# Dilated cardiomyopathy evaluation with Imagenomics: combining multimodal cardiovascular imaging and genetics

**DOI:** 10.1002/ehf2.15307

**Published:** 2025-04-24

**Authors:** Kristian Galanti, Ghaith Sharaf Dabbagh, Fabrizio Ricci, Sabina Gallina, Roberta Giansante, Ron Jacob, Edmond Obeng‐Gyimah, Leslie T. Cooper, Sanjay K. Prasad, David H. Birnie, Andrew P. Landstrom, Selma F. Mohammed, Saidi Mohiddin, Mohammed Y. Khanji, Anwar A. Chahal

**Affiliations:** ^1^ Department of Neuroscience, Imaging and Clinical Sciences G. D'Annunzio University of Chieti‐Pescara Chieti Italy; ^2^ Center for Inherited Cardiovascular Diseases WellSpan Health Lancaster Pennsylvania USA; ^3^ University Cardiology Division, Heart Department SS. Annunziata Polyclinic Chieti Italy; ^4^ Department of Clinical Sciences Lund University Malmö Sweden; ^5^ Institute for Advanced Biomedical Technologies G. D'Annunzio University of Chieti‐Pescara Chieti Italy; ^6^ The Heart and Vascular Institute Lancaster General Health/Penn Medicine Lancaster Pennsylvania USA; ^7^ Perelman Clinical Electrophysiology Section, Cardiovascular Division, Department of Medicine, School of Medicine University of Pennsylvania Philadelphia Pennsylvania USA; ^8^ Division of Cardiovascular Diseases Mayo Clinic Rochester Minnesota USA; ^9^ Department of Cardiology Royal Brompton Hospital London UK; ^10^ Department of Cardiovascular Medicine, National Heart & Lung Institute Imperial College London UK; ^11^ Department of Cardiology University of Ottawa Heart Institute Ottawa Ontario Canada; ^12^ Division of Cardiology, Department of Pediatrics (A.P.L.), School of Medicine Duke University Durham North Carolina USA; ^13^ School of Medicine Creighton University Omaha Nebraska USA; ^14^ NIHR Barts Biomedical Research Centre, William Harvey Research Institute Queen Mary University of London London UK; ^15^ Barts Heart Centre, St. Bartholomew's Hospital Barts Health NHS Trust London UK; ^16^ Barts Heart Centre Barts Health NHS Trust London UK; ^17^ Barts Health NHS Trust Newham University Hospital London UK

**Keywords:** Dilated cardiomyopathy, Multimodal cardiovascular imaging, Genetics, DCM perspectives, Personalized medicine

## Abstract

Dilated cardiomyopathy (DCM) is a clinical diagnosis characterized by the presence of left ventricular dilatation and systolic dysfunction unexplained by abnormal loading conditions or coronary artery disease. However, a broad range of phenotypic manifestations, encompassing isolated scar, DCM with preserved ejection fraction, and overt DCM, should be regarded as a diagnostic classification representing a broad spectrum of underlying aetiologies, including both inherited and acquired heart muscle disorders. A multimodal non‐invasive imaging approach is essential for accurate morpho‐functional assessment of cardiac chambers and is key to establish the cardiac phenotype and to rule out an underlying ischaemic aetiology. Furthermore, advanced imaging techniques enable deep cardiovascular phenotyping and non‐invasive tissue characterization. The aim of this review is to propose a systematic approach to the diagnosis of DCM, emphasizing the importance of genetics and clinical findings for a precise and practical clinical approach. Also, we strive to qualify the role of cardiac imaging in the diagnosis of DCM, particularly on the relevance of novel techniques and clinical utility of actionable parameters to improve current diagnostic schemes and risk stratification algorithms. We further elaborate on the role of cardiac imaging to deliver optimal guidance to aetiology‐based therapeutic approaches, verification of treatment response and disease progression monitoring.

## Graphical abstract

Systematic approach to dilated cardiomyopathy diagnosis. Dilated cardiomyopathy is a clinical diagnosis characterized by the presence of left ventricular dilatation and systolic disfunction unexplained by abnormal loading conditions or coronary artery disease. A multimodal non‐invasive imaging approach is essential for accurate morpho‐functional assessment of cardiac chambers and is key to establish the dilated cardiac phenotype and to rule‐out an underlying ischaemic aetiology. Furthermore, advanced imaging techniques enable deep cardiovascular phenotyping and non‐invasive tissue characterization.

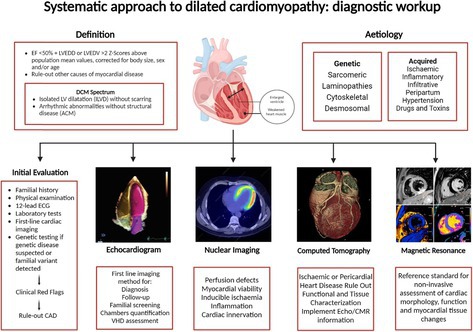

## Introduction

Dilated cardiomyopathies (DCM) represent a prevalent phenotype within heart failure (HF) populations, manifesting a broad range of underlying causes, encompassing both inherited and acquired disorders.^1^ This spectrum includes various aetiologies, ranging from overt DCM to intermediate phenotypes within the DCM spectrum that do not meet definitive diagnostic criteria. Notably, these aetiologies are not mutually exclusive and may coexist, further complicating a strict dichotomous classification. Intermediate phenotypes may present as early arrhythmic disorders without structural diseases, isolated ventricular dilation, and/or hypokinetic non‐dilated cardiomyopathy.^2^ While DCM is defined as the presence of left ventricular (LV) dilatation and global or regional systolic dysfunction unexplained solely by abnormal loading conditions or coronary artery disease (CAD), hypertension, valvular heart disease, and ischaemia frequently coexist with the underlying primary myocardial disorder, influencing disease progression and phenotype expression, particularly in genetically predisposed individuals. In recent years, the increased availability of high‐throughput genetic sequencing to both researchers and clinicians has led to greater recognition of the significance of genetic causes in DCM, with simple Mendelian inheritance accounting for between one‐third and half of the cases.^2^, ^3^ Early detection and detailed characterization of DCM are crucial for aetiology‐oriented therapeutic approaches. It is noteworthy that some forms of DCM are associated with poor prognosis even at a stage where there are few or no symptoms and/or only relatively minor cardiac abnormalities. These individuals, alongside those with a pre‐clinical phase where preventive strategies may be most effective, often exhibit imaging findings that fall outside current diagnostic definitions for DCM, emphasizing the need for clinicians to acknowledge potential limitations in conventional diagnostic imaging thresholds. This review delves into the current evidence regarding the role of genetic investigation and the diagnostic role of non‐invasive multimodality imaging techniques in DCM, underscoring the importance of an aetiology‐oriented treatment and prognosis stratification.

## Definition

The term DCM, although widely adopted, has carried varying interpretations depending on the context. Initially perceived as an idiopathic condition among cardiomyopathy specialists, the classification of cardiomyopathies has expanded over time to include acquired and systemic causes. Conversely, for HF clinical trialists, DCM is typically categorized into ischaemic and non‐ischaemic subtypes. The term non‐ischaemic cardiomyopathy (NICM), particularly prevalent in North America, serves to differentiate this condition from ischaemic aetiologies and may encompass a spectrum of disease stages, from covert to overt DCM.^4^ The European Society of Cardiology (ESC) defines DCM as a heart muscle disease featuring LV dilatation and global or regional systolic dysfunction that are otherwise unexplained by abnormal loading conditions (e.g. hypertension, valvular or congenital heart disease) or coronary artery disease.^5^ A more precise echocardiography‐based definition is LV end‐diastolic diameter (LVEDD) or volume (LVEDV) > 2 Z‐score above population mean values, adjusted for body size, sex, and/or age; meanwhile, LV dysfunction is defined by LV ejection fraction (EF) < 50%.^6^ In 2016, Pinto *et al*. proposed a revised definition, acknowledging the spectrum of DCM phenotype observed, considering that a long asymptomatic phase can frequently occur in the disease process, in conditions of normal LVEF and/or sometimes dilated LV cavity dimensions. Right ventricular (RV) dilatation and/or dysfunction may not be present and are not required for diagnosis. No phenotypic expression and fully expressed DCM can show different intermediate categories: isolated left ventricular dilatation (ILVD) without impairment of EF, early arrhythmic abnormalities without structural disease, and hypokinetic non‐dilated cardiomyopathy. In the 2023 ESC guidelines, a new term, non‐dilated left ventricular cardiomyopathy (NDLVC), was introduced, characterized by the presence of non‐ischaemic LV scarring or fatty replacement, regardless of the presence of wall motion abnormalities.^2^, ^5^ For the purposes of this review, the term DCM used henceforth refers to the spectrum of NDLVC and over DCM.

## Epidemiology

Reclassifications and evolving definitions of DCM over recent decades have led to variability in data regarding its accurate epidemiology.^7^ Although previously perceived as a rare or orphan disease, estimates of DCM incidence, typically manifesting between the third and fourth decades of life, range from 1 in 2500 to 1 in 250 individuals, often exhibiting a male predominance (1.4:1 male‐to‐female ratio).^8^ Recent reviews suggest that DCM prevalence may range from 1 in 250 to 1 in 400 individuals within the population.^4^ Furthermore, familial DCM represents 30–50% of DCM cases, with approximately 30–40% of these cases attributable to identifiable genetic causes.^9^

## The role of genetics

**Figure 1:**
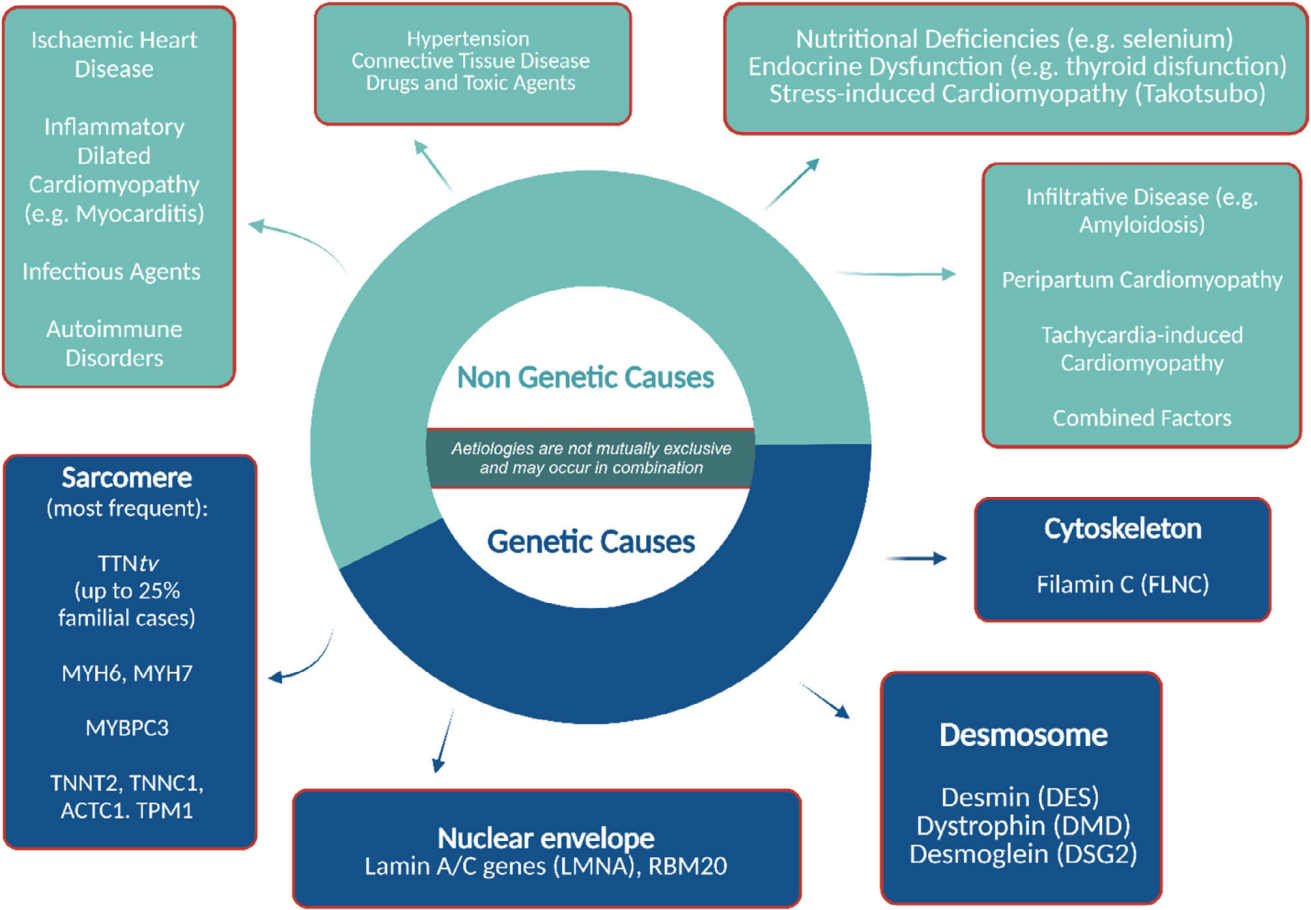
Principal aetiologies of DCM. This figure endeavours to delineate the broad spectrum of aetiologies underlying DCM, primarily categorized into genetic and non‐genetic factors. The understanding of DCM's aetiology and natural progression has significantly advanced in recent decades. Various underlying causes leading to left ventricular dysfunction can ultimately manifest as DCM. BAG3, BAG Cochaperone 3; DCM, dilated cardiomyopathy; RBM20, RNA binding motif protein 20; TTN, titin gene; VTs, ventricular tachycardias.

**Figure 2:**
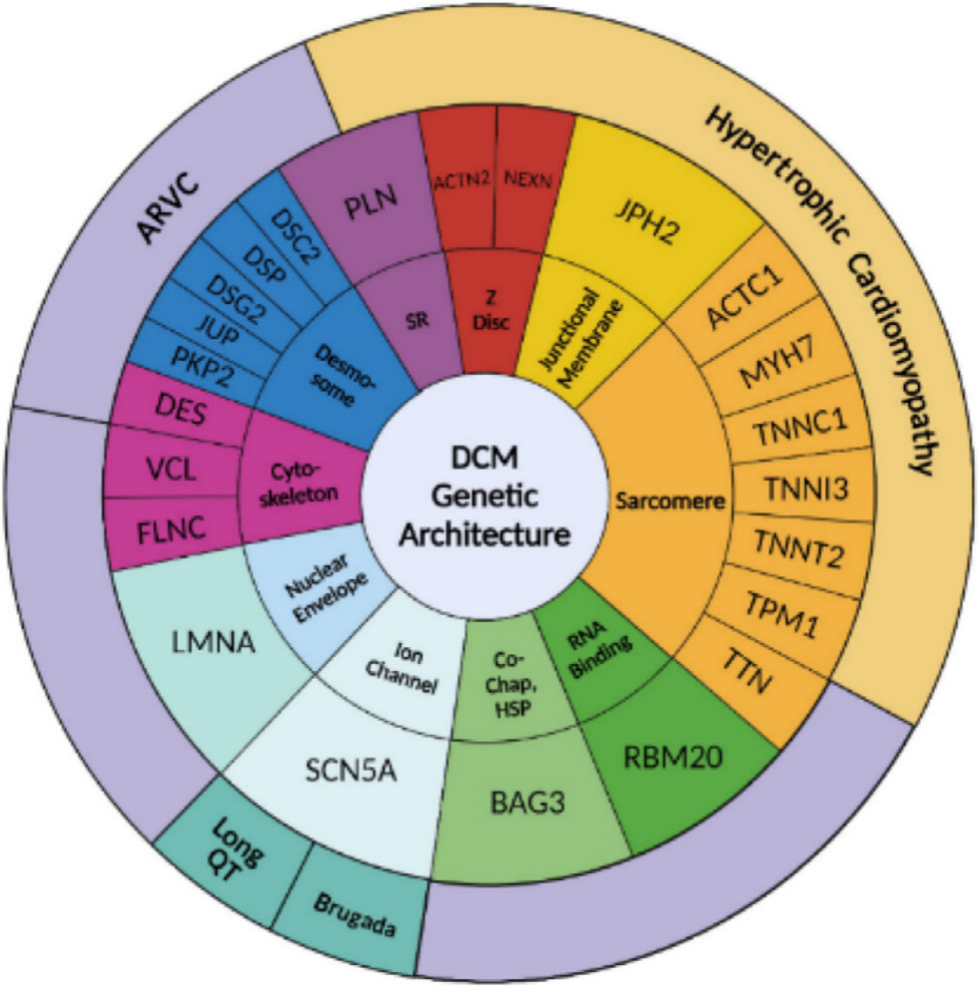
Genetic burden of DCM. The genetic contribution to DCM accounts for approximately 30–40% of cases, with around 40% of these cases attributed to rare variants in over 60 genes that these figure aim to summarize. Different inheritance patterns have been identified, ranging from the prevalent autosomal dominant to fewer common patterns, such as X‐linked, autosomal recessive, and matrilinear transmission [adapted from Jordan *et al*.^1^].

The causes of DCM can be divided into genetic and non‐genetic (*Figure* *1*), with the genetic yield of DCM encompassing about 30–40% of the cases^10^ and approximately 40% of the genetic causes being linked to rare variants in over 60 genes^11^ (*Figure* *2*). Various patterns of inheritance have been recognized, including the most common autosomal dominant, as well as X‐linked, autosomal recessive and matrilinear transmission.^12^, ^13^, ^14^ Also, putative pathogenic variants can be identified in some cases of sporadic DCM. Given the growing importance of genetics in the characterization of patients with DCM, the role of multidisciplinary teaming becomes pivotal. Such a team should include specialists with expertise in genetic testing methodology, variant interpretation, and clinical application, ideally operating within a specialized inherited cardiovascular disease service or a cardiomyopathy service or an equivalent network.^15^

### Genetic causes

Genetic testing was previously recommended only if there was a history of at least two affected family members. However, falling costs and greater accessibility have led to this being a class I recommendation where non‐ischaemic DCM is diagnosed.^5^ In fact, as expressed in the Expert Consensus Statement on the State of Genetic Testing for Cardiac Diseases, genetic testing is useful in all DCM patients, recommended in patients with the highest yield of pathogenic variant screening and should be considered even in the absence of familial context or associated clinical features (<60 years of age).^16^ Even though there are still difficulties related to variants of uncertain significance (VUS), high‐throughput sequencing with targeted sequencing panels of genes is the most cost‐effective approach and recommended technique. VUS are often segregates into the investigated family, presenting significant challenges in interpretation^17^; however, this uncertainty can be mitigated through in silico prediction tools, co‐segregation analysis, high‐throughput in vitro functional validation, and specialized web‐based models that utilize amino acid‐level signal‐to‐noise analysis to aid in differential diagnosis.^18^ Collaboration with the Cardiogenetic Team will be key to determine, through consultation with specific databases (e.g. ClinVar, Franklin Genoox, VarSome), whether the specific mutation has undergone reclassification as pathogenic or not. The most widely used classification system of genetic variants is that of the American Council of Medical genetics and Genomics (ACMG) although others (such as the ABC system) exist.^19^, ^20^ While the ACMG classification is a lab‐based classification, clinical interpretation and classification may be different. Clinicians are encouraged to work with the laboratories to provide new information, which may lead to reclassification of the VUS into benign or pathogenic categories. Decision‐making is on benign or pathogenic and not VUSs. The most prevalent variants in autosomal dominant DCM are located within genes encoding for sarcomeric proteins and the nuclear envelope protein lamin A/C. Truncating variants in the *TTN* (*TTNtv*) gene are identified in 20–25% of DCM patients. Variants in genes encoding for other sarcomeric proteins [e.g. myosin heavy chain (*MYH7*), cardiac troponin T (*TNNT2*), α‐tropomyosin (*TPM1*), cardiac troponin C (*TNNC1*), cardiac troponin I (*TNNI3*)], are considered to be disease‐causing in up to 10% of patients with DCM.^4^ For this reason, panels should include validated genes in DCM, with most recurrent genes such as those aforementioned.^16^ Also, genetic testing can be oriented by the presence of a particular extra‐cardiac phenotype as for example neuromuscular diseases, mitochondrial diseases, or congenital syndromes.^21^ This recommendation for diagnostic genetic testing extends to children at higher risk of DCM due to syndromes associated with cardiomyopathy. Children often present with recessive disease or compound heterozygosity, and certain inborn errors of metabolism, exhibiting aggressive phenotypes and limited treatment options not applicable to adult DCM, such as enzyme replacement therapy in Pompe disease.^22^, ^23^

### Genetic testing: Definitions and timing

Three primary recommendations guide the evaluation of a patient with primary cardiomyopathy: (I) obtaining a comprehensive family history spanning at least three generations; (II) advising clinical screening for cardiomyopathy in at‐risk first‐degree relatives; and (III) referring patients with genetic, familial, or other unexplained forms of cardiomyopathy to expert centers.^22^ Consequently, a minimum three generational family tree undergoing genetic testing should be considered, as this offers an opportunity for cascade family screening and preventive measurements that might otherwise be missed. Familial DCM typically follows autosomal dominant inheritance, although recessive and X‐linked inheritance patterns have been documented.^24^ The familial risk of DCM among relatives of affected individuals depends on the degree of relationship, being highest in full siblings, but still notable in second‐degree and third‐degree relatives.^25^ Diagnostic or confirmatory testing is performed in individuals with suspected cardiomyopathy, while cascade or predictive testing is conducted in clinically unaffected relatives of the proband. Genetic testing holds potential benefits for all patients with DCM, irrespective of age or underlying causes.^5^ In cases where probands test negative for genetic variants, echocardiography and ECG screenings are recommended in all first‐degree relatives around the age of 10, with subsequent screenings scheduled every 2–3 years if cardiovascular tests yield normal results, annually if minor abnormalities are detected and every 6 months during periods of rapid growth, such as adolescence. For genotype positive probands, cascade testing is performed, and only genotype‐positive patients will undergo follow‐up. It is important to note that genetic surveillance in children should begin either upon confirmation of genotype positivity through familial cascade testing or at the time of proband's diagnosis if genotype negativity is confirmed or if cascade testing for the child is not pursued or declined.^22^, ^23^ There are currently no definitive indications on when to conclude screening programmes, though a suggested upper age of 65 years has been proposed.^26^ Screening intervals may also be adjusted based on the specific course of DCM subtypes. Genetic panels result for cardiomyopathies typically ranges from 4 to 6 weeks, depending on the scope of the panel, the complexity of variant interpretation, and the prioritization protocols of the testing laboratory. Notably, patients with *LMNA* warrant particular attention due to the propensity for early‐onset atrioventricular block, supraventricular or ventricular arrhythmias, and progressive DCM. Additionally, other genes assume significance in patients without overt DCM phenotypes, including *TTN*tv, *DSP*, *FLNC*, and RNA binding motif protein 20 (*RBM20*).^27^

## Clinical features

### Diagnostic work‐up

**Figure 3:**
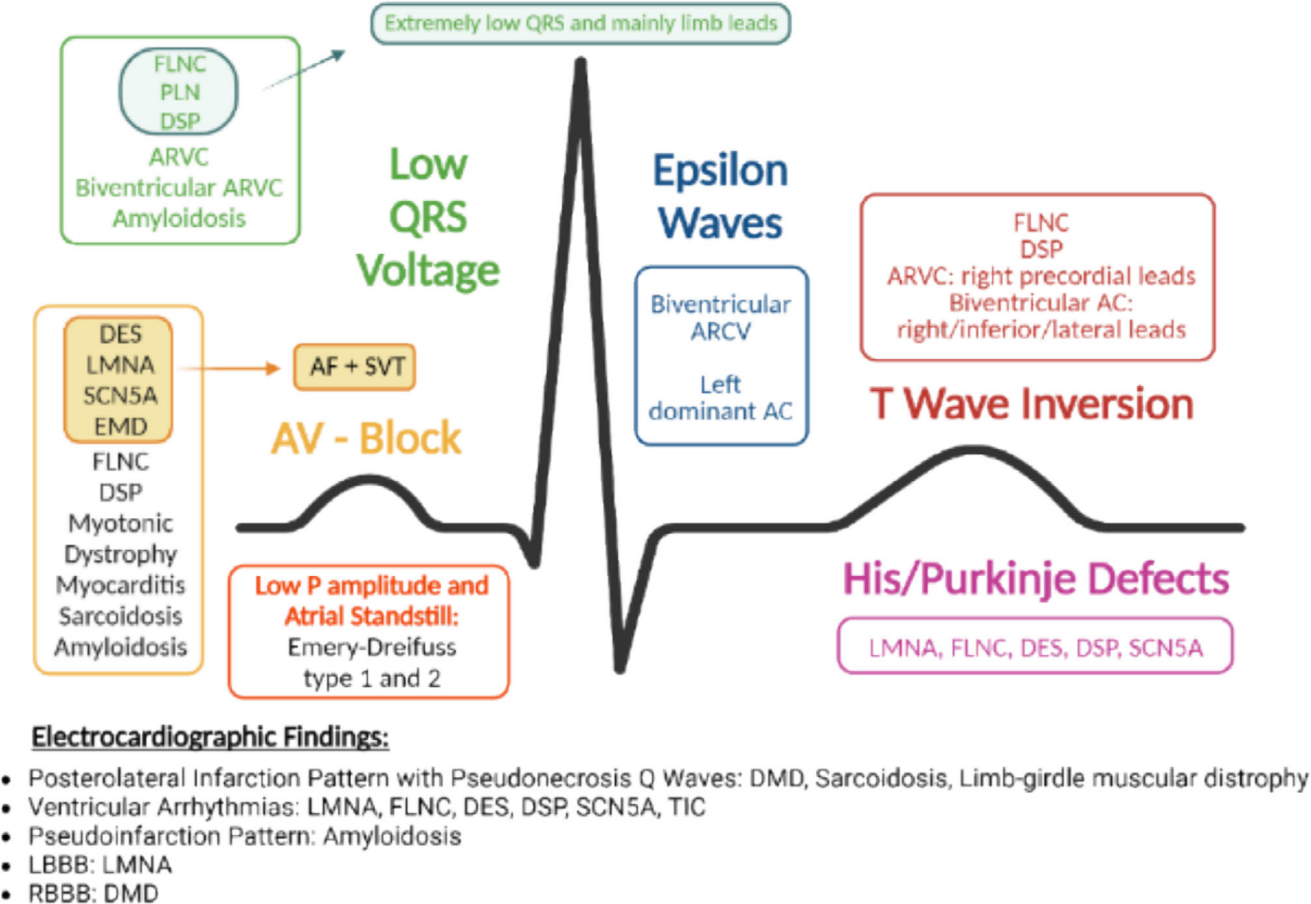
ECG red flags and abnormalities in genetic and acquired forms of DCM. ECG constitutes a central investigative modality in the diagnostic work‐up of DCM, alongside personal and family history, age, physical examination, and laboratory parameters. This figure highlights specific electrocardiographic ‘red flags’ that may prove beneficial in the diagnostic direction of DCM. AC, arrhythmogenic cardiomyopathy; AF, atrial fibrillation; ARVC, arrhythmogenic right ventricular cardiomyopathy; DES, desmin gene; DMD, dystrophin gene; DSP, desmoplakin gene; EMD, Emery–Dreifuss; FLNC, filamin C gene; LBBB, left bundle branch block; LMNA, lamina A/C gene; PLN, phospholamban gene; RBBB, right bundle branch block; SCN5A, sodium voltage‐gated channel alpha subunit 5; SVT, supraventricular tachycardia; TIC, tachycardia‐induced cardiomyopathy.

**Figure 4:**
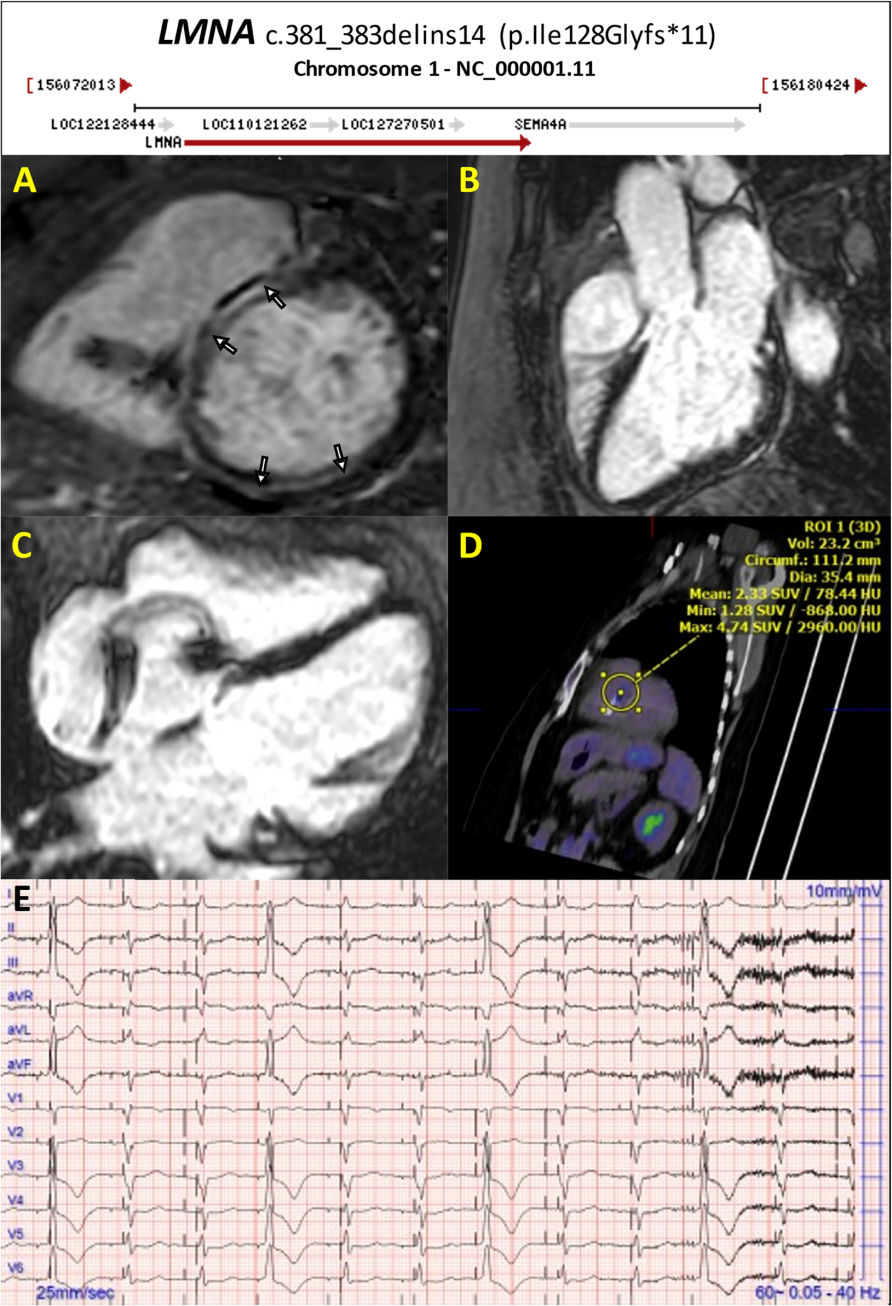
DCM in patient with permanent pacemaker and LMNA mutation. Permanent pacemaker in DCM with LVEF 45%. CMR shows mid myocardial LGE involving the basal anteroseptal and posterior wall, FDG+, paroxysmal AF. Genetics: LMNA c.381_383delins14 (p.Ile128Glyfs*11). (A) CMR LGE short axis. (B) CMR LGE 3ch. (C) CMR LGE 4ch. (D) CT‐PET active myocardial inflammation involving the basal septum, max SUV 4.7. (E) ECG electronic atrial/ventricular pacemaker (due to complete heart block) with occasional PVC fusion. AF, atrial fibrillation; DCM, dilated cardiomyopathy; PVC, premature ventricular contraction.

A systematic approach is essential for the comprehensive evaluation, aetiological determination, management, and prognostication of DCM. Initial assessment requires detailed personal and familial history, age‐specific considerations, physical examination, 12‐lead and Holter‐ECG monitoring, laboratory investigations, and first‐line cardiac imaging. Recognition of ‘red flags’ (*Table* S1) guides subsequent diagnostic and prognostic steps, prompting advanced assessments including CMR, endomyocardial biopsy (EMB), and genetic analysis.^28^, ^29^ Laboratory assessment include comprehensive blood analyses, thyroid function tests, serological screenings, natriuretic peptides, and novel biomarkers reflecting heart strain, myocyte injury, and oxidative stress. Specialized metabolic testing may be warranted in paediatric cases, evaluating lactate, pyruvate, and metabolic profiles.^5^ ECG abnormalities can be noticed in up to 80% of patients with DCM and may offer insights into specific genetic or acquired aetiologies^30^, ^31^ (*Figure* *3*). Also, considering that cardiovascular structures adapt to metabolic demands, cardiopulmonary exercise test aids in differentiating physiological from pathological cardiac enlargement.^32^ Genetic testing is important for both early disease identification and prognostic stratification, guiding interventions, including ICD therapy in high‐risk individuals harbouring pathogenic variants (e.g. *TTNtv*, *LMNA*, *and RBM20*; *Figure* *4* and *Figures* *S2* *–* *S27*).^5^ Besides, innovative tools, such as the Madrid Genotype Score, improve the efficiency of genetic testing selection, thereby optimizing patient management [https://madriddcmscore.com/].^33^ *Figure* *S28* describes the cardiac genetic service specialists involved in this management. When non‐invasive techniques fail to establish an aetiological diagnosis, EMB plays a crucial role in identifying myocardial inflammation and infiltrative or storage diseases, thereby guiding targeted therapy and refining prognostic stratification.^5^

### Differential diagnosis

The interplay between genetic predisposition and environmental factors is central to DCM pathogenesis. Given its heterogeneity—encompassing both acquired and genetic aetiologies—rigorous exclusion of secondary causes is essential before diagnosing idiopathic DCM.^34^ This includes ruling out ischaemic aetiology, where advanced imaging modalities such as cardiac computed tomography (CCT), nuclear imaging, and cardiovascular magnetic resonance (CMR) are invaluable tools. A registry study of 3023 consecutive CAD patients undergoing contrast‐enhanced CMR for the assessment of ventricular function and scar identified coexisting NICM in one out of six cases, highlighting its under‐recognition. Notably, patients with CAD and concurrent NICM had worse long‐term outcomes compared to those with isolated ischaemic cardiomyopathy. Coincidental NICM in patients with CAD was associated with worse long‐term outcomes, compared to patients manifesting solely ischaemic cardiomyopathy.^35^ Additionally, conditions predisposing to DCM must be considered, including arrhythmia‐induced cardiomyopathy (AiCM), peripartum cardiomyopathy (PPCM), cardiotoxic effects of cancer treatments, myocarditis, iron overload, and exposure to toxins, like alcohol and cocaine.^36^, ^37^, ^38^, ^39^ Notably, the presence of alternative causes does not preclude the identification of pathogenic mutations, given the marked genetic and allelic heterogeneity of cardiomyopathies, characterized by incomplete penetrance and variable expressivity. Consequently, individuals with putative genetic variants may either develop DCM or remain unaffected throughout their lives, with genetic predisposition contributing to disease manifestation. For instance, *TTNtv* are associated with alcoholic cardiomyopathy and with worse LVEF in DCM patients who excess alcohol consumption.^5^ Early detection of DCM is possible, particularly in asymptomatic individuals, by assessing risk factors such as familial recurrence and uncontrolled cardiovascular risk factors. Detailed evaluation of LV size, diastolic function, and global longitudinal strain (GLS) aids in defining the preventive and therapeutic strategies, facilitating lifestyle modifications or medical interventions that can significantly alter disease trajectory and reduce morbidity and mortality.^40^, ^41^ An expert consensus document proposed by the ESC recommend comprehensive diagnostic criteria for relatives of familial DCM patients, integrating multiple imaging methods and 12‐lead ECG. Specific imaging criteria, including LVEF and LV dilatation, are considered as major criteria, whereas others, as an abnormal regional wall motion in the absence of conduction defects and non‐ischaemic late gadolinium enhancement (LGE) identified by CMR, are considered minor ones^42^ (*Table* *S2*).

### Inflammatory‐mediated myocardial injury

Myocarditis, a differential diagnosis in DCM patients, exhibits a broad clinical spectrum, from meeting revised Pinto criteria to potentially progressing to a DCM‐like phenotype^43^ or arrhythmogenic cardiomyopathy.^44^ While often resolving spontaneously, it poses a significant risk of SCD in young individuals, with inflammation‐induced scarring contributing to extensive LV remodelling.^45^ The condition presents in two main forms: acute myocarditis (AM), characterized by a short duration from symptom onset to diagnosis (generally less than 1 month), and chronic inflammatory cardiomyopathy (infl‐CMP), where myocardial inflammation is established alongside DCM or NDLVC.^46^, ^47^ Anamnestic clues, such as recurrent myocarditis or hypersensitivity conditions, subtle electrocardiographic abnormalities (e.g. low voltage or fragmentation of QRS in peripheral leads, minor conduction disturbances, and non‐specific ST‐T abnormalities), low‐grade persistent elevation of troponin, and unresponsiveness to standard HF treatment should prompt investigation for an inflammatory aetiology (*Table* *S3*). Notably, the boundary between inflammatory and genetic cardiomyopathy is blurred, as they can coexist: An early pathological series of arrhythmogenic right ventricular cardiomyopathy (ARVC) showed lymphocytic myocarditis in almost 2/3 of hearts,^48^ and a case series documented patients with acute chest pain, troponin elevations, and imaging typical of myocarditis who were found to have desmoplakin (*DSP*) cardiomyopathy. Also, a recent clinical report by Peretto *et al*. regarding the overlap between myocarditis and genetic cardiomyopathies shows that multimodal imaging can help in discriminating specific genotypes and identifying myocardial inflammation, proven using endomyocardial biopsy.^49^ An evaluation and management approach incorporating multimodality imaging is crucial for immune‐mediated systemic inflammatory diseases. Early diagnosis and non‐invasive monitoring of cardiac involvement, facilitated by novel technologies, can enhance patient selection, provide surrogate endpoints, and thereby improve clinical outcomes.^50^

### Arrhythmia‐induced cardiomyopathy

‘Arrhythmia‐induced cardiomyopathy’ is increasingly replacing ‘tachycardia‐induced or ‐mediated cardiomyopathy’, because of the growing recognition that a tachycardia, atrial fibrillation or PVCs, can induce a sometimes reversible cardiomyopathy.^51^ Part of the challenge in assessing morphological phenotypes lies in discerning between AiCM and DCM with arrhythmias as part of the clinical spectrum. AiCM can occur in the setting of AF with rapid ventricular response (RVR), high PVC burden, left bundle branch block (LBBB), and even high premature atrial contraction (PAC) burden.^52^ Presentation can vary widely from asymptomatic to decompensated HF. Mechanisms contributing to AiCM include dyssynchrony, altered myocardial blood flow, AV dissociation, sympathetic dysregulation, calcium overload, and impairment of excitation–contraction coupling, resulting in tissue, myocyte, and electrical remodelling.^51^ Reversibility is the hallmark of AiCM, distinguishing it from DCM with arrhythmias, and thus suppression of the arrhythmia can help distinguish this. It should be noted that MMI can help identify underlying non‐reversible causes.^53^ Additionally, there are some imaging features that may help distinguishing PVC‐mediated cardiomyopathy from DCM with PVCs using imaging features (*Table* *S4*).

## Role of non‐invasive multimodality imaging

**Figure 5:**
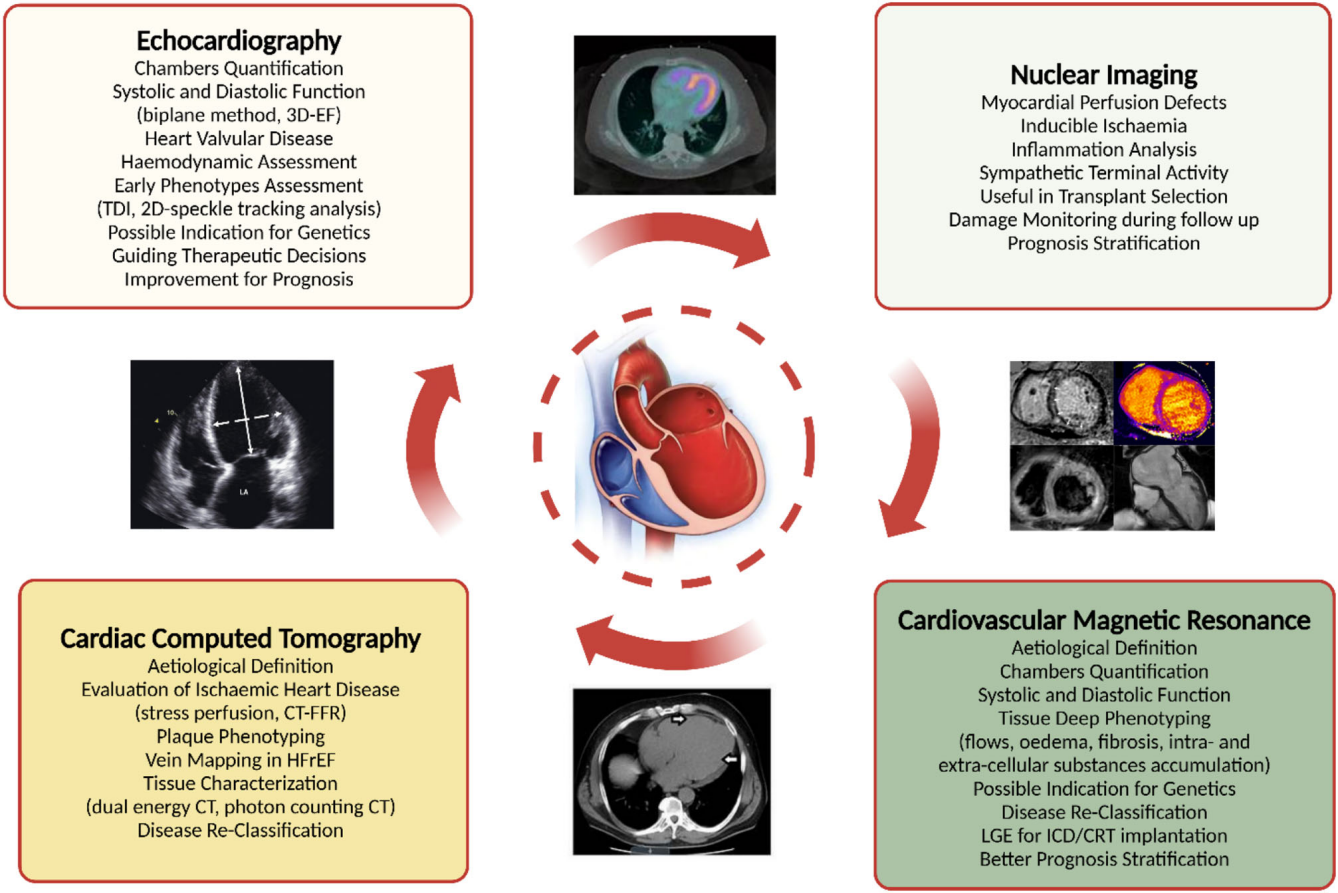
The importance of multimodality cardiovascular imaging for DCM. Multimodal cardiovascular imaging can be used for the diagnosis, as guidance to treatment (e.g. ICD, CRT, LVAD) and for follow‐up of DCM. The appropriateness of use for each technique may be dependent on the mode of presentation, the stage of the DCM, the symptomatic status, and the need to perform a screening. A multimodal imaging approach is key to confer a broader point of view on this condition, from revealing preclinical signs of the disease to the definition of an aetiology‐based therapeutic management. CRT, cardiac resynchronization therapy; CT, computed tomography; HFrEF, heart failure with reduced ejection fraction; ICD, implantable cardioverter defibrillator; LGE, late gadolinium enhancement; MRI, magnetic resonance imaging; PET/SPECT, positron emission tomography/single‐photon emission computed tomography.

Cardiovascular imaging is key for diagnosis, guiding treatment and monitoring DCM. Its appropriateness depends on presentation urgency, disease stage, symptoms, and screening needs.^42^ A multimodal imaging strategy offers comprehensive insights, from detecting preclinical signs to tailoring aetiology‐based therapy (*Figure* *5*).

### Echocardiography

Two‐dimensional (2D) transthoracic echocardiography (TTE) with Doppler is the first‐line imaging method for diagnosing, monitoring, and screening familial cases of DCM, offering comprehensive data on chamber dimensions, systolic and diastolic function, and valvulopathies, in a broadly available, non‐invasive, and cost‐effective manner.^54^ DCM is echo‐defined by LVEDD or LVEDV exceeding 2 Z‐score beyond population mean values, corrected for body size, sex, and/or age, with concomitant LV dysfunction defined by LVEF < 50%.^5^ A value of predicted LVEDD > 112% (>2SD) is a diagnostic criterion for DCM, and a value > 117% (2SD + 5%) increases specificity.^55^ Accurate quantification of LV function is key to guide therapeutic decisions, including ICD, resynchronization therapy, or chemotherapy discontinuation.^56^ Despite its variability, the apical biplane method of discs remains the preferred technique for LV volumes and EF assessment, although consideration of alternative methods such as three‐dimensional (3D)‐EF is warranted for more reliable and improved reproducibility.^57^ Notably, 3D‐TTE may overcome the limitations related to 2D‐TTE with respect to LV volumes and EF estimates but requires good quality images. DCM typically manifests with diffuse LV hypokinesia, but regional wall motion abnormalities may be present and should be distinguished from CAD‐related wall motion abnormalities.^55^ Also, LV eccentric hypertrophy and LV diastolic dysfunction along with secondary mitral regurgitation (MR) may be present as a result of the apical tethering of leaflets, annular dilatation, and/or ventricular dyssynchrony.^58^ Early DCM detection is facilitated by tissue Doppler imaging (TDI)^40^, ^59^, ^60^ and GLS analysis by 2D speckle tracking echocardiography, which is the most commonly studied parameter for the detection of preclinical disease.^61^, ^62^ Abnormal circumferential and radial deformation parameters, as well as abnormal torsion, have also been described in preclinical DCM patients. Differential diagnosis with myocarditis, especially in cases with preserved LVEF, entails recognizing specific echocardiographic findings indicative of AM: increased wall thickness, mild segmental hypokinesia, in particular in the inferior and infero‐lateral walls, diastolic dysfunction, abnormal TDI, mild RV dysfunction, pericardial effusion, pericardial thickening, effuse‐constrictive physiology, and abnormal myocardial echogenicity.^46^ Long‐term follow‐up echocardiography aids in prognostic stratification, focusing on the left or right ventricular reverse remodelling, eventual MR, and/or LV restrictive filling pattern improvements. Despite its utility, TTE limitations include image quality issues and tissue characterization deficiencies.^59^ Further details on echocardiographic assessment and follow‐up protocols for DCM are available in *Table* *S5*.

### Nuclear imaging

Nuclear imaging techniques are valuable adjuncts in diagnosing DCM, offering insights into myocardial perfusion defects, viability, inducible ischaemia,^59^ inflammation, and cardiac innervation through radio‐labelled compounds.^42^ Single‐photon emission computed tomography (SPECT) is crucial for excluding ischaemia and providing prognostic information. Positron emission tomography (PET), alone or combined with computed tomography (CT), is a valid but expensive alternative for detecting myocardial ischaemia^59^ and evaluating sympathetic nerve terminal activity, offering prognostic value in both ischaemic and non‐ischaemic cardiomyopathy.^63^, ^64^ Nuclear imaging complements other imaging modalities in identifying uncommon DCM aetiologies, including sarcoidosis or amyloidosis.^65^ Radionuclide imaging serves a unique role in diagnosing cardiac amyloidosis, with ^99m^Tc‐labelled diphosphonate and pyrophosphate (bone‐avid) compounds demonstrating high sensitivity and specificity, aiding in early *ATTR* cardiac amyloidosis detection. Radionuclide ventriculography assesses LV function without any geometrical assumptions but suffers from high intra‐ and inter‐observer variability.^66^ In the context of AM or infl‐CMP, PET is not standard, but it may be considered for stable patients unable to undergo CMR or those with suspected systemic autoimmune diseases.^67^ PET is particularly useful in diagnosing and monitoring cardiac sarcoidosis, revealing hypermetabolic mediastinal and hilar lymph nodes, differentiating sarcoidosis from other autoimmune disease with cardiac involvement (e.g. vasculitis).^68^ This technique provides a tool to monitor the progression of damage and its regression in response to immunosuppressive therapy. In this context, 18F‐FDG CT‐PET improves quality in the diagnostic pathway: CMR is commonly done first so that certain pathologies as sarcoidosis can be excluded and, if other abnormalities are encountered, progressing to 18F‐FDG CT‐PET is appropriate. If CMR cannot be performed, direct 18F‐FDG CT‐PET is helpful in suspected cases of infl‐CMP and has a role in surveillance and response assessment to therapy.^69^ Serum beta‐hydroxybutyrate levels help assess LV suppression during PET studies.^70^ Also, while PET enhances diagnostic pathways, its role in evaluating microvascular dysfunction remains largely investigational, with H_2_^15^O or ^13^NH_3_ dipyridamole (or regadenoson) use still mainly confined to research purposes.^5^ Various radionuclide tracers are employed for heart pathologies, detailed in *Table* *S6*, although their direct diagnostic role in DCM is still limited.^29^

### Cardiac computed tomography

Cardiac CT (CCT) has emerged as a promising adjunct to CMR for assessing both functional and tissue characteristics, expanding its utility beyond coronary anatomy and plaque phenotyping evaluations.^71^ While invasive coronary angiography (ICA) remains the gold standard for DCM patients with intermediate‐to‐high pre‐test CAD probability, CCT serves as a valuable non‐invasive alternative, particularly in subjects with low‐to‐intermediate CAD^42^ likelihood or when constrictive pericarditis^65^ or congenital anomalies in children and adolescents are suspected.^5^ CCT effectively rules out CAD in patients with LV systolic dysfunction, given its high negative predictive value, and ability to detect myocardial inflammation.^42^, ^72^ Emerging techniques like stress perfusion CT and CT‐derived fractional flow reserve (CT‐FFR) offer promising avenues for ischaemia assessment.^73^, ^74^ CCT demonstrates high diagnostic accuracy for severe coronary stenosis identification in patients with reduced LVEF,^74^ possibly due to optimal heart rate control and reduced cardiac motion obtained with targeted medical therapy. The ESC recognizes the role of CCT in excluding significant CAD in newly diagnosed DCM patients.^75^ Despite its potential, technical limitations hinder CCT non‐coronary application, including (I) non‐negligible radiation dose, (II) contrast medium requirements, and (III) limited post hoc analysis for biventricular volumes and function. Dual‐energy CT^42^ and photon‐counting CT represent promising advancements, enabling myocardial fibrosis identification with reduced contrast and improving dose efficiency and spatial resolution, respectively.^76^, ^77^ Further research is warranted to delineate photon‐counting CT role in cardiovascular disease, underscoring the need for ongoing investigation into CCT evolving applications.^78^

### Cardiovascular magnetic resonance

The recent advancements in CMR imaging have revolutionized the non‐invasive characterization of cardiomyopathies, establishing it as the reference standard for cardiac morphology, function, and tissue characterization. CMR enables accurate quantification of biventricular parameters, intra‐ and extra‐cardiac flows, detection of myocardial oedema, fibrosis, and intracellular/extracellular substances accumulation (such as fat, iron, and amyloid), offering invaluable insights into disease aetiology and prognosis.^79^ It is now a fundamental tool for diagnosing and stratifying patients with LV dysfunction, with contrast‐enhanced CMR reaching class I—level of evidence B recommendation for the initial evaluation of patients with cardiomyopathy.^5^ Overcoming limitations of other imaging modalities, CMR is particularly helpful in patients with non‐diagnostic or suboptimal 2D‐echo imaging and should be considered in high‐risk families where DCM diagnosis remains uncertain, significantly influencing patients' management.^80^ Despite concerns about gadolinium contrast agents, their safety profile, with negligible risk of complications such as nephrogenic systemic fibrosis, has been affirmed by regulatory authorities.^81^ The approval of newer agents, such as gadopiclenol, underscores the continued advancements and safety of CMR imaging in clinical practice. Despite the increasing demand and availability, access to CMR remains geographically variable.^82^ Therefore, in such cases, the integration of multiple imaging modalities is pivotal, with echocardiography playing a key role as the first‐line tool.

### CMR: Tissue characterization and deep phenotyping

**Figure 6:**
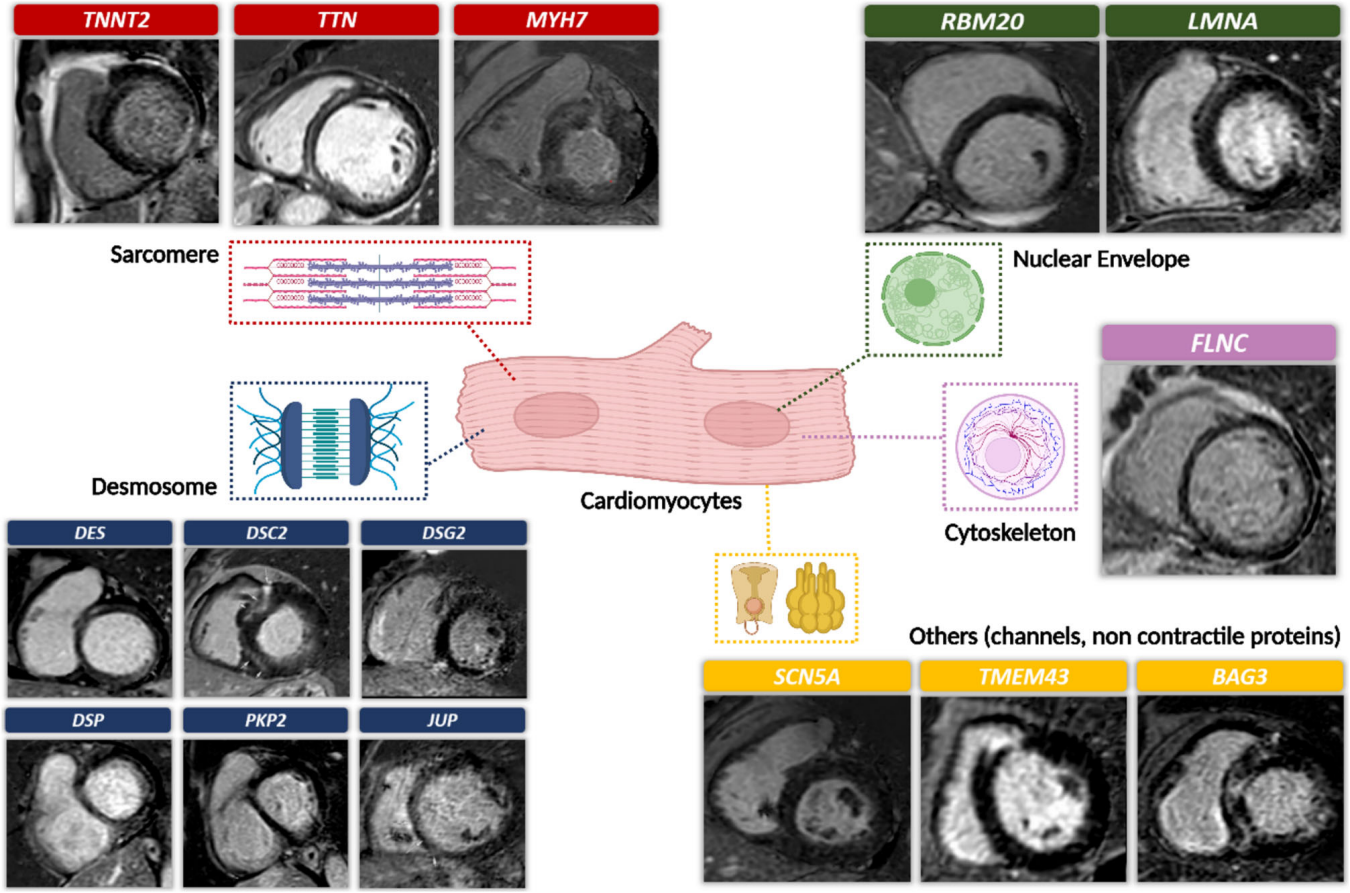
The pivotal connection between genetics and imaging: the role of the LGE. The LGE technique is currently the non‐invasive gold standard for the identification and the quantification of myocardial scar and is used to evaluate replacement fibrosis, providing key information with respect to aetiology and clinical outcome. Here is a summary on the most relevant genetic disorders and their CMR phenotype.

CMR plays a pivotal role in assessing myocardial longitudinal strain and tissue characterization using various sequences, including early gadolinium enhancement, T2‐ and T1‐weighted imaging, or mapping and LGE.^83^ Its safety profile, absence of radiation exposure, and ability to provide serial scans in adults and children make CMR the preferred modality for serial scans. The LGE technique is currently the non‐invasive gold standard for identifying and quantifying myocardial scar, aiding in aetiological classification (e.g., ischaemic vs. NICM) and prognostication (*Figure* *6*). Ischaemic heart disease typically exhibits subendocardial/transmural LGE in coronary artery vascular territories (ischaemic pattern), whereas non‐ischaemic cases can present with diverse non‐ischaemic LGE patterns (e.g., mid‐wall, sub‐epicardial, or patchy distribution). LGE presence, patterns, and quantification inform risk stratification for malignant VAs and predict LV reverse remodelling (LVRR).^84^, ^85^ Stress perfusion CMR complements LGE, distinguishing ischaemic from NICM with high diagnostic accuracy. Mapping techniques enable myocardial tissue characterization beyond LGE and, together with the assessment of native T1, T2, and T2*, offers insights into tissue properties. Pre‐ (or native) and post‐contrast T1 mapping, coupled with actual or synthetic haematocrit, enables extracellular volume (ECV) quantification. Native T1 mapping and ECV have been employed to quantify cardiac amyloid burden both in *ATTR* and in *AL* amyloidosis^86^: without known causes to expand the cardiac interstitium, such as oedema or amyloid deposits, ECV is a valuable biomarker of interstitial fibrosis.^87^ T2 short‐tau inversion recovery (STIR) mapping identifies myocardial oedema, indicative of active inflammation, while T2* mapping is a method of choice for the non‐invasive assessment and quantification of cardiac iron.^88^ For this reason, T2* values can be useful in DCM differential diagnosis pathway^59^: a T2* value of the myocardium ≤10 ms is associated with severe iron overload, and 98% of thalassemic patients with a T2* in this range developed overt HF at 1‐year follow‐up.^88^ CMR is indispensable in detecting cardiac sarcoidosis, characterized by variable patterns of LGE but typically: basal mid‐wall and/or epicardial enhancement, septum and the lateral wall involvement and nodular mid‐wall hyperintense foci on oedema‐sensitive sequences, as well as areas of focal myocardial thickening.

### CMR: Inflammation‐related and arrhythmogenic DCM phenotypes

**Figure 7:**
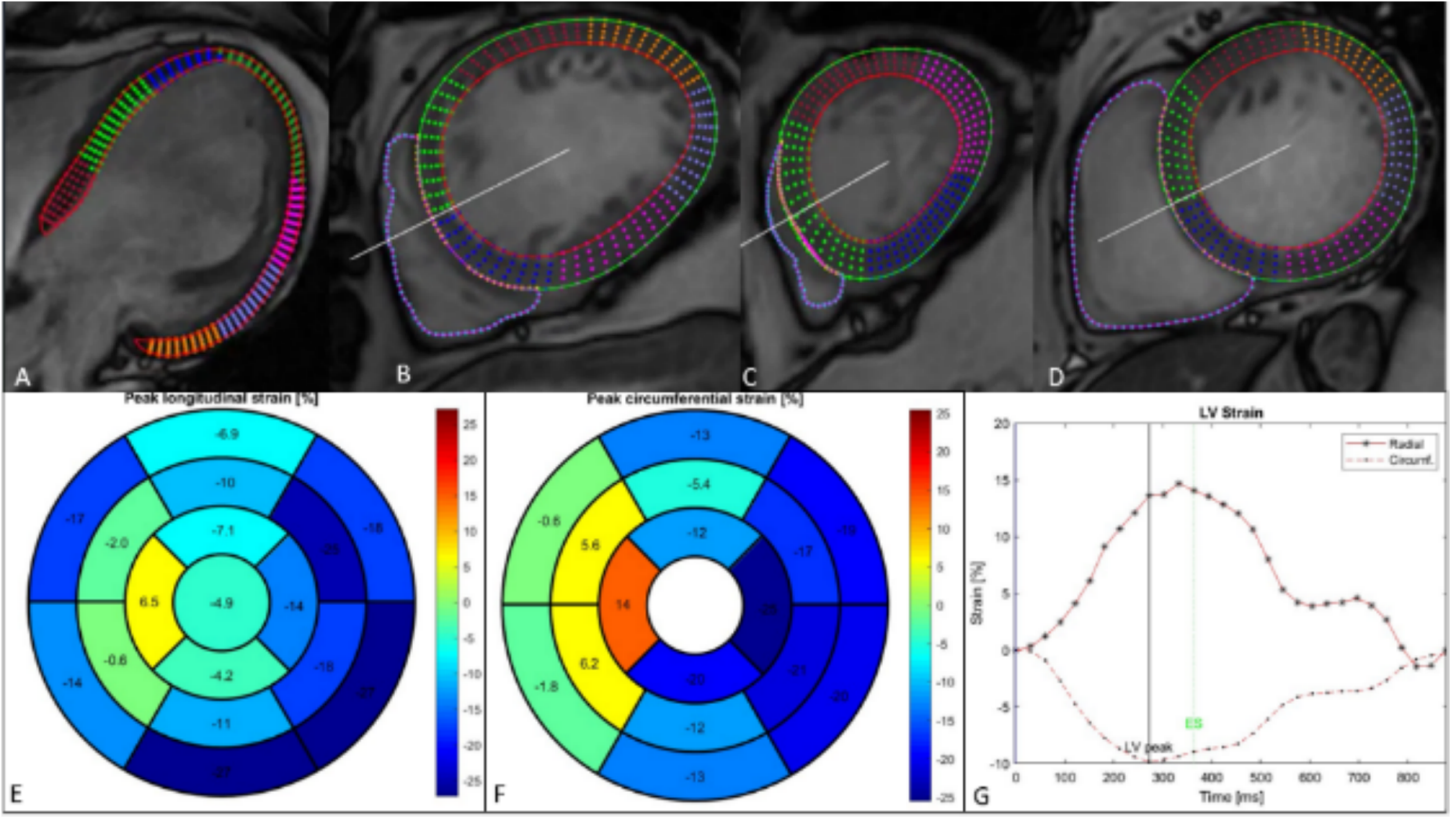
CMR feature‐tracking strain analysis in DCM patient with complete LBBB. (A) LV focused Cine 4Ch‐View strain analysis. (B–D) LV focused Short Axis Cine strain analysis. (E) Long Axis Cine Peak Longitudinal Strain. (F) Short Axis Cine Peak Circumferential Strain. (G) Short Axis Cine LV Strain. CMR, cardiac magnetic resonance; DCM, dilated cardiomyopathy; LBBB, left bundle branch block; LV, left ventricle.

**Figure 8:**
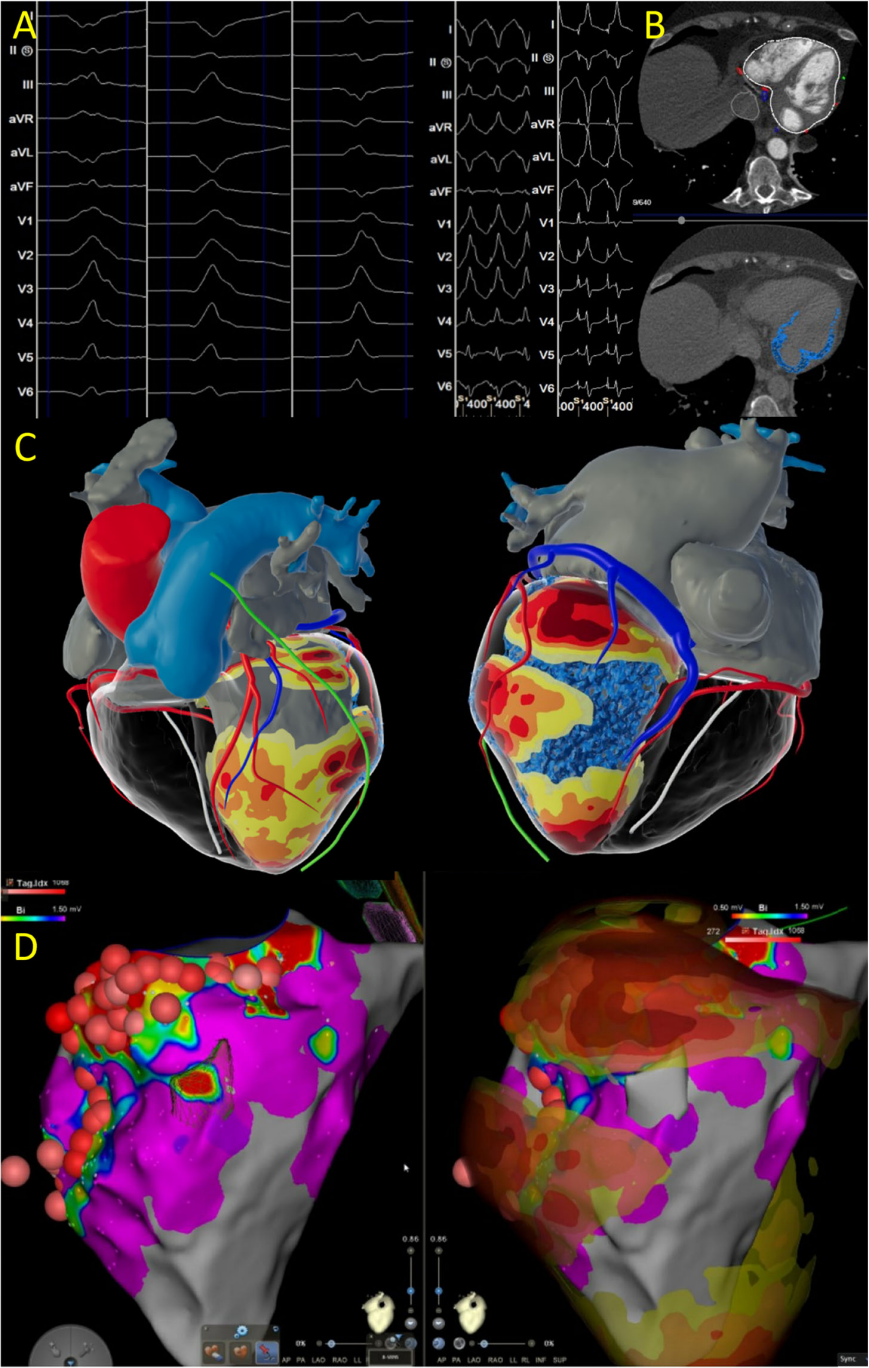
An inHEART image focus for electroanatomical modelling and ablation planning. LGE is a relatively reliable marker of myocardial disease and a potential substrate for scar‐related arrhythmias. Emerging imaging platforms, such as inHEART or ADAS, are increasingly recognized for their pivotal role in facilitating ventricular tachycardia mapping and ablation procedures guided by CMR. (A) PVC and VT morphologies identified in ablation case. (B) CT and MRI imported on inHEART, transverse section. (C) inHEART 3D reconstructed models showing CT wall thickness (thicker yellow to thinner red) and LGE (blue) on the left ventricle. (D) Electroanatomical map showing ablation sites (red spheres) and merged inHEART model on Carto. CT, computed tomography; LGE, late gadolinium enhancement; MRI, magnetic resonance imaging; PVC, premature ventricular contraction; VT, ventricular tachycardia.

In individuals presenting with de novo DCM, or unexplained VAs, CMR imaging can offer insights into prior myocardial inflammation based on the distribution of LGE. Notably, the presence and localization of mid‐layer septal (*mid‐wall strip*) LGE, coupled with low baseline LVEF, serve as robust negative prognostic indicators.^89^, ^90^ While oedema often resolves during follow‐up (up to 84% of cases), LGE typically persists in up to 89% of patients. The extent of LGE reflects a dynamic process in AM, with tissue oedema predominating in the acute phase and gradually diminishing over time, while late‐phase LGE primarily reflects post‐inflammatory replacement fibrosis.^91^ Additionally, CMR feature‐tracking (FT) strain analysis in ACM patients demonstrated layer‐specific diagnostic utility, particularly with mid‐myocardial global circumferential strain (GCS) and subepicardial GLS providing superior results^92^ (*Figure* *7* and *Videos* *S1* *–* *S4*). Moreover, the presence of LGE serves as a reliable indicator of myocardial disease severity and potential substrate for scar‐related arrhythmias, aiding in risk stratification for VAs/SCD.^93^, ^94^, ^95^, ^96^ Novel imaging programmes like inHEART or ADAS (*Figure* *8* and *Figure* *S29*) are increasingly used for guiding ventricular tachycardias (VT) mapping and ablations, as, for example, was demonstrated by Piers *et al*., where contrast‐enhanced CMR can be used to identify critical isthmus site for VT in ischaemic and non‐ischaemic heart disease.^97^ The extent and patterns of LGE also correlate with the risk of VAs/SCD in DCM with specific LGE patterns associated with specific genotypes.^98^, ^99^ CMR thus plays a central role in the genotype–phenotype diagnosis pathway, as described by Augusto *et al*.: the subepicardial ring‐like scar pattern in *DSP/FLNC* can be considered as a possible diagnostic criterion for the definition of arrhythmogenic left ventricular cardiomyopathy (ALVC).^100^ Early CMR incorporation into the initial diagnostic work‐up of NICM enable both diagnostic accuracy and early prognostic stratification. CMR can be equally repeated during follow‐up assessment, providing valuable data on changes in LV volumes and function. Additional details on diagnostic and prognostic CMR findings in DCM are outlined in *Table* *S7*, with CMR indicators associated with DCM phenotypes summarized in *Table* *S8*.

## Prognosis

Despite advancements in DCM treatments, 10‐year survival rates hover around 60%, with frequent HF exacerbations preceding death, underscoring the challenge of individual risk assessment.^41^, ^101^ Notably, emerging evidence underscores the persistence of SCD vulnerability (2–4% annually),^102^ and refractory HF necessitating heart transplant or mechanical circulatory support in select patients.^103^ Approximately 40% of newly diagnosed DCM cases exhibit LVRR within 2 years under optimal medical therapy, portending favourable long‐term outcomes.^104^, ^105^ These evidences challenge (I) the timing of device implantation following at least 3 months of OMT initiation in newly diagnosed DCM patients and (II) the LVEF threshold (≤35%) for primary prevention ICD implantation in symptomatic DCM patients (NYHA class II and III). While LVEF ≤ 35% alone modestly identifies high‐risk SCD patients, other factors like specific disease‐causing variants (e.g., *LMNA*, *PLN*, *DSP*, *FLNC*, *RBM20*) and myocardial scar are crucial in guiding ICD implantation.^5^ Progressive LV dilatation independently predicts cardiovascular death and hospitalization, contrasting with reverse remodelling indicative of enhanced survival.^105^, ^106^ Other imaging parameters useful for risk stratification are described in *Table* *S9*. Additional parameters such as myocardial fibrosis and specific genetic substrate related to an arrhythmic phenotype further refine risk stratification.^55^ For instance, Jones *et al*. demonstrated that advanced LGE characterization in ischaemic heart disease patients, consisting of scar microstructure analysis, quantification of core infarction, and peri‐infarct zone, independently predicts SCD risk beyond conventional predictors.^107^ Genotype–phenotype studies revealed the benefit of primary prevention ICD in patients with specific genotypes, even without severe LV dysfunction (*Table* *S10*). For example, patients carrying malignant *LMNA* variants were firstly recognized as a distinct group with a higher SCD risk; therefore, risk assessment for primary ICD implantation in *LMNA* patients should include calculation of the absolute 5‐year risk of malignant arrhythmias using *LMNA*‐risk Ventricular Tachyarrhythmias Score [https://lmna‐risk‐vta.fr/]^108^; additionally, in the case of the *PLN* p.Arg14del variant, there is a variant‐specific risk‐prediction score [https://plnriskcalculator.shinyapps.io/final_shiny/].^109^ However, the efficacy of primary prevention ICD in non‐ischaemic DCM remains contentious, with limited evidence supporting its total mortality reduction compared to ischaemic heart disease.^110^, ^111^, ^112^, ^113^ The DANISH Study underscores the need to improve primary prevention ICD recommendations in non‐ischaemic DCM.^111^ Comprehensive details on multimodal parameters for ventricular arrhythmias prediction are provided in *Table* *S11*.

### Multimodality imaging tools for risk stratification

LVRR, defined as an increased LVEF > 10% or a LVEF > 50% with a decrease in indexed LVEDD of >10% or indexed LVEDD > 33 mm/m^2^ at 24 months, emerges as a robust indicator in DCM.^105^ Additionally, RV dilatation and dysfunction correlate with worsened functional status and advanced LV failure.^114^ GLS shows promise in predicting mortality in symptomatic DCM patients and detecting early ventricular dysfunction in asymptomatic variant carriers.^115^, ^116^ CMR aids in predicting DCM trajectory, with mid‐wall fibrosis evaluated via LGE being particularly impactful for risk stratification. Also, native T1 and ECV fraction reflect interstitial fibrosis degree, offering potential for improved risk assessment.^117^ FT myocardial strain analysis obtained from cine imaging emerges as another promising tool for improving DCM risk stratification. RV systolic function assessment via RVEF ≤ 45% measured by CMR enhances risk stratification for all‐cause mortality or cardiac transplantation.^59^ Global LV strain parameters showed an additive prognostic value: GLS surpassed standard clinical parameters in prognostication, with GLS and GCS providing additional prognostic insights, even in LVEF ≤ 35%. CMR‐FT, offering fast and reproducible strain assessment, holds promise for routine clinical application.^118^ Parametric mapping sequences, evaluating myocardial T1 and T2 relaxation times, as well as ECV, consistently report higher values in DCM patients compared to controls, reflecting diffuse interstitial fibrosis.^119^, ^120^ Higher native T1 values of myocardium independently predict all‐cause mortality and HF events in DCM patients.^121^ Further details on overall prognostication and outcomes prediction can be found in *Tables* *S12* and *S13*.

## Future perspectives

The introduction of new‐generation CT scanners, such as dual‐source scanners with wide detectors enabling the entire heart volume to be covered in one beat, offers a promising avenue to overcome previous limitations of CCT. Moreover, new‐generation CT scans are characterized by a reduction in gantry rotation time, enhancing temporal resolution for better end‐systolic/end‐diastolic phase identification.^71^ Meanwhile, diffusion tensor CMR emerges as a novel technique enabling in‐depth phenotyping through non‐invasive interrogation of the 3D heart microarchitecture.^122^ It assesses cardiomyocyte and sheetlet orientation through helix angle and absolute angulation of the second eigenvector (E2A), respectively.^59^ While healthy subjects exhibit diastolic wall‐parallel sheetlet alignment transitioning to wall‐perpendicular in systole, DCM patients show altered systolic conformation and reduced mobility, with E2A reduction suggesting sheetlet orientation abnormalities.^122^ Additionally, four‐dimensional (4D) flow CMR is an emerging technology enabling visualization and quantification of intra‐cardiac blood flow, revealing heterogeneous LV haemodynamic forces and diastolic flow routes in DCM patients, potentially serving as subclinical markers of LV dysfunction.^59^, ^123^ Furthermore, the left atrium (LA) has emerged as a significant predictor of cardiovascular events, reflecting LV diastolic filling pressures and contributing to myocardial disease progression. Raafs *et al*. demonstrated the prognostic value of CMR‐derived LA conduit strain in DCM, highlighting its independent association with SCD, HF hospitalization or life‐threatening arrhythmias, incremental to myocardial fibrosis presence and NYHA class >2.^124^ Furthermore, the use of hyperpolarized ^13^C magnetic resonance imaging, with the analysis of oxidized pyruvate levels, could prove valuable in differentiating metabolic changes during heart failure progression, potentially serving as a starting point for identifying DCM.^125^, ^126^, ^127^ Similarly, another frontier in personalized diagnosis and therapy lies in the utilization of extracellular‐based liquid biopsy. When combined with CMR, this approach holds promise for analysing cardiac remodelling, from its earliest stages to more advanced phases.^128^

## Conclusions

DCM can be considered as a wide clinical entity embodying diverse aetiologies and gene–environment interactions, demanding personalized diagnostic, therapeutic, and prognostic strategies. Integrated multimodality imaging approach, with CMR as a robust tool for tissue characterization, alongside genetic testing, enhances DCM phenotype evaluation with the aim of advancing increasingly personalized medicine, even as we await the widespread availability of gene‐specific therapies.^129^

## Conflict of interest

Nothing to disclose.

## Funding

F.R. was supported by the European Union—Next Generation EU, under the National Recovery and Resilience Plan (NRRP), Mission 4 Component 2—M4C2, Investment 1.5—Call No. 3277 of 30.12.2021—The Italian Ministry of University and Research (MUR), Award Number: ECS00000041, project title: ‘Innovation, digitalisation and sustainability for the diffused economy in Central Italy’, Concession Degree No. 1057 of 23.06.2022 adopted by the Italian Ministry of University and Research (MUR). CUP: D73C22000840006.

## Supporting information


**Figure S1:** DCM pathophysiology characteristics, seen on the ECG.
**Figure S2:** Genetics: *TTN* c. 82036C > T (p.Gln27346*). CMR: LGE nonischemic pattern; septum, inferior wall, anterior wall, right ventricular insertion sites; mid myocardial and subepicardial; inflammatory process (myocarditis or sarcoidosis) PET was negative severe left atrial dilation. A: CMR LGE short axis; B: CMR elevated T1; C: CMR elevated T2; D: CMR enlarged LA; E: ECG AF rate controlled and PVCs.
**Figure S2.** Videos ‐ TTN 2nd case found here.
**Figure S3:** Genetics: *LMNA* c.1412G > A (p.Arg471His). DCM hypertrabculation, ICD, AVNRT, aorto mitral tachycardia, PVC, SVT, presyncope. CMR non‐compaction LGE basal infero‐lateral segment mid and subepicardial distribution. A: CMR LGE short axis; B: CMR LGE 2ch; C: CMR LGE 3ch; D: CMR LGE 4ch; E: ECG Sinus RBBB with occasional PVC.
**Figure S4:** Genetics: *FLNC* c.6242dup (p.Ser2082Lysfs*8). aDCM, ALVC, EF35%, CMR There is basal septal thinning with dyskinesis and patchy fibrosis, PET+, AF, PVC 10%. A: CMR LGE short axis; B: CMR LGE 3ch; C: CMR LGE 4ch; D: mild myocardial inflammation involving the basal/mid lateral wall (max SUV 4) and apex (max SUV 4); E: ECG Sinus with occasional PVCs, low voltage in precordial leads.
**Figure S5:** Genetics: *FLNC* c.8110_8111insC (p.Tyr2704Serfs*9). NICM, bystander CAD, PPM, VT, AFL, AF, SVT, LGE medium amount of midmyocardial LGE at basal/mid anterior and anterolateral walls, PET normal. A: CMR LGE short axis; B: CMR LGE 2ch; C: CMR LGE 3ch; D: CMR LGE 4ch; E: ECG electronic atrial and ventricular pacemaker.
**Figure S6:** Genetics: *FLNC* c.1849G > T (p.Glu617*). DCM/ALVC, EF52%, CMR hypokentic LGE subepicardial, mid myocardial distribution involving the basal inferoseptal, basal inferior, mid inferoseptal, mid inferior segments, and RV insertion points.; PVC/VT, ICD, PET normal. A: CMR LGE short axis; B: CMR LGE 2ch; C: CMR LGE 3ch; D: CMR LGE 4ch; E: ECG Sinus with rare PVCs.
**Figure S7:** Genetics: *FLNC* c.4926_4927insACGTCACA (p.Val1643Thrfs*26). NICM, EF30%, LGE in small amount RV insertion, PET+ same CMR, PVC (LVOT), MVP, SVT/AVNRT, VT (RVOT). A: CMR LGE short axis; B: CMR LGE 3ch; C: CMR LGE 4ch; D: small amount of active myocardial inflammation involving the mid inferoseptal wall, max SUV 2.97; E: ECG Sinus with frequent PVCs.
**Figure S8:** Genetics: DMD c.6913‐?_7098+?del (Deletion (Exon 48). NICM, VT, ICD, HFrEF, CMR hypokinetic LGE Extensive enhancement of the lateral wall with both transmural and subepicardial components, with extension into adjacent anterior and inferior segments; PET same as CMR. A: CMR LGE basal short axis; B: CMR LGE mid short axis; C: CMR LGE apical short axis; D: CMR LGE 4ch; E: ECG Sinus with occasional PVCs, intraventricular conduction delay, left axis deviation.
**Figure S9:** Genetics: *ACTC1* c.301G > A (p.Glu101Lys). CMR: There is small amount of midmyocardial late gadolinium enhancement (LGE) at the basal inferolateral wall. Overall, findings are suggestive of an infiltrative process such as Fabry's disease or cardiac sarcoidosis. Prior viral myocarditis can also have similar pattern in the correct clinical context. Danon disease is possible although less likely given LGE pattern. A: CMR LGE short axis; B: CMR LGE 3ch; C: CMR LGE 4ch; D: CT‐PET uptake along the basal to mid anteroseptum at the RV insertion point (max SUV 5.3), basal to mid inferior septum; E: Sinus tachycardia, Incomplete right bundle branch block, Left anterior fascicular block, Left ventricular hypertrophy and ST‐T change.
**Figure S10:** Genetics: *DSP* c.7075del(p.Ile2359Serfs*10). PVC (RVOT) ablated, early NICM (arrythmia), AF; PET normal; CMR: no LGE. A: CMR LGE short axis; B: CMR LGE 2ch; C: CMR LGE 3ch; D: CMR LGE 4ch; E: Sinus rhythm with PVCs, there is poor R‐wave progression.
**Figure S11:** Genetics: *MYH7* c.3865C > T (p.Arg1289Trp). Dilated cardiomyopathy (LVEDVi 112 mL/m^2^; LVEDV 219 mL) with basal septum midwall LGE. A: CMR LGE 4 chamber view; B: CMR LGE short axis multislice view; C: ECG showing sinus rhythm and reduced R wave progression in the precordial leads.
**Figure S12:** Genetics: *SCN5A* c.5350G > A (p.Glu1784Lys). LQTS 3, PVC/VF/PMVT/storm, ICD, CMR: delayed enhancement sequences are suboptimal due to a susceptibility artifact over the mid and distal anterior all secondary to pacemaker however no obvious evidence of an infiltrative cardiomyopathy or ischemic damage in the segments visualized. No obvious evidence of myocardial edema in the visualized segments of the myocardium. A: CMR LGE short axis; B: CMR LGE 2ch; C: CMR LGE 3ch; D: CMR LGE 4ch; E: ECG Electronic atrial pacemaker PVC, Moderate T‐wave abnormality, LQTS 3.
**Figure S13:** Genetics: *BAG3* c.499G > A (p.Glu167Arg). A: CMR LGE short axis; B: CMR LGE 2ch; C: CMR LGE 3ch; D: CMR LGE 4ch; E: ECG.
**Figure S14:** Genetics: *TTNT2* c.832C > T (p.Arg278Cys). A: CMR LGE short axis; B: CMR LGE 2ch; C: CMR LGE 3ch; D: CMR LGE 4ch; E: ECG.
**Figure S15:** Genetics: *DES* c.1048C > T (p.Arg350Trp). A: CMR LGE short axis; B: CMR LGE 2ch; C: CMR LGE 3ch; D: CMR LGE 4ch; E: ECG.
**Figure S16:** Genetics: *PKP2* c.2312_2313del (p.Leu771Profs*2). A: CMR LGE short axis; B: CMR LGE 2ch; C: CMR LGE 3ch; D: CMR LGE 4ch; E: ECG.
**Figure S17:** Genetics: *DSC2* c.2039G > A (p.Arg680His). A: CMR LGE short axis; B: CMR LGE 2ch; C: CMR LGE 3ch; D: CMR LGE 4ch; E: ECG.
**Figure S18:** Genetics: *DSG2* c.146G > A (p.Arg49His). A: CMR LGE short axis; B: CMR LGE 2ch; C: CMR LGE 3ch; D: CMR LGE 4ch; E: ECG.
**Figure S19:** Genetics: *TMEM43* c.98C > T (p.Ser33Leu). A: CMR LGE short axis; B: CMR LGE 2ch; C: CMR LGE 3ch; D: CMR LGE 4ch; E: ECG.
**Figure S20:** Genetics: *JUP* c.509C > T (p.Ser170Leu). A: CMR LGE short axis; B: CMR LGE 2ch; C: CMR LGE 3ch; D: CMR LGE 4ch; E: ECG.
**Figure S21:** Genetics: *SCN5A* c.665G > A (p.R222Q: CGA > CAA). 24‐year‐old man, black, referred to CMR for excessive trabeculations and dilated phenotype by echocardiogram. A: CMR LGE short axis showing mild midwall circumferential LGE of basal segments; B: CMR LGE 2ch; C: CMR LGE 3ch; D: CMR LGE 4ch; E: ECG.
**Figure S22.** RBM20 Mutation in patient with COVID‐related myocarditis. The evaluation of the inferior wall is limited due to artifact from the subcutaneous pacemaker however no obvious delayed enhancement noted in the visualized segments of the myocardium. No myocardial edema by T2‐weighted imaging noted in the visualized segments of the cardia. Genetics: *RBM20* c.2737G > A (p.Glu913Lys). A: CMR LGE short axis; B: CMR LGE 2ch; C: CMR LGE 3ch; D: CMR LGE 4ch; E: ECG Ventricular tachycardia, LBBB. CMR: Cardiac Magnetic Resonance; LBBB: Left Bundle Branch Block.
**Figure S23.** ALVC in patient with TTN mutation. ALVC in a patient with severely reduced LV ejection fraction (LVEF: 15%). ECG findings: TWI and RVOT ventricular tachycardias/premature ventricular contractions. CMR: excessive LV trabeculation, midmyocardial LGE at basal/mid septum. ICD implantation. Genetics: *TTN* c. 98994del (p.Lys32998Asnfs*63). A: CMR LGE short axis; B: CMR LGE 2ch; C: CMR LGE 3ch; D: CMR LGE 4ch; E: ECG Sinus with T inversions. ALVC: Arrhythmogenic Left Ventricular Cardiomyopathy; TWI: T Wave Inversion; RVOT: Right Ventricular Outflow Tract.
**Figure S24.** DCM in patient with haemochromatosis. A: CMR survey scan; B: CMR T1 mapping; C: CMR ECV; D: CMR LGE short axis. DCM: Dilated Cardiomyopathy; CMR: Cardiac Magnetic Resonance; ECV: Extracellular Volume; LGE: Late Gadolinium Enhancement.
**Figure S25.** ALVC in patient with FLNC mutation. A: CMR 4 chamber cine view; B: CMR LGE short axis view. ALVC: Arrhythmogenic Left Ventricular Cardiomyopathy; CMR: Cardiac Magnetic Resonance; FLNC: Filamin C gene; LGE: Late Gadolinium Enhancement.
**Figure S26.** DCM in patient with Duchenne Muscular Dystrophy. A: CMR 4 chamber cine view; B: CMR LGE 4 chamber view; C: CMR LGE short axis view. DCM: Dilated Cardiomyopathy; CMR: Cardiac Magnetic Resonance; LGE: Late Gadolinium Enhancement.
**Figure S27.** DCM in patient with hereditary ATTR characterized by cardiac and neurological involvement. Genetics: *TTR* missense variant c.160G (p.Arg54Gly); A: CMR 2 chamber LGE view; B: CMR LGE short axis view; C: CMR LGE 4 chamber view. ATTR: Transthyretin‐related Hereditary Amyloidosis; DCM: Dilated Cardiomyopathy; CMR: Cardiac Magnetic Resonance; LGE: Late Gadolinium Enhancement; TTR: Transthyretin.
**Figure S28:** Cardiac Genetic Service Specialists involved in DCM management. Genetic testing should be offered to all first‐degree relatives if a pathogenic or likely pathogenic variant has been detected in the family because, even in genotype–phenotype‐negative members, because a non‐monogenic DCM variant could be present. DCM: Dilated Cardiomyopathy.
**Figure S29.** ADAS case report. A: VT morphology RBRI, with positive concordance precordial leads, B: CMR T1 LGE images showing areas of subendocardial LGE *yellow arrow), and significant device artefact; C: ADAS 3D reconstructed model showing left ventricle wall thickness (thicker blue to thinner red), D: Electroanatomical map showing Bipolar voltage maps and regions of ablation (red spheres) correlating with transition region from thick to thin on ADAS. VT: Ventricular Tachycardia; RBRI: Right Bundle Branch Block – Right Inferior; CMR: Cardiac Magnetic Resonance; LGE: Late Gadolinium Enhancement.
**Table S1.** Main clinical and laboratory findings in DCM phenotypes.
**Table S2.** Diagnostic criteria for relatives or familial DCM [adapted from EACVI 2019 Consensus document].
**Table S3.** Main definitions of myocarditis [adapted from Ammirati et al.].
**Table S4.** Clinical and PVC features to identify PVC‐Cardiomyopathy [taken from Huizar et al., JACC 2019].
**Table S5.** Main Echocardiography parameters in the diagnosis and follow up of DCM [adapted from Mitropoulou et al.].
**Table S6.** Nuclear Imaging in the diagnosis of DCM etiology: main tracers and future applications.
**Table S7.** Main diagnostic and prognostic CMR findings in DCM [adapted from Merlo et al.]. In *Italics*, the optional techniques.
**Table S8.** CMR findings associated to DCM phenotypes.
**Table S9.** Main prognostic markers in DCM [adapted from EACVI 2019 Consensus document].
**Table S10.** ICD recommendations based on genetic variants [adapted from Orphanou et al.].
**Table S11.** Multimodality imaging and specific predictors for ventricular arrhythmias in patients with DCM [adapted from EACVI 2019 Consensus document].
**Table S12.** Overall prognostication of adverse events in DCM via CMR [adapted from Mitropoulou et al.].
**Table S13.** Outcome prediction via CMR [adapted from Mitropoulou et al.].

## References

[1] Gulati A , Ismail TF , Jabbour A , Alpendurada F , Guha K , Ismail NA , *et al*. The prevalence and prognostic significance of right ventricular systolic dysfunction in nonischemic dilated cardiomyopathy. Circulation 2013;128:1623‐1633. doi:10.1161/CIRCULATIONAHA.113.002518 23965488

[2] Pinto YM , Elliott PM , Arbustini E , Adler Y , Anastasakis A , Böhm M , *et al*. Proposal for a revised definition of dilated cardiomyopathy, hypokinetic non‐dilated cardiomyopathy, and its implications for clinical practice: a position statement of the ESC working group on myocardial and pericardial diseases. Eur Heart J 2016;37:1850‐1858. doi:10.1093/eurheartj/ehv727 26792875

[3] Caforio ALP , Adler Y , Agostini C , Allanore Y , Anastasakis A , Arad M , *et al*. Diagnosis and management of myocardial involvement in systemic immune‐mediated diseases: a position statement of the European Society of Cardiology Working Group on Myocardial and Pericardial Disease. Eur Heart J 2017;38:2649‐2662. doi:10.1093/eurheartj/ehx321 28655210

[4] McKenna WJ , Judge DP . Epidemiology of the inherited cardiomyopathies. Nat Rev Cardiol 2021;18:22‐36. doi:10.1038/s41569-020-0428-2 32895535

[5] Arbelo E , Protonotarios A , Gimeno JR , Arbustini E , Barriales‐Villa R , Basso C , *et al*. 2023 ESC guidelines for the management of cardiomyopathies. Eur Heart J 2023;44:3503‐3626. doi:10.1093/eurheartj/ehad194 37622657

[6] Lipshultz SE , Law YM , Asante‐Korang A , Austin ED , Dipchand AI , Everitt MD , *et al*. Cardiomyopathy in children: classification and diagnosis: a scientific statement from the American Heart Association. Circulation 2019;140:e9‐e68. doi:10.1161/CIR.0000000000000682 31132865

[7] Rakar S , Sinagra G , Di Lenarda A , Poletti A , Bussani R , Silvestri F , *et al*. Epidemiology of dilated cardiomyopathy. A prospective post‐mortem study of 5252 necropsies. Eur Heart J 1997;18:117‐123. doi:10.1093/oxfordjournals.eurheartj.a015092 9049523

[8] Hershberger RE , Hedges DJ , Morales A . Dilated cardiomyopathy: the complexity of a diverse genetic architecture. Nat Rev Cardiol 2013;10:531‐547. doi:10.1038/nrcardio.2013.105 23900355

[9] Ganesh SK , Arnett DK , Assimes TL , Basson CT , Chakravarti A , Ellinor PT , *et al*. Genetics and genomics for the prevention and treatment of cardiovascular disease: update a scientific statement from the american heart association. Circulation 2013;128:2813‐2851. doi:10.1161/01.cir.0000437913.98912.1d 24297835

[10] Michels VV , Moll PP , Miller FA , Tajik AJ , Chu JS , Driscoll DJ , *et al*. The frequency of familial dilated cardiomyopathy in a series of patients with idiopathic dilated cardiomyopathy. N Engl J Med 1992;326:77‐82. doi:10.1056/NEJM199201093260201 1727235

[11] Jordan E , Peterson L , Ai T , Asatryan B , Bronicki L , Brown E , *et al*. Evidence‐based assessment of genes in dilated cardiomyopathy. Circulation 2021;144:7‐19. 33947203 10.1161/CIRCULATIONAHA.120.053033 PMC8247549

[12] Elliott P , Andersson B , Arbustini E , Bilinska Z , Cecchi F , Charron P , *et al*. Classification of the cardiomyopathies: a position statement from the European Society of Cardiology Working Group on Myocardial and Pericardial Diseases. Eur Heart J 2008;29:270‐276. doi:10.1093/eurheartj/ehm342 17916581

[13] Arbustini E , Morbini P , Pilotto A , Gavazzi A , Tavazzi L . Genetics of idiopathic dilated cardiomyopathy. Herz 2000;25:156‐160. doi:10.1007/s000590050001 10904833

[14] Grünig E , Tasman JA , Kücherer H , Franz W , Kübler W , Katus HA . Frequency and phenotypes of familial dilated cardiomyopathy. J Am Coll Cardiol 1998;31:186‐194. doi:10.1016/s0735-1097(97)00434-8 9426039

[15] Sorella A , Galanti K , Iezzi L , Gallina S , Mohammed SF , Sekhri N , *et al*. Diagnosis and management of dilated cardiomyopathy: a systematic review of clinical practice guidelines and recommendations. Eur Heart J Qual Care Clin Outcomes 2024;11:206‐222. doi:10.1093/ehjqcco/qcae109 PMC11879293 39674807

[16] Wilde AAM , Semsarian C , Márquez MF , Shamloo AS , Ackerman MJ , Ashley EA , *et al*. European Heart Rhythm Association (EHRA)/Heart Rhythm Society (HRS)/Asia Pacific Heart Rhythm Society (APHRS)/Latin American Heart Rhythm Society (LAHRS) expert consensus statement on the state of genetic testing for cardiac diseases. Europace 2022;24:1307‐1367. doi:10.1093/europace/euac030 35373836 PMC9435643

[17] Chahal CAA , Landstrom AP . Predicting the development of dilated cardiomyopathy in kindred with genetic risk. J Am Coll Cardiol 2024 Apr;83:1652‐1655. doi:10.1016/j.jacc.2024.03.381 38658104

[18] Kurzlechner LM , Kishnani S , Chowdhury S , Atkins SL , Moya‐Mendez ME , Parker LE , *et al*. DiscoVari: a web‐based precision medicine tool for predicting variant pathogenicity in cardiomyopathy‐ and channelopathy‐associated genes. Circ Genom Precis Med 2023 Aug;16:317‐327. doi:10.1161/CIRCGEN.122.003911 37409478 PMC10527712

[19] Houge G , Laner A , Cirak S , de Leeuw N , Scheffer H , den Dunnen JT . Stepwise ABC system for classification of any type of genetic variant. Eur J Hum Genet 2022;30:150‐159. doi:10.1038/s41431-021-00903-z 33981013 PMC8821602

[20] Houge G , Bratland E , Aukrust I , Tveten K , Žukauskaitė G , Sansovic I , *et al*. Comparison of the ABC and ACMG systems for variant classification. Eur J Hum Genet 2024;32:858‐863. doi:10.1038/s41431-024-01617-8 38778080 PMC11219933

[21] Hershberger RE , Givertz MM , Ho CY , Judge DP , Kantor PF , McBride KL , *et al*. Genetic evaluation of cardiomyopathy—a Heart Failure Society of America practice guideline. J Card Fail 2018;24:281‐302. doi:10.1016/j.cardfail.2018.03.004 29567486 PMC9903357

[22] Musunuru K , Hershberger RE , Day SM , Klinedinst NJ , Landstrom AP , Parikh VN , *et al*. Genetic testing for inherited cardiovascular diseases: a scientific statement from the American Heart Association. Circ Genom Precis Med 2020;13:e000067. doi:10.1161/HCG.0000000000000067 32698598

[23] Parker LE , Landstrom AP . The clinical utility of pediatric cardiomyopathy genetic testing: from diagnosis to a precision medicine‐based approach to care. Prog Pediatr Cardiol 2021;62:101413. doi:10.1016/j.ppedcard.2021.101413 34776723 PMC8579834

[24] Mestroni L , Rocco C , Gregori D , Sinagra G , Di Lenarda A , Miocic S , *et al*. Familial dilated cardiomyopathy. J Am Coll Cardiol 1999;34:181‐190. 10400009 10.1016/s0735-1097(99)00172-2

[25] Ricci F , Banihashemi B , Pirouzifard M , Sundquist J , Sundquist K , Sutton R , *et al*. Familial risk of dilated and hypertrophic cardiomyopathy: a national family study in Sweden. ESC Heart Fail 2023;10:121‐132. doi:10.1002/ehf2.14171 36169166 PMC9871695

[26] Charron P , Arad M , Arbustini E , Basso C , Bilinska Z , Elliott P , *et al*. Genetic counselling and testing in cardiomyopathies: a position statement of the European Society of Cardiology Working Group on Myocardial and Pericardial Diseases. Eur Heart J 2010;31:2715‐2726. doi:10.1093/eurheartj/ehq271 20823110

[27] Patel V , Asatryan B , Siripanthong B , Munroe PB , Tiku‐Owens A , Lopes LR , *et al*. State of the art review on genetics and precision medicine in arrhythmogenic cardiomyopathy. Int J Mol Sci 2020;21:6615. doi:10.3390/ijms21186615 32927679 PMC7554944

[28] Dungu JN , Langley SG , Hardy‐Wallace A , Li B , Barbagallo RM , Field D , *et al*. Dilated cardiomyopathy: the role of genetics, highlighted in a family with filamin C (FLNC) variant. Heart 2022;108:676‐682. doi:10.1136/heartjnl-2021-319682 34417207

[29] Rapezzi C , Arbustini E , Caforio ALP , Charron P , Gimeno‐Blanes J , Heliö T , *et al*. Diagnostic work‐up in cardiomyopathies: bridging the gap between clinical phenotypes and final diagnosis. A position statement from the ESC Working Group on Myocardial and Pericardial Diseases. Eur Heart J 2013;34:1448‐1458. doi:10.1093/eurheartj/ehs397 23211230

[30] Momiyama Y , Mitamura H , Kimura M . ECG characteristics of dilated cardiomyopathy. J Electrocardiol 1994;27:323‐328. doi:10.1016/s0022-0736(05)80270-5 7815010

[31] Merlo M , Zaffalon D , Stolfo D , Altinier A , Barbati G , Zecchin M , *et al*. ECG in dilated cardiomyopathy: specific findings and long‐term prognostic significance. J Cardiovasc Med 2019;20:450‐458. doi:10.2459/JCM.0000000000000804 30985353

[32] Letnes JM , Nes BM , Langlo KAR , Aksetøy ILA , Lundgren KM , Skovereng K , *et al*. Indexing cardiac volumes for peak oxygen uptake to improve differentiation of physiological and pathological remodeling: from elite athletes to heart failure patients. Eur Heart J Cardiovasc Imaging 2023;24:721‐729. doi:10.1093/ehjci/jead034 37073553 PMC10229299

[33] Escobar‐Lopez L , Ochoa JP , Royuela A , Verdonschot JAJ , Dal Ferro M , Espinosa MA , *et al*. Clinical risk score to predict pathogenic genotypes in patients with dilated cardiomyopathy. J Am Coll Cardiol 2022;80:1115‐1126. doi:10.1016/j.jacc.2022.06.040 36109106 PMC10804447

[34] Merlo M , Cannatà A , Gobbo M , Stolfo D , Elliott PM , Sinagra G . Evolving concepts in dilated cardiomyopathy. Eur J Heart Fail 2018;20:228‐239. doi:10.1002/ejhf.1103 29271570

[35] Bawaskar P , Thomas N , Ismail K , Guo Y , Chhikara S , Athwal PSS , *et al*. Nonischemic or dual cardiomyopathy in patients with coronary artery disease. Circulation 2024;149:807‐821. doi:10.1161/CIRCULATIONAHA.123.067032 37929565 PMC10951941

[36] Yoneda ZT , Anderson KC , Quintana JA , O'Neill MJ , Sims RA , Glazer AM , *et al*. Early‐onset atrial fibrillation and the prevalence of rare variants in cardiomyopathy and arrhythmia genes. JAMA Cardiol 2021;6:1371. doi:10.1001/jamacardio.2021.3370 34495297 PMC8427496

[37] Ware JS , Li J , Mazaika E , Yasso CM , DeSouza T , Cappola TP , *et al*. Shared genetic predisposition in peripartum and dilated cardiomyopathies. N Engl J Med 2016;374:2601‐2602. doi:10.1056/NEJMc1602671 PMC4797319 26735901

[38] Ware JS , Amor‐Salamanca A , Tayal U , Govind R , Serrano I , Salazar‐Mendiguchía J , *et al*. Genetic etiology for alcohol‐induced cardiac toxicity. J Am Coll Cardiol 2018;71:2293‐2302. 29773157 10.1016/j.jacc.2018.03.462 PMC5957753

[39] Huizar JF , Ellenbogen KA , Tan AY , Kaszala K . Arrhythmia‐induced cardiomyopathy: JACC state‐of‐the‐art review. J Am Coll Cardiol 2019;73:2328‐2344. doi:10.1016/j.jacc.2019.02.045 31072578 PMC6538508

[40] Fatkin D , Yeoh T , Hayward CS , Benson V , Sheu A , Richmond Z , *et al*. Evaluation of left ventricular enlargement as a marker of early disease in familial dilated cardiomyopathy. Circ Cardiovasc Genet 2011;4:342‐348. doi:10.1161/CIRCGENETICS.110.958918 21636824

[41] Japp AG , Gulati A , Cook SA , Cowie MR , Prasad SK . The diagnosis and evaluation of dilated cardiomyopathy. J Am Coll Cardiol 2016;67:2996‐3010. doi:10.1016/j.jacc.2016.03.590 27339497

[42] Donal E , Delgado V , Bucciarelli‐Ducci C , Galli E , Haugaa KH , Charron P , *et al*. Multimodality imaging in the diagnosis, risk stratification, and management of patients with dilated cardiomyopathies: an expert consensus document from the European Association of Cardiovascular Imaging. Eur Heart J Cardiovasc Imaging 2019;20:1075‐1093. doi:10.1093/ehjci/jez178 31504368

[43] Drazner MH , Bozkurt B , Cooper LT , Aggarwal NR , Basso C , Bhave NM , *et al*. 2024 ACC expert consensus decision pathway on strategies and criteria for the diagnosis and management of myocarditis. J Am Coll Cardiol 2025;85:391‐431. doi:10.1016/j.jacc.2024.10.080 39665703

[44] Asatryan B , Asimaki A , Landstrom AP , Khanji MY , Odening KE , Cooper LT , *et al*. Inflammation and immune response in Arrhythmogenic cardiomyopathy: state‐of‐the‐art review. Circulation 2021;144:1646‐1655. doi:10.1161/CIRCULATIONAHA.121.055890 34780255 PMC9034711

[45] Towbin JA , Lowe AM , Colan SD , Sleeper LA , Orav EJ , Clunie S , *et al*. Incidence, causes, and outcomes of dilated cardiomyopathy in children. JAMA 2006;296:1867‐1876. doi:10.1001/jama.296.15.1867 17047217

[46] Ammirati E , Frigerio M , Adler ED , Basso C , Birnie DH , Brambatti M , *et al*. Management of acute myocarditis and chronic inflammatory cardiomyopathy: an expert consensus document. Circ Heart Fail 2020;13:e007405. doi:10.1161/CIRCHEARTFAILURE.120.007405 33176455 PMC7673642

[47] Ammirati E , Veronese G , Cipriani M , Moroni F , Garascia A , Brambatti M , *et al*. Acute and fulminant myocarditis: a pragmatic clinical approach to diagnosis and treatment. Curr Cardiol Rep 2018;20:114. doi:10.1007/s11886-018-1054-z 30259175

[48] Basso C , Thiene G , Corrado D , Angelini A , Nava A , Valente M . Arrhythmogenic right ventricular cardiomyopathy: dysplasia, dystrophy, or myocarditis? Circulation 1996;94:983‐991. doi:10.1161/01.CIR.94.5.983 8790036

[49] Peretto G , De Luca G , Villatore A , Di Resta C , Sala S , Palmisano A , *et al*. Multimodal detection and targeting of biopsy‐proven myocardial inflammation in genetic cardiomyopathies: a pilot report. JACC Basic Transl Sci 2023;8:755‐765. doi:10.1016/j.jacbts.2023.02.018 37547072 PMC10401291

[50] Weber BN , Paik JJ , Aghayev A , Klein AL , Mavrogeni SI , Yu PB , *et al*. Novel imaging approaches to cardiac manifestations of systemic inflammatory diseases. J Am Coll Cardiol 2023;82:2128‐2151. doi:10.1016/j.jacc.2023.09.819 37993205 PMC11238243

[51] Shoureshi P , Tan AY , Koneru J , Ellenbogen KA , Kaszala K , Huizar JF . Arrhythmia‐induced cardiomyopathy. J Am Coll Cardiol 2024;83:2214‐2232. doi:10.1016/j.jacc.2024.03.416 38811098

[52] Bozkurt B , Colvin M , Cook J , Cooper LT , Deswal A , Fonarow GC , *et al*. Current diagnostic and treatment strategies for specific dilated cardiomyopathies: a scientific statement from the American Heart Association. Circulation 2016;134:e579‐e646. doi:10.1161/CIR.0000000000000455 27832612

[53] Penela D , Fernández‐Armenta J , Aguinaga L , Tercedor L , Ordoñez A , Bisbal F , *et al*. Clinical recognition of pure premature ventricular complex‐induced cardiomyopathy at presentation. Heart Rhythm 2017;14:1864‐1870. doi:10.1016/j.hrthm.2017.07.025 28756100

[54] Thomas DE , Wheeler R , Yousef ZR , Masani ND . The role of echocardiography in guiding management in dilated cardiomyopathy. Eur J Echocardiogr 2009;10:iii15‐iii21. doi:10.1093/ejechocard/jep158 19889654

[55] Orphanou N , Papatheodorou E , Anastasakis A . Dilated cardiomyopathy in the era of precision medicine: latest concepts and developments. Heart Fail Rev 2022;27:1173‐1191. doi:10.1007/s10741-021-10139-0 34263412 PMC8279384

[56] Lang RM , Badano LP , Victor MA , Afilalo J , Armstrong A , Ernande L , *et al*. Recommendations for cardiac chamber quantification by echocardiography in adults: an update from the American Society of Echocardiography and the European Association of Cardiovascular Imaging. J Am Soc Echocardiogr 2015;28:1‐39. doi:10.1016/j.echo.2014.10.003 25559473

[57] Clark J , Ionescu A , Chahal CAA , Bhattacharyya S , Lloyd G , Galanti K , *et al*. Interchangeability in left ventricular ejection fraction measured by echocardiography and cardiovascular magnetic resonance: not a perfect match in the real world. Curr Probl Cardiol 2023;48:101721. doi:10.1016/j.cpcardiol.2023.101721 37001574

[58] Donal E , De Place C , Kervio G , Bauer F , Gervais R , Leclercq C , *et al*. Mitral regurgitation in dilated cardiomyopathy: value of both regional left ventricular contractility and dyssynchrony. Eur J Echocardiogr 2009;10:133‐138. doi:10.1093/ejechocard/jen188 18586669

[59] Mitropoulou P , Georgiopoulos G , Figliozzi S , Klettas D , Nicoli F , Masci PG . Multi‐modality imaging in dilated cardiomyopathy: with a focus on the role of cardiac magnetic resonance. Front Cardiovasc Med 2020;7:97. doi:10.3389/fcvm.2020.00097 32714942 PMC7343712

[60] Caballero L , Kou S , Dulgheru R , Gonjilashvili N , Athanassopoulos GD , Barone D , *et al*. Echocardiographic reference ranges for normal cardiac Doppler data: results from the NORRE study. Eur Heart J Cardiovasc Imaging 2015;16:1031‐1041. doi:10.1093/ehjci/jev083 25896355

[61] Sugimoto T , Dulgheru R , Bernard A , Ilardi F , Contu L , Addetia K , *et al*. Echocardiographic reference ranges for normal left ventricular 2D strain: results from the EACVI NORRE study. Eur Heart J Cardiovasc Imaging 2017;18:833‐840. doi:10.1093/ehjci/jex140 28637227

[62] Farsalinos KE , Daraban AM , Ünlü S , Thomas JD , Badano LP , Voigt JU . Head‐to‐head comparison of global longitudinal strain measurements among nine different vendors: the EACVI/ASE inter‐vendor comparison study. J Am Soc Echocardiogr 2015;28:1171‐1181. doi:10.1016/j.echo.2015.06.011 26209911

[63] Gimelli A , Liga R , Avogliero F , Coceani M , Marzullo P . Relationships between left ventricular sympathetic innervation and diastolic dysfunction: the role of myocardial innervation/perfusion mismatch. J Nucl Cardiol 2018;25:1101‐1109. doi:10.1007/s12350-016-0753-3 28028761

[64] Majmudar MD , Murthy VL , Shah RV , Kolli S , Mousavi N , Foster CR , *et al*. Quantification of coronary flow reserve in patients with ischaemic and non‐ischaemic cardiomyopathy and its association with clinical outcomes. Eur Heart J Cardiovasc Imaging 2015;16:900‐909. doi:10.1093/ehjci/jev012 25719181 PMC4592320

[65] Gunaratnam K , Wong LH , Nasis A , Ellims A , Nandurkar D , Soo G , *et al*. Review of cardiomyopathy imaging. Eur J Radiol 2013;82:1763‐1775. doi:10.1016/j.ejrad.2013.05.041 23827801

[66] Naik MM , Diamond GA , Pai T , Soffer A , Siegel RJ . Correspondence of left ventricular ejection fraction determinations from two‐dimensional echocardiography, radionuclide angiography and contrast cineangiography. J Am Coll Cardiol 1995;25:937‐942. doi:10.1016/0735-1097(94)00506-L 7884101

[67] Perel‐Winkler A , Bokhari S , Perez‐Recio T , Zartoshti A , Askanase A , Geraldino‐Pardilla L . Myocarditis in systemic lupus erythematosus diagnosed by 18 F‐fluorodeoxyglucose positron emission tomography. Lupus Sci Med 2018;5:e000265. doi:10.1136/lupus-2018-000265 30094040 PMC6069920

[68] Birnie DH , Nery PB , Ha AC , Beanlands RSB . Cardiac sarcoidosis. J Am Coll Cardiol 2016;68:411‐421. doi:10.1016/j.jacc.2016.03.605 27443438

[69] Chareonthaitawee P , Beanlands RS , Chen W , Dorbala S , Miller EJ , Murthy VL , *et al*. Joint SNMMI–ASNC expert consensus document on the role of 18 F‐FDG PET/CT in cardiac sarcoid detection and therapy monitoring. J Nucl Med 2017;58:1341‐1353. doi:10.2967/jnumed.117.196287 28765228 PMC6944184

[70] Alfawara MS , Ahmed AI , Saad JM , Han Y , Alahdab F , Al Rifai M , *et al*. The utility of beta‐hydroxybutyrate in detecting myocardial glucose uptake suppression in patients undergoing inflammatory [18F]‐FDG PET studies. Eur J Nucl Med Mol Imaging 2023;50:1103‐1110. doi:10.1007/s00259-022-06062-7 36474124

[71] Conte E , Mushtaq S , Muscogiuri G , Formenti A , Annoni A , Mancini E , *et al*. The potential role of cardiac CT in the evaluation of patients with known or suspected cardiomyopathy: from traditional indications to novel clinical applications. Front Cardiovasc Med 2021;8:709124. doi:10.3389/fcvm.2021.709124 34595219 PMC8476802

[72] Paras ML , Khurshid S , Foldyna B , Huang AL , Hohmann EL , Cooper LT , *et al*. Case 13‐2022: a 56‐year‐old man with Myalgias, fever, and bradycardia. N Engl J Med 2022;386:1647‐1657. doi:10.1056/NEJMcpc2201233 35476654

[73] Koo BK , Erglis A , Doh JH , Daniels DV , Jegere S , Kim HS , *et al*. Diagnosis of ischemia‐causing coronary stenoses by noninvasive fractional flow reserve computed from coronary computed tomographic angiograms: results from the prospective multicenter DISCOVER‐FLOW (diagnosis of ischemia‐causing stenoses obtained via noninvasive fractional flow reserve) study. J Am Coll Cardiol 2011;58:1989‐1997. doi:10.1016/j.jacc.2011.06.066 22032711

[74] George RT , Mehra VC , Chen MY , Kitagawa K , Arbab‐Zadeh A , Miller JM , *et al*. Myocardial CT perfusion imaging and SPECT for the diagnosis of coronary artery disease: a head‐to‐head comparison from the CORE320 multicenter diagnostic performance study. Radiology 2014;272:407‐416. doi:10.1148/radiol.14140806 24865312 PMC4263655

[75] Neumann FJ , Sechtem U , Banning AP , Bonaros N , Bueno H , Bugiardini R , *et al*. 2019 ESC guidelines for the diagnosis and management of chronic coronary syndromes. Eur Heart J 2020;41:407‐477. doi:10.1093/eurheartj/ehz425 31504439

[76] Si‐Mohamed SA , Boccalini S , Lacombe H , Diaw A , Varasteh M , Rodesch PA , *et al*. Coronary CT angiography with photon‐counting CT: first‐in‐human results. Radiology 2022;303:303‐313. doi:10.1148/radiol.211780 35166583

[77] Sandfort V , Persson M , Pourmorteza A , Noël PB , Fleischmann D , Willemink MJ . Spectral photon‐counting CT in cardiovascular imaging. J Cardiovasc Comput Tomogr 2021;15:218‐225. doi:10.1016/j.jcct.2020.12.005 33358186

[78] Boulmier D , Audinet C , Heautot JF , Larralde A , Veillard D , Hamonic S , *et al*. Clinical contributions of 64‐slice computed tomography in the evaluation of cardiomyopathy of unknown origin. Arch Cardiovasc Dis 2009;102:685‐696. doi:10.1016/j.acvd.2009.06.004 19913770

[79] Merlo M , Gagno G , Baritussio A , Bauce B , Biagini E , Canepa M , *et al*. Clinical application of CMR in cardiomyopathies: evolving concepts and techniques: a position paper of myocardial and pericardial diseases and cardiac magnetic resonance working groups of Italian society of cardiology. Heart Fail Rev 2023;28:77‐95. doi:10.1007/s10741-022-10235-9 35536402 PMC9902331

[80] Mavrogeni S , Dimitroulas T , Kitas GD . Multimodality imaging and the emerging role of cardiac magnetic resonance in autoimmune myocarditis. Autoimmun Rev 2012;12:305‐312. doi:10.1016/j.autrev.2012.05.005 22617620

[81] Woolen SA , Shankar PR , Gagnier JJ , MacEachern MP , Singer L , Davenport MS . Risk of nephrogenic systemic fibrosis in patients with stage 4 or 5 chronic kidney disease receiving a group II gadolinium‐based contrast agent: a systematic review and meta‐analysis. JAMA Intern Med 2020;180:223‐230. doi:10.1001/jamainternmed.2019.5284 31816007 PMC6902198

[82] Parwani P , Chen T , Allen B , Kallianos K , Ng MY , Kozor R , *et al*. Challenges and opportunities for early career medical professionals in cardiovascular magnetic resonance (CMR) imaging: a white paper from the Society for Cardiovascular Magnetic Resonance. J Cardiovasc Magn Reson 2023;25:65. doi:10.1186/s12968-023-00968-3 37968709 PMC10652595

[83] López‐Fernández T , Thavendiranathan P . Emerging cardiac imaging modalities for the early detection of cardiotoxicity due to anticancer therapies. Rev Esp Cardiol (Engl Ed) 2017;70:487‐495. doi:10.1016/j.rec.2017.01.004 28189542

[84] Di Marco A , Anguera I , Schmitt M , Klem I , Neilan T , White JA , *et al*. Late gadolinium enhancement and the risk for ventricular arrhythmias or sudden death in dilated cardiomyopathy: systematic review and meta‐analysis. JACC Heart Fail 2017;5:28‐38. 28017348 10.1016/j.jchf.2016.09.017

[85] Masci PG , Schuurman R , Andrea B , Ripoli A , Coceani M , Chiappino S , *et al*. Myocardial fibrosis as a key determinant of left ventricular remodeling in idiopathic dilated cardiomyopathy: a contrast‐enhanced cardiovascular magnetic study. Circ Cardiovasc Imaging 2013;6:790‐799. doi:10.1161/CIRCIMAGING.113.000438 23934992

[86] Fontana M , Banypersad SM , Treibel TA , Maestrini V , Sado DM , White SK , *et al*. Native T1 mapping in transthyretin amyloidosis. JACC Cardiovasc Imaging 2014;7:157‐165. doi:10.1016/j.jcmg.2013.10.008 24412190

[87] Flett AS , Hayward MP , Ashworth MT , Hansen MS , Taylor AM , Elliott PM , *et al*. Equilibrium contrast cardiovascular magnetic resonance for the measurement of diffuse myocardial fibrosis: preliminary validation in humans. Circulation 2010;122:138‐144. doi:10.1161/CIRCULATIONAHA.109.930636 20585010

[88] Kirk P , Roughton M , Porter JB , Walker JM , Tanner MA , Patel J , *et al*. Cardiac T2* magnetic resonance for prediction of cardiac complications in thalassemia major. Circulation 2009;120:1961‐1968. doi:10.1161/CIRCULATIONAHA.109.874487 19801505 PMC2784198

[89] Blissett S , Chocron Y , Kovacina B , Afilalo J . Diagnostic and prognostic value of cardiac magnetic resonance in acute myocarditis: a systematic review and meta‐analysis. Int J Card Imaging 2019;35:2221‐2229. doi:10.1007/s10554-019-01674-x 31388815

[90] Gräni C , Eichhorn C , Bière L , Murthy VL , Agarwal V , Kaneko K , *et al*. Prognostic value of cardiac magnetic resonance tissue characterization in risk stratifying patients with suspected myocarditis. J Am Coll Cardiol 2017;70:1964‐1976. 29025553 10.1016/j.jacc.2017.08.050 PMC6506846

[91] Ammirati E , Moroni F , Sormani P , Peritore A , Milazzo A , Quattrocchi G , *et al*. Quantitative changes in late gadolinium enhancement at cardiac magnetic resonance in the early phase of acute myocarditis. Int J Cardiol 2017;231:216‐221. doi:10.1016/j.ijcard.2016.11.282 27913009

[92] Isaak A , Kravchenko D , Mesropyan N , Endler C , Bischoff LM , Vollbrecht T , *et al*. Layer‐specific strain analysis with cardiac MRI feature tracking in acute myocarditis. Radiol Cardiothoracic Imaging 2022;4:e210318. doi:10.1148/ryct.210318 35833169 PMC9274313

[93] Clark DE , Parikh A , Dendy JM , Diamond AB , George‐Durrett K , Fish FA , *et al*. COVID‐19 myocardial pathology evaluation in athletes with cardiac magnetic resonance (COMPETE CMR). Circulation 2021;143:609‐612. doi:10.1161/CIRCULATIONAHA.120.052573 33332151 PMC7864610

[94] Khanji MY , Archbold RA , Gallagher A , Sekhri N . Extensive myocardial scarring in idiopathic dilated cardiomyopathy: implications for management and prognosis. Eur Heart J Cardiovasc Imaging 2022;23:e267. doi:10.1093/ehjci/jeac034 35142359

[95] Khanji MY , Archbold RA , Gallagher A , Sekhri N . Systematic assessment in patients with dilated cardiomyopathy phenotype. Eur Heart J Cardiovasc Imaging 2022;23:e476. doi:10.1093/ehjci/jeac156 35900249

[96] Meister F , Passerini T , Audigier C , Lluch È , Mihalef V , Ashikaga H , *et al*. Extrapolation of ventricular activation times from sparse electroanatomical data using graph convolutional neural networks. Front Physiol 2021;18:694869. doi:10.3389/fphys.2021.694869 PMC8558498 34733172

[97] Piers SRD , Everaerts K , Van Der Geest RJ , Hazebroek MR , Siebelink HM , Pison LAFG , *et al*. Myocardial scar predicts monomorphic ventricular tachycardia but not polymorphic ventricular tachycardia or ventricular fibrillation in nonischemic dilated cardiomyopathy. Heart Rhythm 2015;12:2106‐2114. doi:10.1016/j.hrthm.2015.05.026 26004942

[98] Muser D , Santangeli P , Castro SA , Casado Arroyo R , Maeda S , Benhayon DA , *et al*. Risk stratification of patients with apparently idiopathic premature ventricular contractions: a multicenter international CMR registry. JACC Clin Electrophysiol 2020;6:722‐735. doi:10.1016/j.jacep.2019.10.015 32553224

[99] Alba AC , Gaztañaga J , Foroutan F , Thavendiranathan P , Merlo M , Alonso‐Rodriguez D , *et al*. Prognostic value of late gadolinium enhancement for the prediction of cardiovascular outcomes in dilated cardiomyopathy: an international, multi‐institutional study of the MINICOR group. Circ Cardiovasc Imaging 2020;13: doi:10.1161/CIRCIMAGING.119.010105 32312112

[100] Augusto JB , Eiros R , Nakou E , Moura‐Ferreira S , Treibel TA , Captur G , *et al*. Dilated cardiomyopathy and arrhythmogenic left ventricular cardiomyopathy: a comprehensive genotype‐imaging phenotype study. Eur Heart J Cardiovasc Imaging 2020;21:326‐336. doi:10.1093/ehjci/jez188 31317183

[101] Merlo M , Pivetta A , Pinamonti B , Stolfo D , Zecchin M , Barbati G , *et al*. Long‐term prognostic impact of therapeutic strategies in patients with idiopathic dilated cardiomyopathy: changing mortality over the last 30 years. Eur J Heart Fail 2014;16:317‐324. doi:10.1002/ejhf.16 24464640

[102] Goldberger JJ , Subačius H , Patel T , Cunnane R , Kadish AH . Sudden cardiac death risk stratification in patients with nonischemic dilated cardiomyopathy. J Am Coll Cardiol 2014;63:1879‐1889. 24445228 10.1016/j.jacc.2013.12.021

[103] Zecchin M , Merlo M , Pivetta A , Barbati G , Lutman C , Gregori D , *et al*. How can optimization of medical treatment avoid unnecessary implantable cardioverter‐defibrillator implantations in patients with idiopathic dilated cardiomyopathy presenting with “sCD‐HeFT criteria?”. Am J Cardiol 2012;109:729‐735. doi:10.1016/j.amjcard.2011.10.033 22176998

[104] Gonzalez JA , Kramer CM . Role of imaging techniques for diagnosis, prognosis and management of heart failure patients: cardiac magnetic resonance. Curr Heart Fail Rep 2015;12:276‐283. doi:10.1007/s11897-015-0261-9 26041670 PMC4496303

[105] Merlo M , Pyxaras SA , Pinamonti B , Barbati G , Di Lenarda A , Sinagra G . Prevalence and prognostic significance of left ventricular reverse remodeling in dilated cardiomyopathy receiving tailored medical treatment. J Am Coll Cardiol 2011;57:1468‐1476. doi:10.1016/j.jacc.2010.11.030 21435516

[106] Gulati A , Jabbour A , Ismail TF , Guha K , Khwaja J , Raza S , *et al*. Association of fibrosis with mortality and sudden cardiac death in patients with nonischemic dilated cardiomyopathy. JAMA 2013;309:896. doi:10.1001/jama.2013.1363 23462786

[107] Jones RE , Zaidi HA , Hammersley DJ , Hatipoglu S , Owen R , Balaban G , *et al*. Comprehensive phenotypic characterization of late gadolinium enhancement predicts sudden cardiac death in coronary artery disease. JACC Cardiovasc Imaging 2023;16:628‐638. doi:10.1016/j.jcmg.2022.10.020 36752426 PMC10151254

[108] Wahbi K , Ben Yaou R , Gandjbakhch E , Anselme F , Gossios T , Lakdawala NK , *et al*. Development and validation of a new risk prediction score for life‐threatening ventricular tachyarrhythmias in laminopathies. Circulation 2019;140:293‐302. doi:10.1161/CIRCULATIONAHA.118.039410 31155932

[109] Verstraelen TE , van Lint FHM , Bosman LP , de Brouwer R , Proost VM , Abeln BGS , *et al*. Prediction of ventricular arrhythmia in phospholamban p.Arg14del mutation carriers–reaching the frontiers of individual risk prediction. Eur Heart J 2021;42:2842‐2850. doi:10.1093/eurheartj/ehab294 34113975 PMC8325776

[110] Beggs SAS , Jhund PS , Jackson CE , McMurray JJV , Gardner RS . Non‐ischaemic cardiomyopathy, sudden death and implantable defibrillators: a review and meta‐analysis. Heart 2018;104:144‐150. doi:10.1136/heartjnl-2016-310850 28986406

[111] Køber L , Thune JJ , Nielsen JC , Haarbo J , Videbæk L , Korup E , *et al*. Defibrillator implantation in patients with nonischemic systolic heart failure. N Engl J Med 2016;375:1221‐1230. doi:10.1056/NEJMoa1608029 27571011

[112] Kadish A , Dyer A , Daubert JP , Quigg R , Estes NAM , Anderson KP , *et al*. Prophylactic defibrillator implantation in patients with nonischemic dilated cardiomyopathy. N Engl J Med 2004;350:2151‐2158. doi:10.1056/NEJMoa033088 15152060

[113] Bänsch D , Antz M , Boczor S , Volkmer M , Tebbenjohanns J , Seidl K , *et al*. Primary prevention of sudden cardiac death in idiopathic dilated cardiomyopathy: the cardiomyopathy trial (CAT). Circulation 2002;105:1453‐1458. doi:10.1161/01.CIR.0000012350.99718.AD 11914254

[114] Puwanant S , Priester TC , Mookadam F , Bruce CJ , Redfield MM , Chandrasekaran K . Right ventricular function in patients with preserved and reduced ejection fraction heart failure. Eur J Echocardiogr 2009;10:733‐737. doi:10.1093/ejechocard/jep052 19443468

[115] Verdonschot JAJ , Merken JJ , Brunner‐La Rocca HP , Hazebroek MR , Eurlings CGMJ , Thijssen E , *et al*. Value of speckle tracking–based deformation analysis in screening relatives of patients with asymptomatic dilated cardiomyopathy. JACC Cardiovasc Imaging 2020;13:549‐558. 31202754 10.1016/j.jcmg.2019.02.032

[116] Romano S , Judd RM , Kim RJ , Kim HW , Klem I , Heitner JF , *et al*. Feature‐tracking global longitudinal strain predicts death in a multicenter population of patients with ischemic and nonischemic dilated cardiomyopathy incremental to ejection fraction and late gadolinium enhancement. JACC Cardiovasc Imaging 2018;11:1419‐1429. doi:10.1016/j.jcmg.2017.10.024 29361479 PMC6043421

[117] aus de Siepen F , Buss SJ , Messroghli D , Andre F , Lossnitzer D , Seitz S , *et al*. T1 mapping in dilated cardiomyopathy with cardiac magnetic resonance: quantification of diffuse myocardial fibrosis and comparison with endomyocardial biopsy. Eur Heart J Cardiovasc Imaging 2015;16:210‐216. 25246502 10.1093/ehjci/jeu183

[118] Ochs A , Riffel J , Ochs MM , Arenja N , Fritz T , Galuschky C , *et al*. Myocardial mechanics in dilated cardiomyopathy: prognostic value of left ventricular torsion and strain. J Cardiovasc Magn Reson 2021;23:136. doi:10.1186/s12968-021-00829-x 34852822 PMC8638178

[119] Aquaro GD , Perfetti M , Camastra G , Monti L , Dellegrottaglie S , Moro C , *et al*. Cardiac MR with late gadolinium enhancement in acute myocarditis with preserved systolic function: ITAMY study. J Am Coll Cardiol 2017;70:1977‐1987. doi:10.1016/j.jacc.2017.08.044 29025554

[120] Dass S , Suttie JJ , Piechnik SK , Ferreira VM , Holloway CJ , Banerjee R , *et al*. Myocardial tissue characterization using magnetic resonance noncontrast T1 mapping in hypertrophic and dilated cardiomyopathy. Circ Cardiovasc Imaging 2012;5:726‐733. doi:10.1161/CIRCIMAGING.112.976738 23071146

[121] Puntmann VO , Carr‐White G , Jabbour A , Yu CY , Gebker R , Kelle S , *et al*. T1‐mapping and outcome in nonischemic cardiomyopathy all‐cause mortality and heart failure. JACC Cardiovasc Imaging 2016;9:40‐50. doi:10.1016/j.jcmg.2015.12.001 26762873

[122] Basser PJ . Inferring microstructural features and the physiological state of tissues from diffusion‐weighted images. NMR Biomed 1995;8:333‐344. 8739270 10.1002/nbm.1940080707

[123] Eriksson J , Bolger AF , Ebbers T , Carlhäll CJ . Assessment of left ventricular hemodynamic forces in healthy subjects and patients with dilated cardiomyopathy using 4D flow MRI. Physiol Rep 2016;4:e12685. doi:10.14814/phy2.12685 26841965 PMC4758930

[124] Raafs AG , Vos JL , Henkens MTHM , Slurink BO , Verdonschot JAJ , Bossers D , *et al*. Left atrial strain has superior prognostic value to ventricular function and delayed‐enhancement in dilated cardiomyopathy. JACC Cardiovasc Imaging 2022;15:1015‐1026. doi:10.1016/j.jcmg.2022.01.016 35680209

[125] Moldovan L , Batte KE , Trgovcich J , Wisler J , Marsh CB , Piper M . Methodological challenges in utilizing miRNAs as circulating biomarkers. J Cell Mol Med 2014;18:371‐390. doi:10.1111/jcmm.12236 24533657 PMC3943687

[126] Schroeder MA , Lau AZ , Chen AP , Gu Y , Nagendran J , Barry J , *et al*. Hyperpolarized 13C magnetic resonance reveals early‐ and late‐onset changes to in vivo pyruvate metabolism in the failing heart. Eur J Heart Fail 2013;15:130‐140. doi:10.1093/eurjhf/hfs192 23258802 PMC3547367

[127] Menichetti L , Frijia F , Flori A , Wiesinger F , Lionetti V , Giovannetti G , *et al*. Assessment of real‐time myocardial uptake and enzymatic conversion of hyperpolarized [1‐13C]pyruvate in pigs using slice selective magnetic resonance spectroscopy. Contrast Media Mol Imaging 2012;7:85‐94. doi:10.1002/cmmi.480 22344884

[128] Sluijter JPG , Davidson SM , Boulanger CM , Buzás EI , de Kleijn DPV , Engel FB , *et al*. Extracellular vesicles in diagnostics and therapy of the ischaemic heart: position paper from the Working Group on Cellular Biology of the Heart of the European Society of Cardiology. Cardiovasc Res 2018;114:19‐34. doi:10.1093/cvr/cvx211 29106545 PMC5852624

[129] Argiro A , Bui Q , Hong KN , Ammirati E , Olivotto I , Adler E . Applications of gene therapy in cardiomyopathies. JACC Heart Fail 2024;12:248‐260. doi:10.1016/j.jchf.2023.09.015 37966402

