# Supplementary material for: Dilated cardiomyopathy evaluation with Imagenomics: combining multimodal cardiovascular imaging and genetics
Source: ESC Heart Fail. 2025 Apr 24;12(4):2669–90. doi: 10.1002/ehf2.15307 (PMC12287791; doi:10.1002/ehf2.15307)
Supplement: Supplementary file 1 — Figure S1: DCM pathophysiology characteristics, seen on the ECG. Figure S2: Genetics: TTN c. 82036C > T (p.Gln27346*). CMR: LGE nonischemic pattern; septum, inferior wall, anterior wall, right ventricular insertion sites; mid myocardial and subepicardial; inflammatory process (myocarditis or sarcoidosis) PET was negative severe left atrial dilation. A: CMR LGE short axis; B: CMR elevated T1; C: CMR elevated T2; D: CMR enlarged LA; E: ECG AF rate controlled and PVCs. Figure S2. Videos ‐ TTN 2nd case found here. Figure S3: Genetics: LMNA c.1412G > A (p.Arg471His). DCM hypertrabculation, ICD, AVNRT, aorto mitral tachycardia, PVC, SVT, presyncope. CMR non‐compaction LGE basal infero‐lateral segment mid and subepicardial distribution. A: CMR LGE short axis; B: CMR LGE 2ch; C: CMR LGE 3ch; D: CMR LGE 4ch; E: ECG Sinus RBBB with occasional PVC. Figure S4: Genetics: FLNC c.6242dup (p.Ser2082Lysfs*8). aDCM, ALVC, EF35%, CMR There is basal septal thinning with dyskinesis and patchy fibrosis, PET+, AF, PVC 10%. A: CMR LGE short axis; B: CMR LGE 3ch; C: CMR LGE 4ch; D: mild myocardial inflammation involving the basal/mid lateral wall (max SUV 4) and apex (max SUV 4); E: ECG Sinus with occasional PVCs, low voltage in precordial leads. Figure S5: Genetics: FLNC c.8110_8111insC (p.Tyr2704Serfs*9). NICM, bystander CAD, PPM, VT, AFL, AF, SVT, LGE medium amount of midmyocardial LGE at basal/mid anterior and anterolateral walls, PET normal. A: CMR LGE short axis; B: CMR LGE 2ch; C: CMR LGE 3ch; D: CMR LGE 4ch; E: ECG electronic atrial and ventricular pacemaker. Figure S6: Genetics: FLNC c.1849G > T (p.Glu617*). DCM/ALVC, EF52%, CMR hypokentic LGE subepicardial, mid myocardial distribution involving the basal inferoseptal, basal inferior, mid inferoseptal, mid inferior segments, and RV insertion points.; PVC/VT, ICD, PET normal. A: CMR LGE short axis; B: CMR LGE 2ch; C: CMR LGE 3ch; D: CMR LGE 4ch; E: ECG Sinus with rare PVCs. Figure S7: Genetics: FLNC c.4926_4927insACGTCACA (p.Val1643 [file EHF2-12-2669-s001.pdf]

# Supplemental Material

## Dilated Cardiomyopathy Evaluation with Imagenomics: Combining Multimodal Cardiovascular Imaging and Genetics

### Running Title: Systematic approach to dilated cardiomyopathy diagnosis

Kristian Galanti*[1] MD, Ghaith Sharaf Dabbagh*[2] MD, Fabrizio Ricci*[1,3,4,5] MD, PhD, MSc, Sabina Gallina[1,3] MD, Roberta Giansante[5] MD, Ron Jacob[6] MD, Edmond Obeng-Gyimah[7] MD, Leslie T. Cooper Jr[8] MD, Sanjay K. Prasad[6,9,10] MD, David H. Birnie[11] MD, Andrew P. Landstrom[12] MD, PhD, MBBCh, PhD, Selma F Mohammed[13] MD, PhD, Saidi Mohiddin[14,15] MBChB, MD, MRCP, Mohammed Y. Khanji MBBCh, PhD[#14,16,17] and Anwar A. Chahal[#2,8,16]MBChB, MRCP, PhD

**Shared first authorship; #shared senior authorship*

**Affiliations**

1. Department of Neuroscience, Imaging and Clinical Sciences, G. D'Annunzio University of Chieti-Pescara, 66100 Chieti, Italy
2. Center for Inherited Cardiovascular Diseases, WellSpan Health, Lancaster, PA, USA
3. University Cardiology Division, Heart Department, SS. Annunziata Polyclinic, Chieti, Italy
4. Department of Clinical Sciences, Lund University, 214 28 Malmö, Sweden
5. Institute for Advanced Biomedical Technologies, G. D'Annunzio University of Chieti-Pescara, 66100 Chieti, Italy
6. The Heart and Vascular Institute, Lancaster General Health/Penn Medicine, Lancaster, PA, USA
7. Perelman Clinical Electrophysiology Section, Cardiovascular Division, Department of Medicine, School of Medicine at the University of Pennsylvania, Philadelphia, PA, USA
8. Division of Cardiovascular Diseases, Mayo Clinic, Rochester, MN 55905, USA
9. Department of Cardiology, Royal Brompton Hospital, London, UK
10. Department of Cardiovascular Medicine, National Heart & Lung Institute, Imperial College, London, UK
11. Department of Cardiology, University of Ottawa Heart Institute, Ottawa, Ontario, Canada
12. Division of Cardiology, Department of Pediatrics (A.P.L.), Duke University School of Medicine, Durham, NC, USA
13. Creighton University School of Medicine, Omaha, NE, USA
14. NIHR Barts Biomedical Research Centre, William Harvey Research Institute, Queen Mary University of London, EC1A 7BE, UK
15. Barts Heart Centre, St. Bartholomew's Hospital, Barts Health NHS Trust, London, UK
16. Barts Heart Centre, Barts Health NHS Trust, London, West Smithfield, EC1A 7BE, UK
17. Newham University Hospital, Barts Health NHS Trust, London E13 8SL, UK

**Address for correspondence**
C. Anwar A. Chahal, MBChB, MRCP, PhD
Center for Inherited Cardiovascular Diseases
WellSpan Health
157 North Reading Rd., Ephrata, Lancaster County, PA 17522
Phone: (717) 291-0700; Fax: (717) 291-0700
Email: cchahal@wellspan.org; chahal.anwar@mayo.edu

# Supplemental Figures:

**Supplemental Figure 1:**

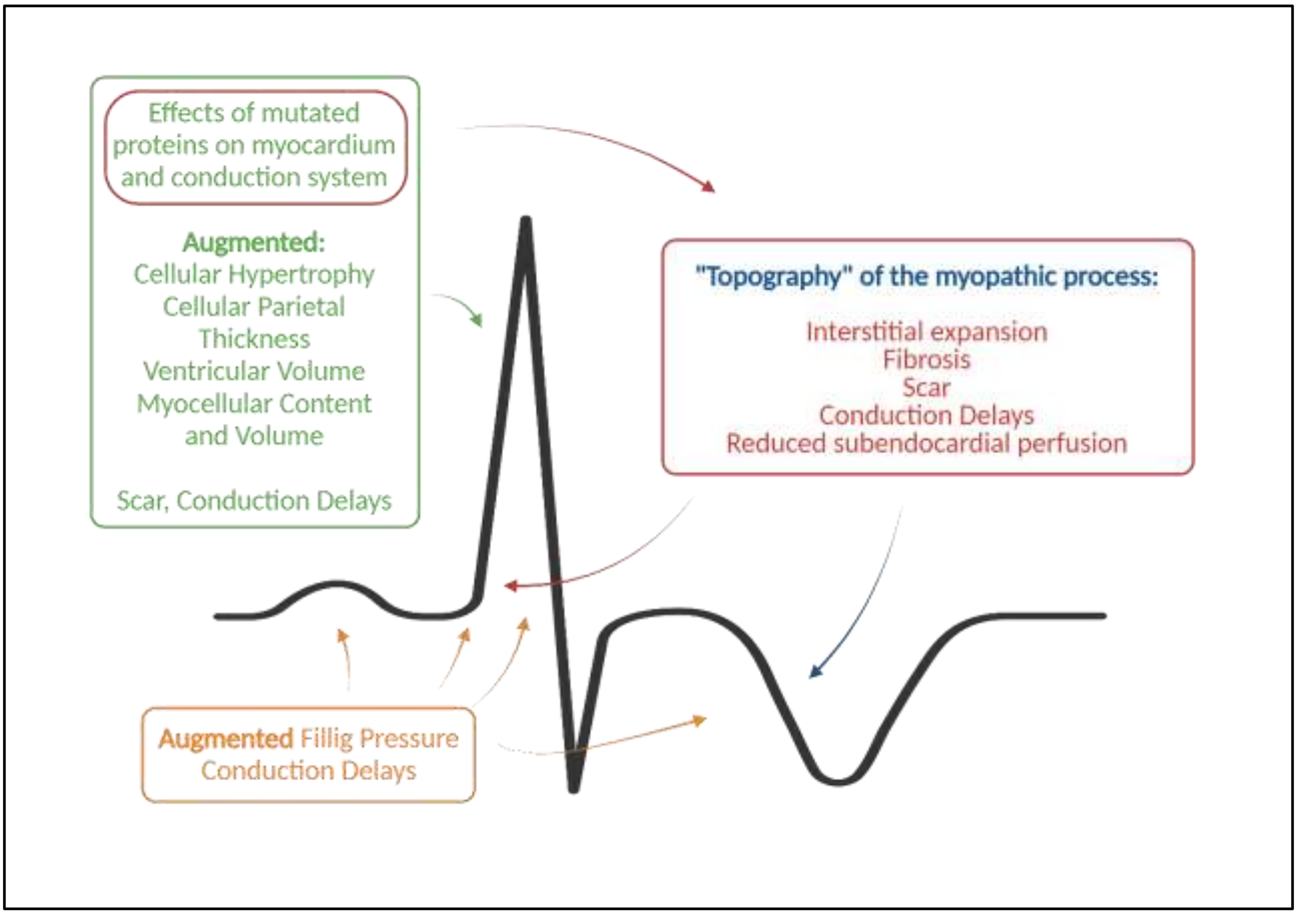


**DCM pathophysiology characteristics, seen on the ECG.**

**Supplemental Figure 2:**

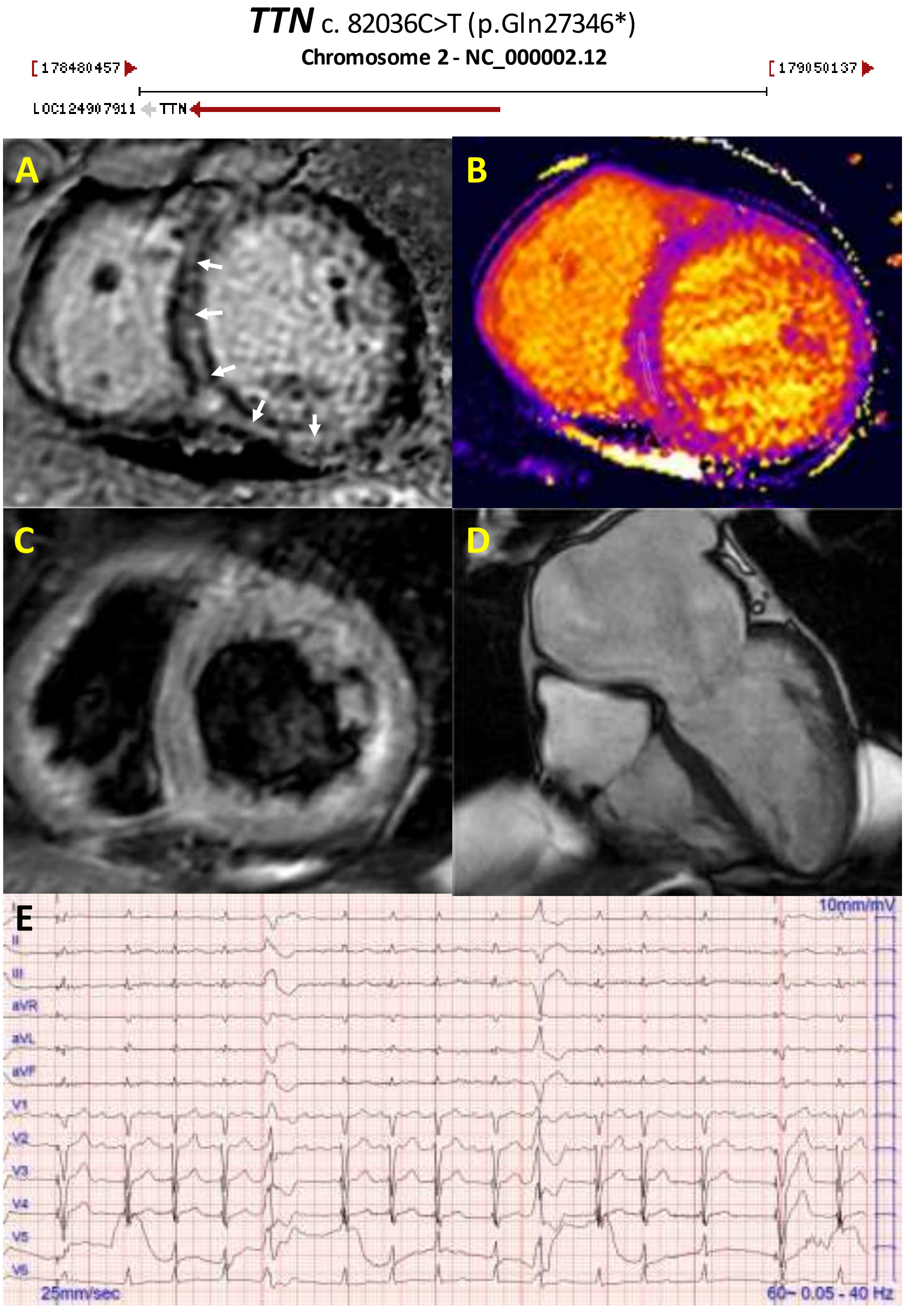


Genetics: *TTN* c. 82036C>T (p.Gln27346*). CMR: LGE nonischemic pattern; septum, inferior wall, anterior wall, right ventricular insertion sites; mid myocardial and subepicardial; inflammatory process (myocarditis or sarcoidosis) PET was negative severe left atrial dilation. **A**: CMR LGE short axis; **B**: CMR elevated T1; **C**: CMR elevated T2; **D**: CMR enlarged LA; **E**: ECG AF rate controlled and PVCs. **Figure S2 videos - TTN 2nd case found here.**

**Supplemental Figure 3:**

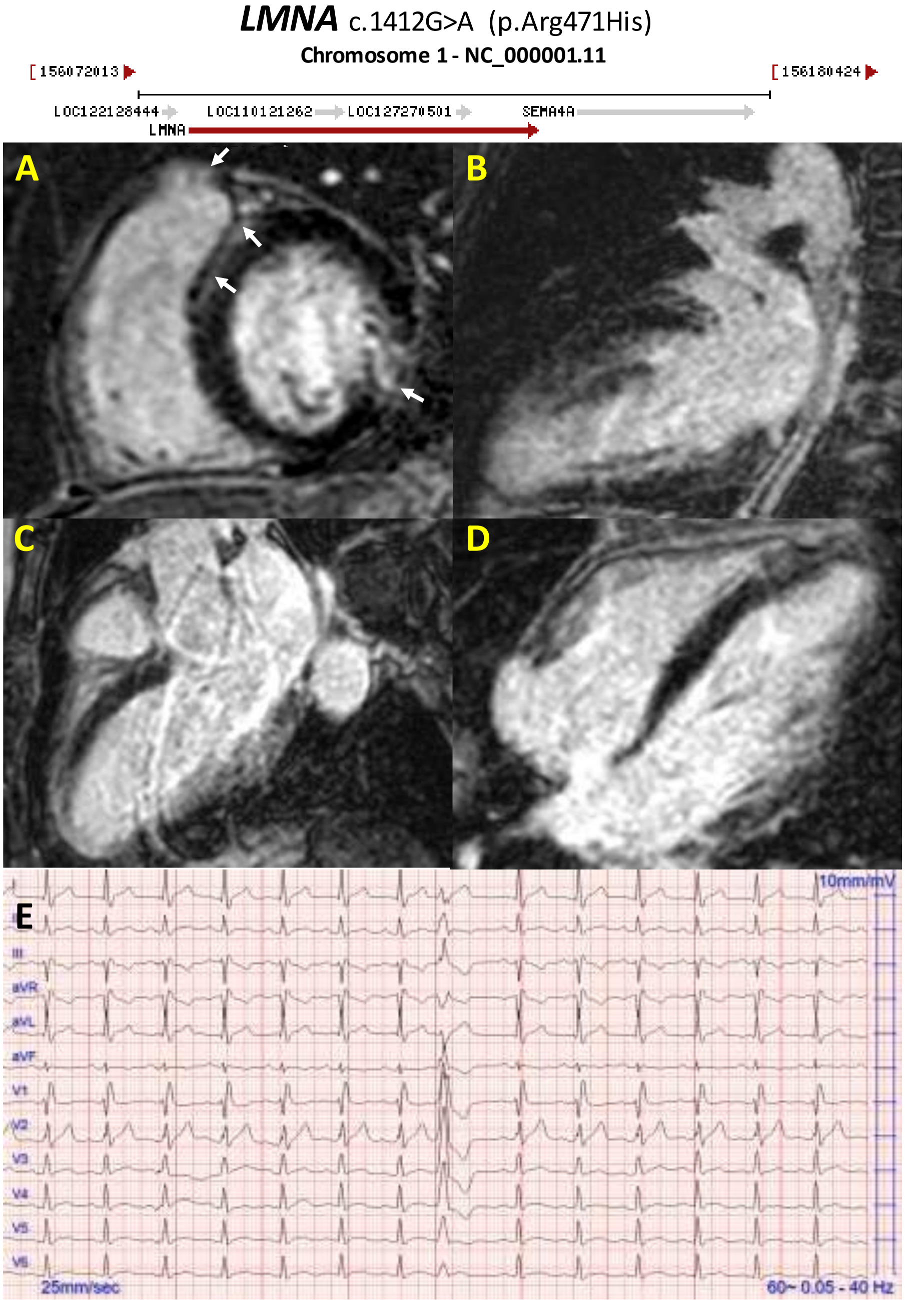


Genetics: ***LMNA*** c.1412G>A (p.Arg471His). DCM hypertrabculation, ICD, AVNRT, aorto mitral tachycardia, PVC, SVT, presyncope. CMR non-compaction LGE basal infero-lateral segment mid and subepicardial distribution. **A**: CMR LGE short axis; **B**: CMR LGE 2ch; **C**: CMR LGE 3ch; **D**: CMR LGE 4ch; **E**: ECG Sinus RBBB with occasional PVC.

**Supplemental Figure 4:**

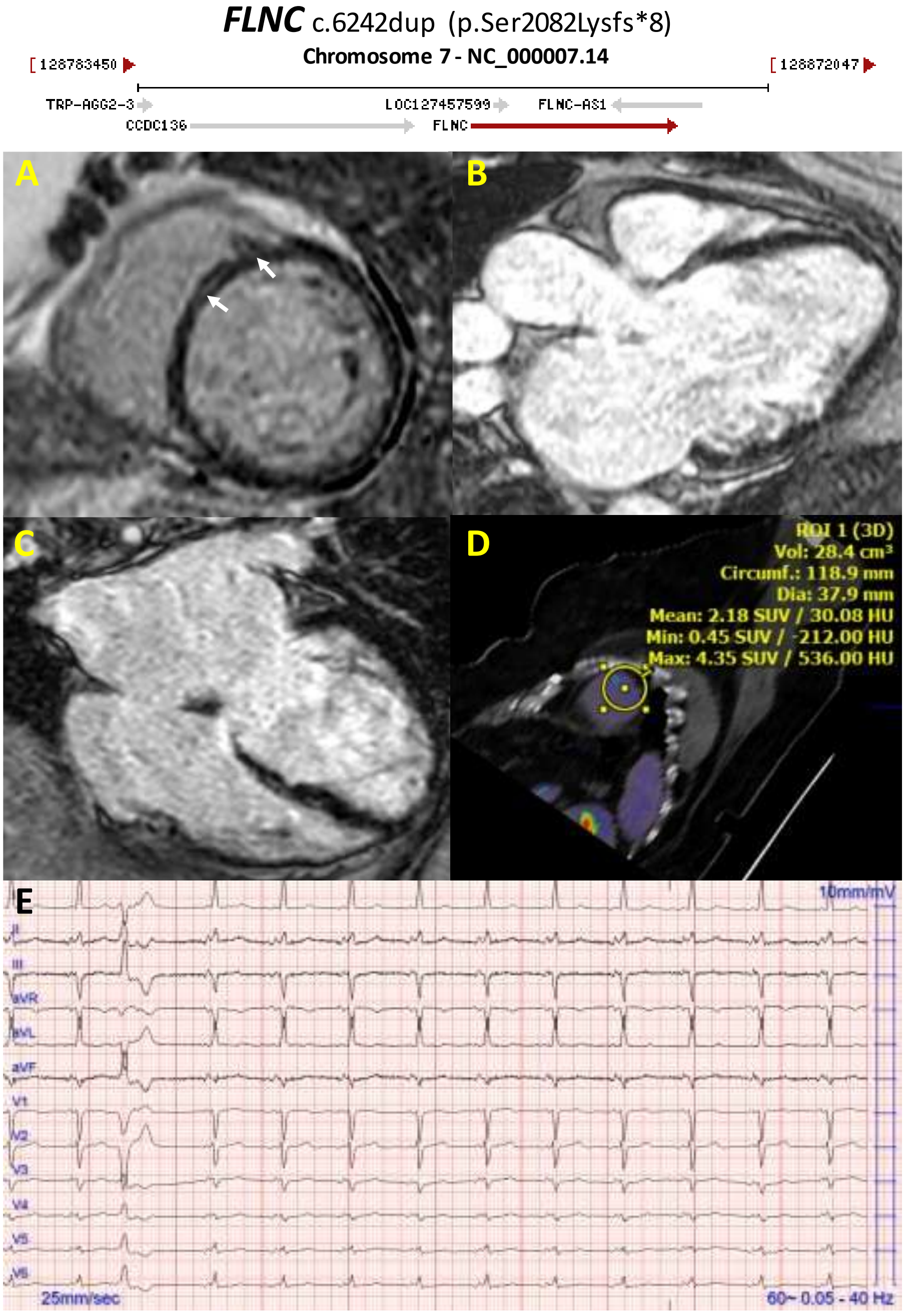


Genetics: ***FLNC*** c.6242dup (p.Ser2082Lysfs*8). aDCM, ALVC, EF35%, CMR There is basal septal thinning with dyskinesis and patchy fibrosis, PET+, AF, PVC 10%. **A**: CMR LGE short axis; **B**: CMR LGE 3ch; **C**: CMR LGE 4ch; **D**: mild myocardial inflammation involving the basal/mid lateral wall (max SUV 4) and apex (max SUV 4); **E**: ECG Sinus with occasional PVCs, low voltage in precordial leads.

**Supplemental Figure 5:**

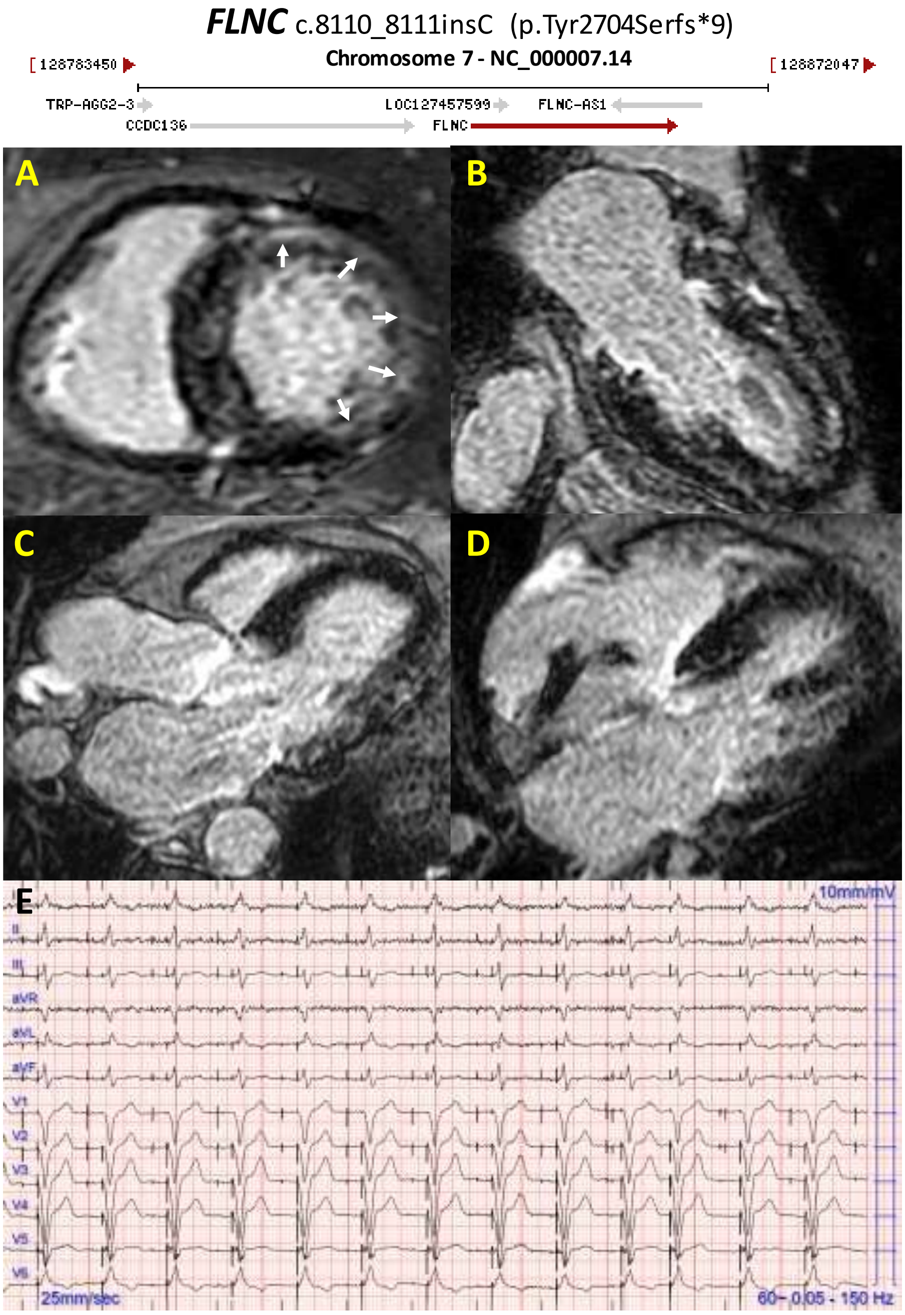


Genetics: ***FLNC*** c.8110_8111insC (p.Tyr2704Serfs*9). NICM, bystander CAD, PPM, VT, AFL, AF, SVT, LGE medium amount of midmyocardial LGE at basal/mid anterior and anterolateral walls, PET normal. **A**: CMR LGE short axis; **B**: CMR LGE 2ch; **C**: CMR LGE 3ch; **D**: CMR LGE 4ch; **E**: ECG electronic atrial and ventricular pacemaker.

**Supplemental Figure 6:**

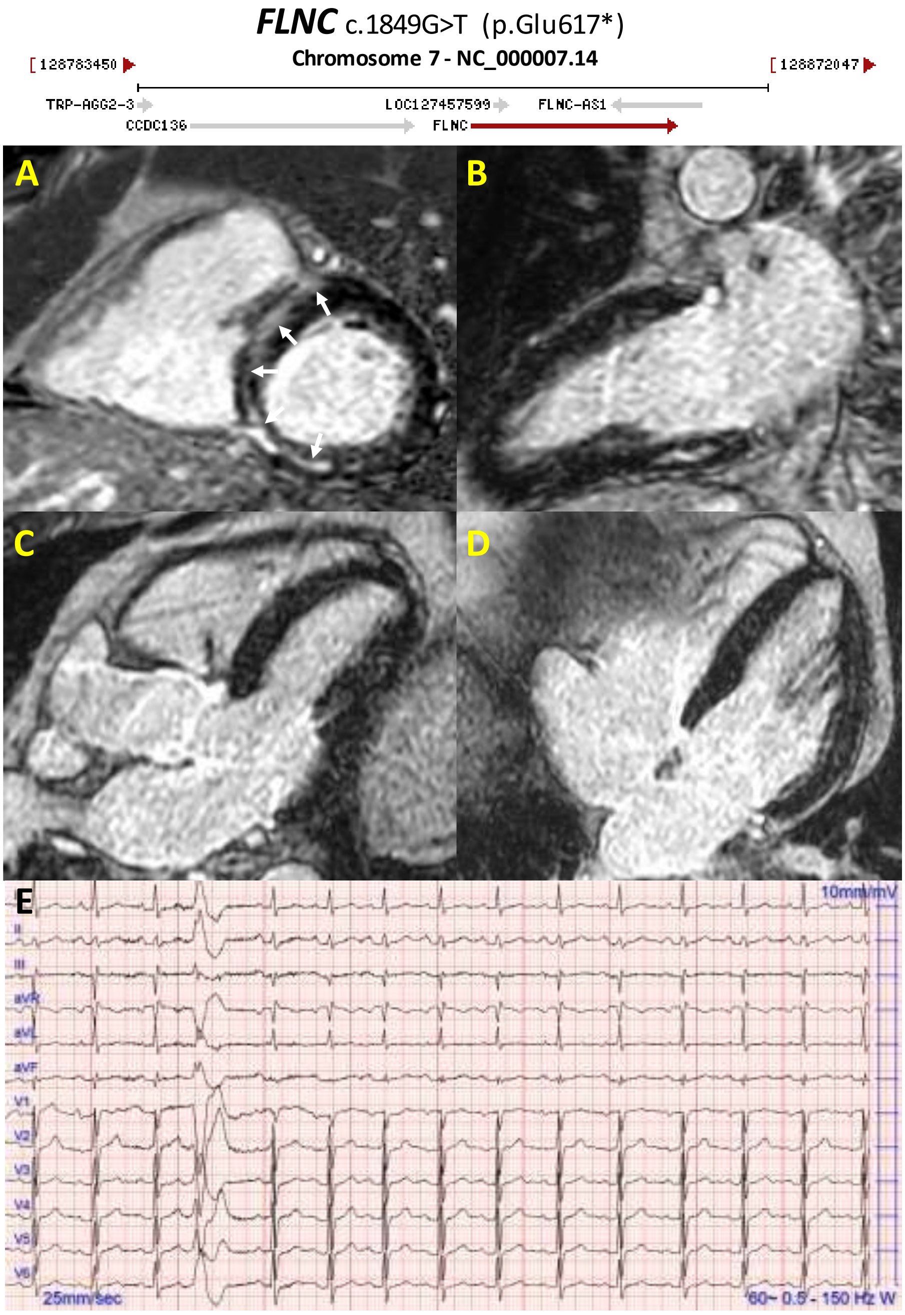


Genetics: ***FLNC*** c.1849G>T (p.Glu617*). DCM/ALVC, EF52%, CMR hypokentic LGE subepicardial, mid myocardial distribution involving the basal inferoseptal, basal inferior, mid inferoseptal, mid inferior segments, and RV insertion points.; PVC/VT, ICD, PET normal. **A**: CMR LGE short axis; **B**: CMR LGE 2ch; **C**: CMR LGE 3ch; **D**: CMR LGE 4ch; **E**: ECG Sinus with rare PVCs.

**Supplemental Figure 7:**

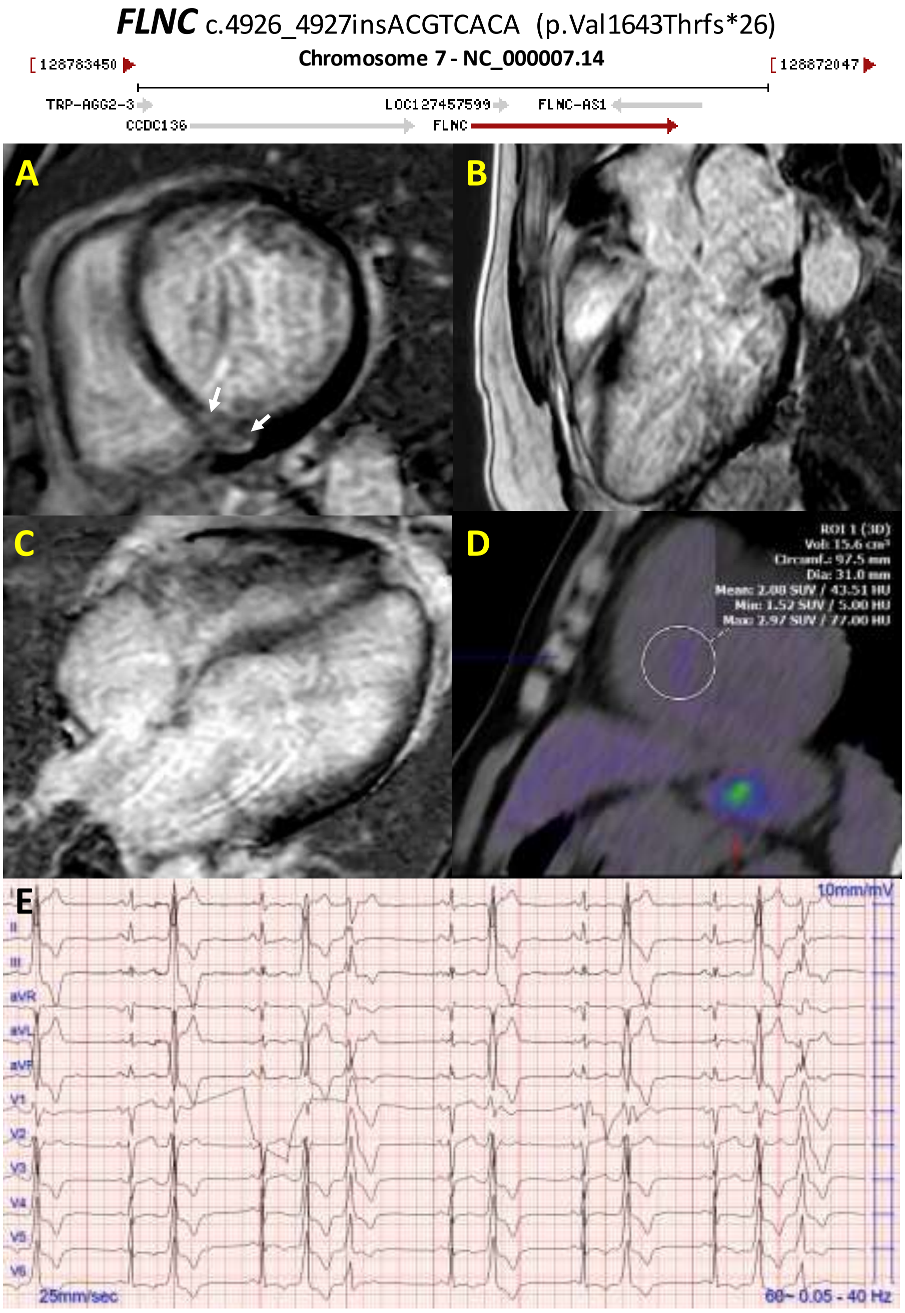


Genetics: ***FLNC*** c.4926_4927insACGTCACA (p.Val1643Thrfs*26). NICM, EF30%, LGE in small amount RV insertion, PET+ same CMR, PVC(LVOT), MVP, SVT/AVNRT, VT(RVOT). **A**: CMR LGE short axis; **B**: CMR LGE 3ch; **C**: CMR LGE 4ch; **D**: small amount of active myocardial inflammation involving the mid inferoseptal wall, max SUV 2.97 ); **E**: ECG Sinus with frequent PVCs.

**Supplemental Figure 8:**

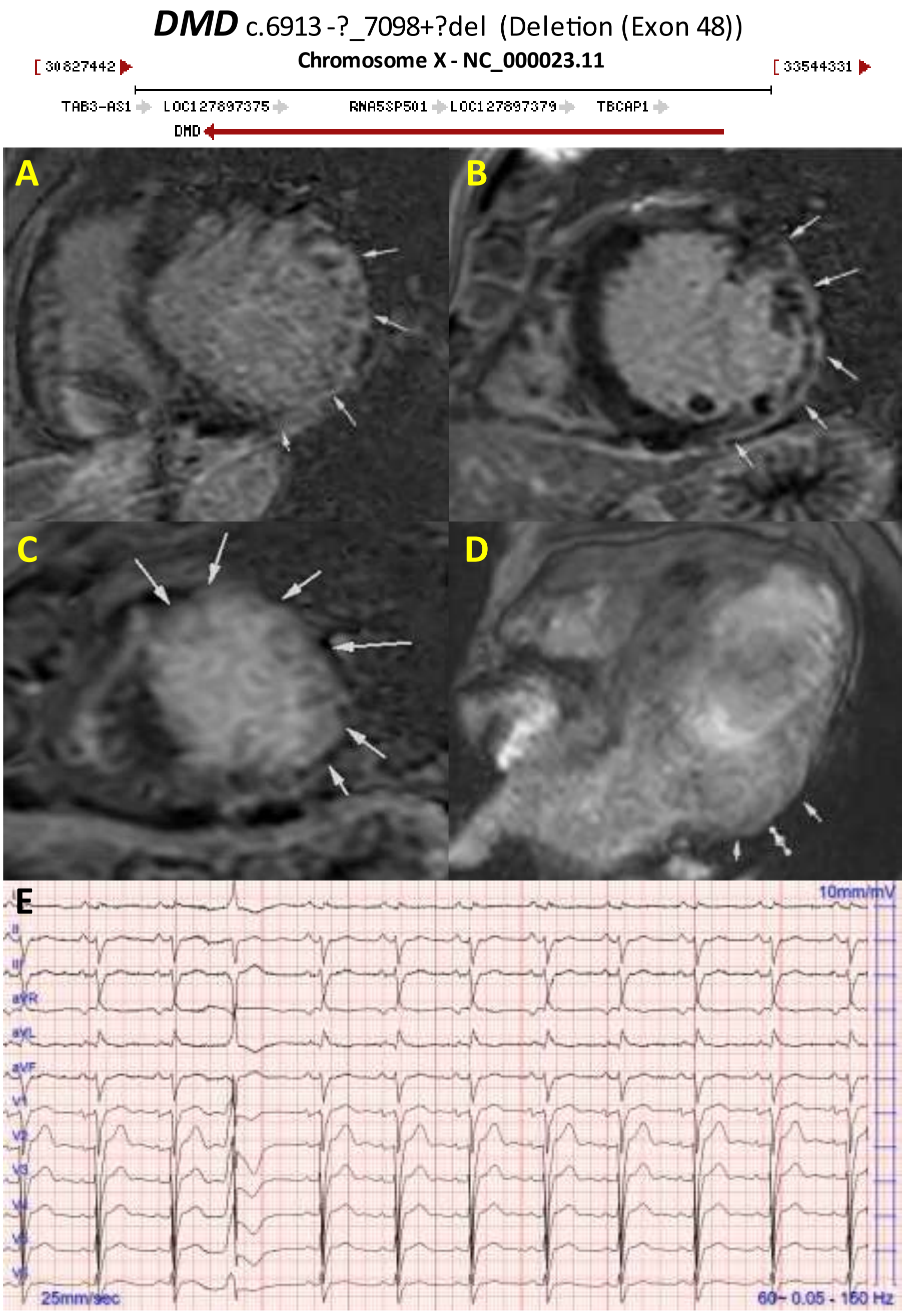


Genetics: ***DMD*** c.6913-?_7098+?del (Deletion (Exon 48). NICM, VT, ICD, HFrEF, CMR hypokinetic LGE Extensive enhancement of the lateral wall with both transmural and subepicardial components, with extension into adjacent anterior and inferior segments; PET same as CMR. **A**: CMR LGE basal short axis; **B**: CMR LGE mid short axis; **C**: CMR LGE apical short axis; **D**: CMR LGE 4ch; **E**: ECG Sinus with occasional PVCs, intraventricular conduction delay, left axis deviation.

**Supplemental Figure 9:**

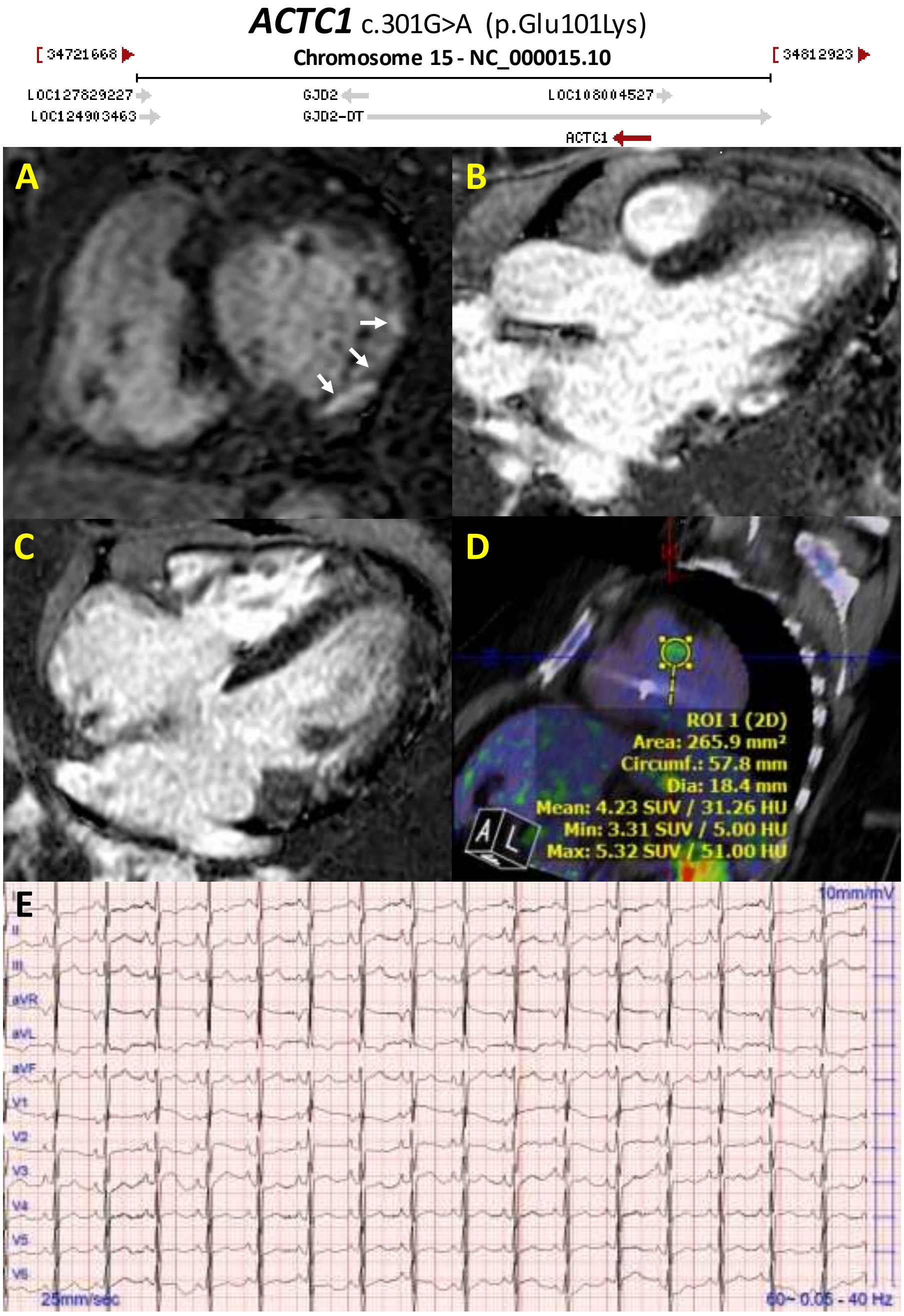


Genetics: ***ACTC1*** c.301G>A (p.Glu101Lys). CMR: There is small amount of midmyocardial late gadolinium enhancement (LGE) at the basal inferolateral wall. Overall, findings are suggestive of an infiltrative process such as Fabry's disease or cardiac sarcoidosis. Prior viral myocarditis can also have similar pattern in the correct clinical context. Danon disease is possible although less likely given LGE pattern. **A**: CMR LGE short axis; **B**: CMR LGE 3ch; **C**: CMR LGE 4ch; **D**: CT-PET uptake along the basal to mid anteroseptum at the RV insertion point (max SUV 5.3), basal to mid inferior septum; **E**: Sinus tachycardia, Incomplete right bundle branch block, Left anterior fascicular block, Left ventricular hypertrophy and ST-T change.

**Supplemental Figure 10:**

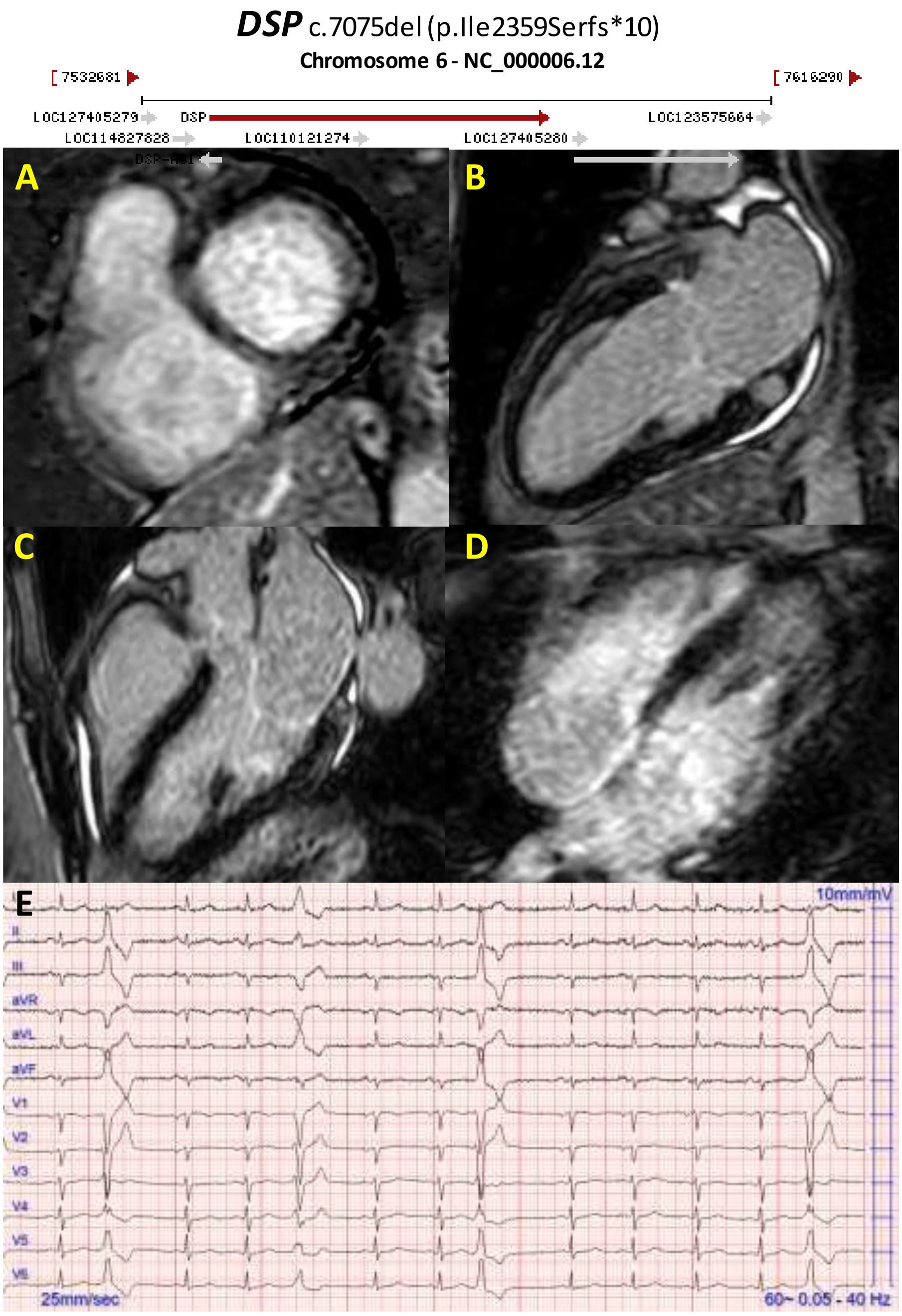


Genetics: ***DSP*** c.7075del(p.Ile2359Serfs*10). PVC (RVOT) ablated, early NICM (arrythmia), AF; PET normal; CMR: no LGE. **A**: CMR LGE short axis; **B**: CMR LGE 2ch; **C**: CMR LGE 3ch; **D**: CMR LGE 4ch; **E**: Sinus rhythm with PVCs, there is poor R-wave progression.

**Supplemental Figure 11:**

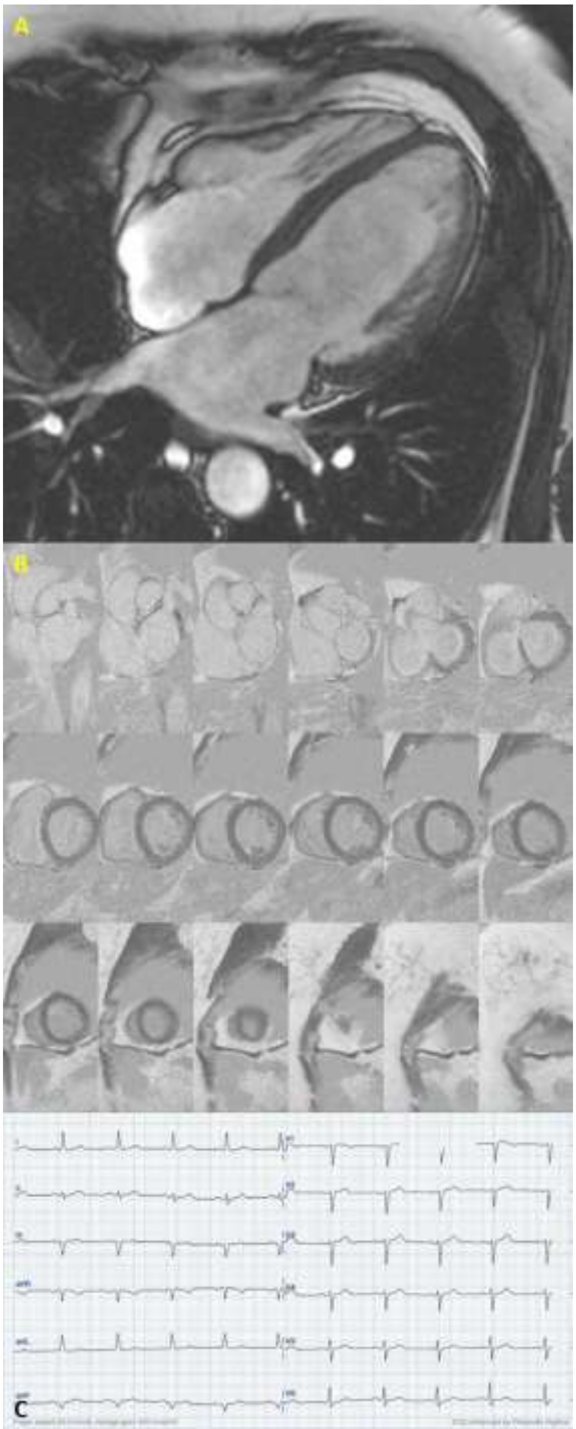

Genetics: ***MYH7*** c.3865C>T (p.Arg1289Trp). Dilated cardiomyopathy (LVEDVi 112 ml/m2; LVEDV 219 ml) with basal septum midwall LGE. **A**: CMR LGE 4 chamber view; **B**: CMR LGE short axis multislice view; **C**: ECG showing sinus rhythm and reduced R wave progression in the precordial leads.

**Supplemental Figure 12:**

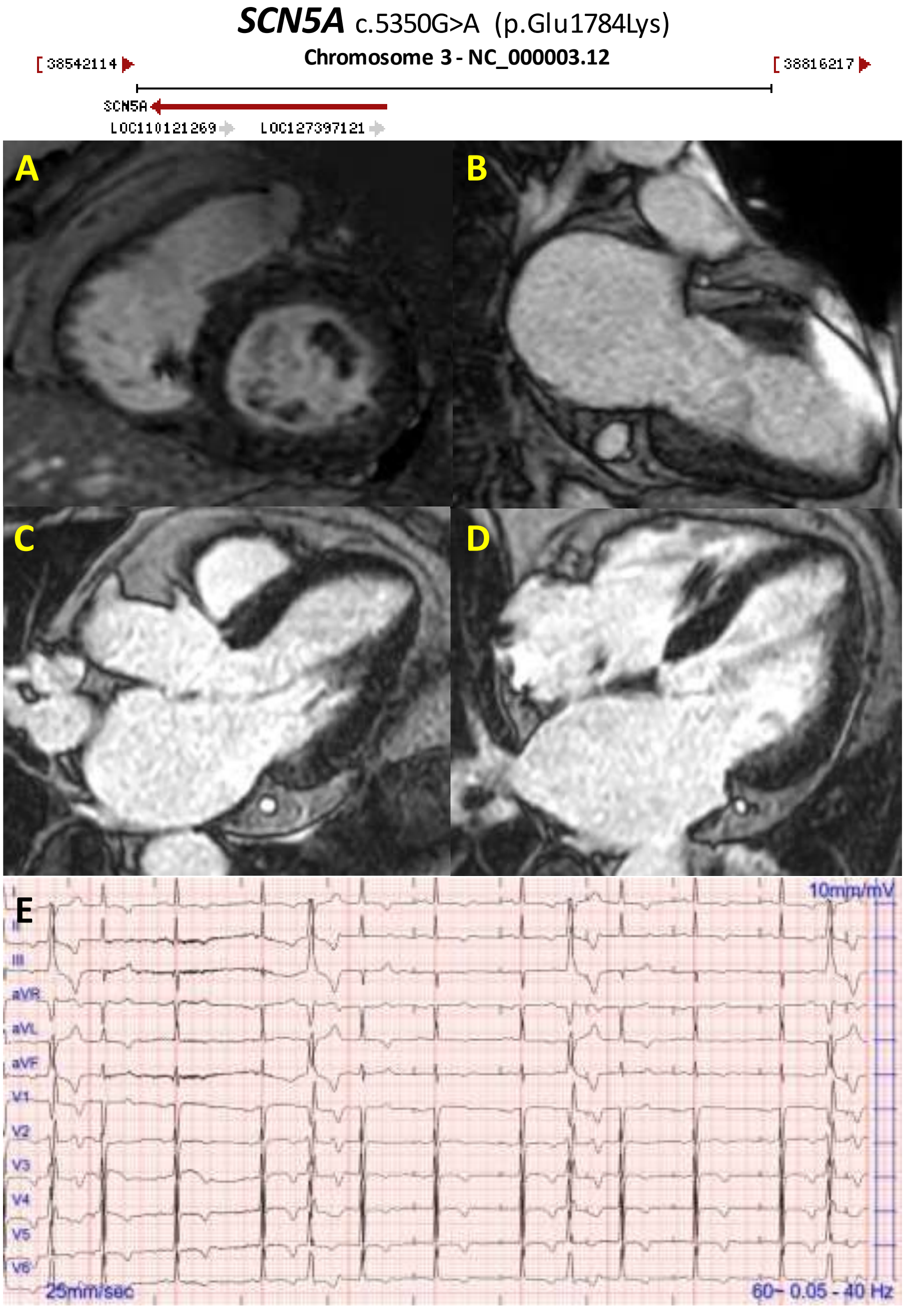


Genetics: ***SCN5A*** c.5350G>A (p.Glu1784Lys). LQTS 3, PVC/VF/PMVT/storm, ICD, CMR: delayed enhancement sequences are suboptimal due to a susceptibility artifact over the mid and distal anterior wall secondary to pacemaker however no obvious evidence of an infiltrative cardiomyopathy or ischemic damage in the segments visualized. No obvious evidence of myocardial edema in the visualized segments of the myocardium. **A**: CMR LGE short axis; **B**: CMR LGE 2ch; **C**: CMR LGE 3ch; **D**: CMR LGE 4ch; **E**: ECG Electronic atrial pacemaker PVC, Moderate T-wave abnormality, LQTS 3.

**Supplemental Figure 13:**

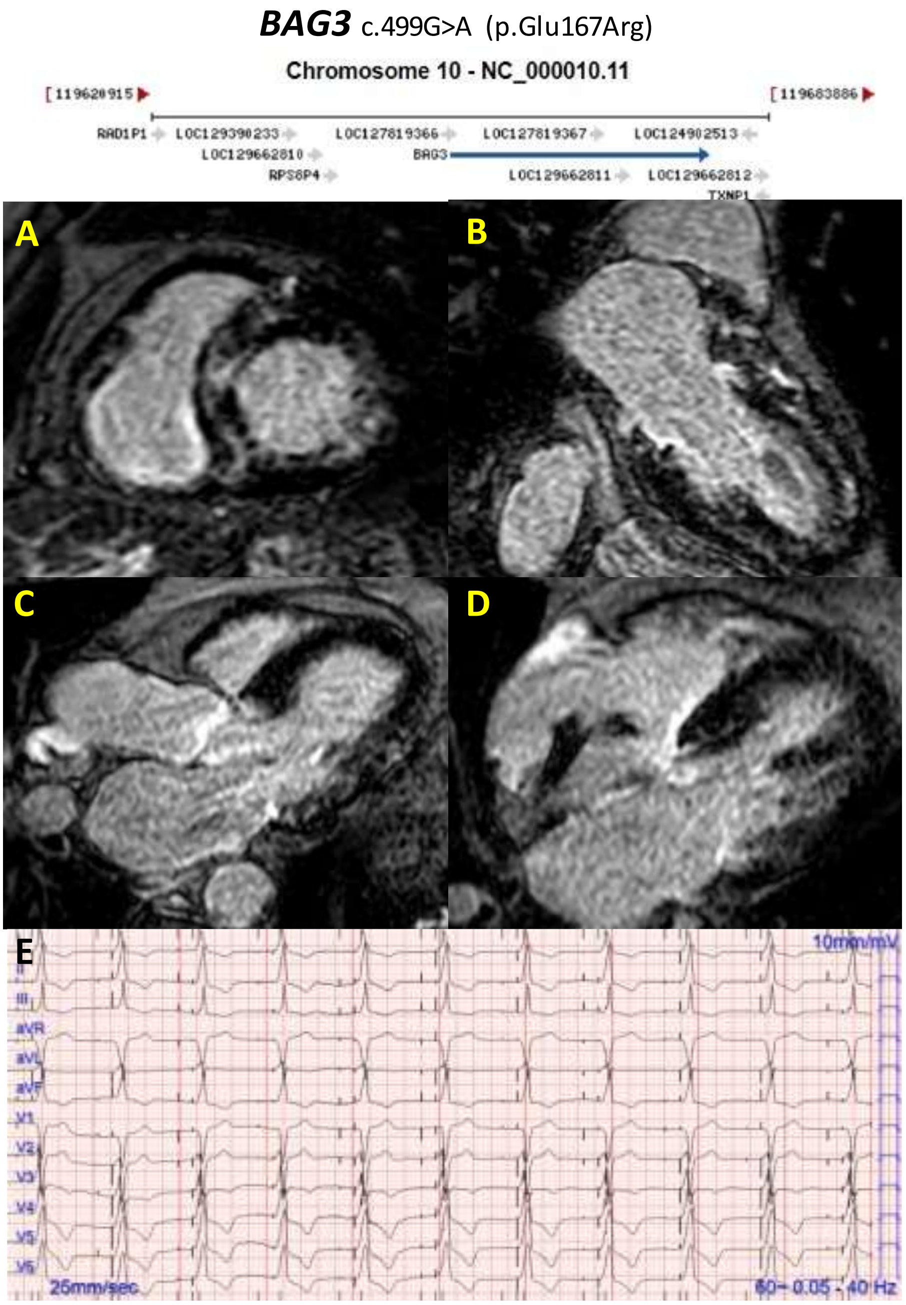


Genetics: ***BAG3*** c.499G>A (p.Glu167Arg). **A**: CMR LGE short axis; **B**: CMR LGE 2ch; **C**: CMR LGE 3ch; **D**: CMR LGE 4ch; **E**: ECG.

**Supplemental Figure 14:**

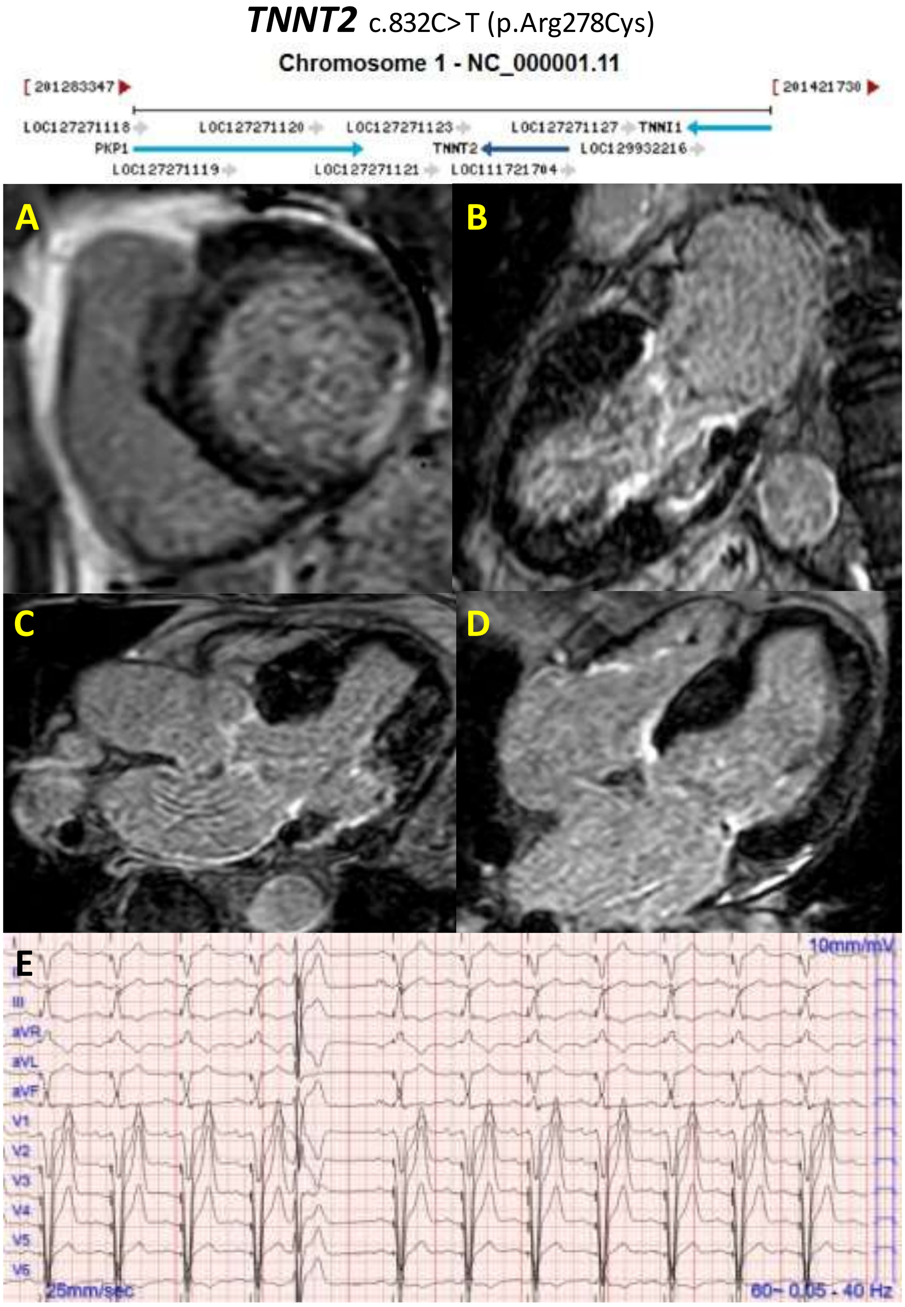


Genetics: ***TTNT2*** c.832C>T (p.Arg278Cys). **A**: CMR LGE short axis; **B**: CMR LGE 2ch; **C**: CMR LGE 3ch; **D**: CMR LGE 4ch; **E**: ECG.

**Supplemental Figure 15:**

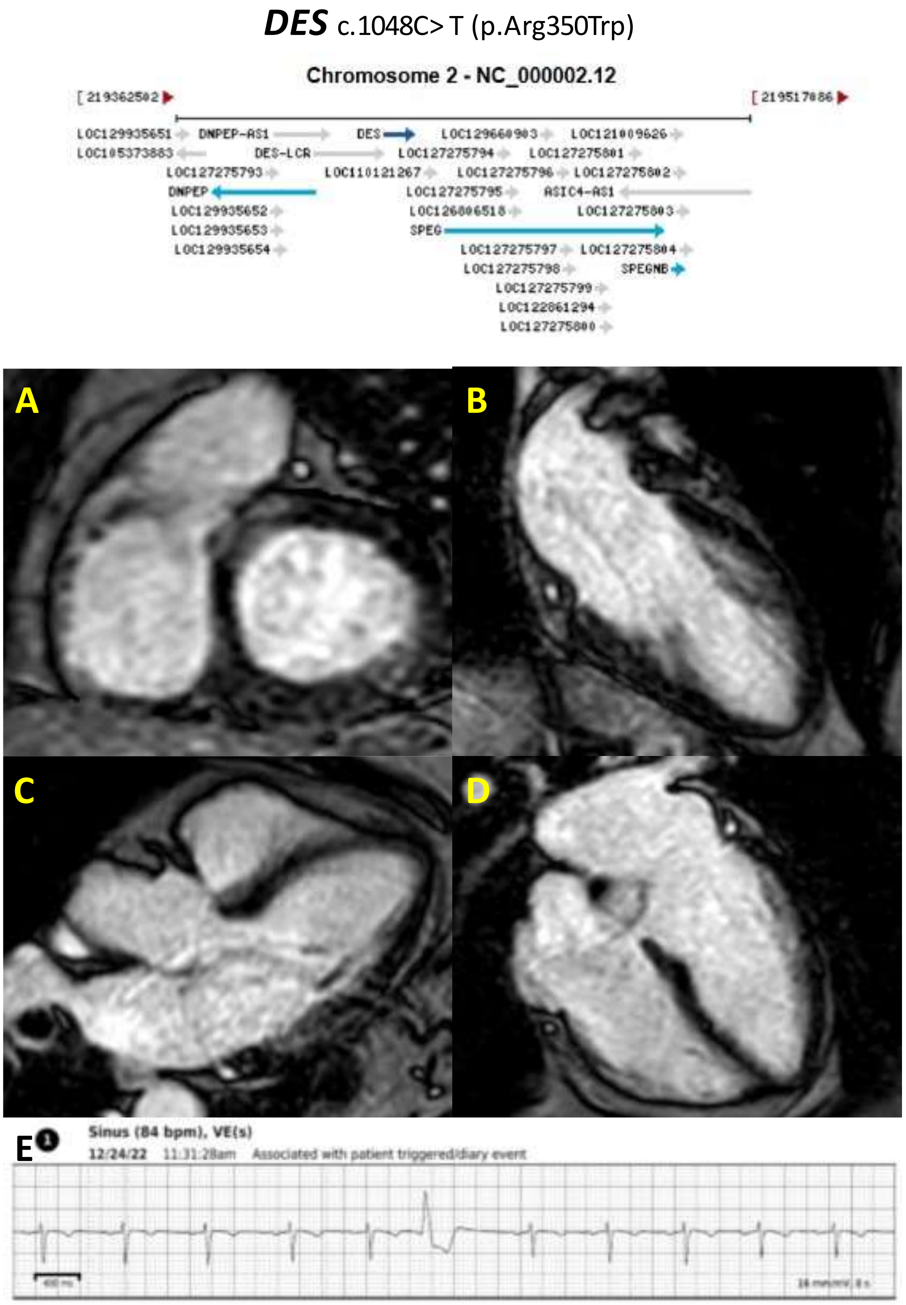


Genetics: ***DES*** c.1048C>T (p.Arg350Trp). **A**: CMR LGE short axis; **B**: CMR LGE 2ch; **C**: CMR LGE 3ch; **D**: CMR LGE 4ch; **E**: ECG.

**Supplemental Figure 16:**

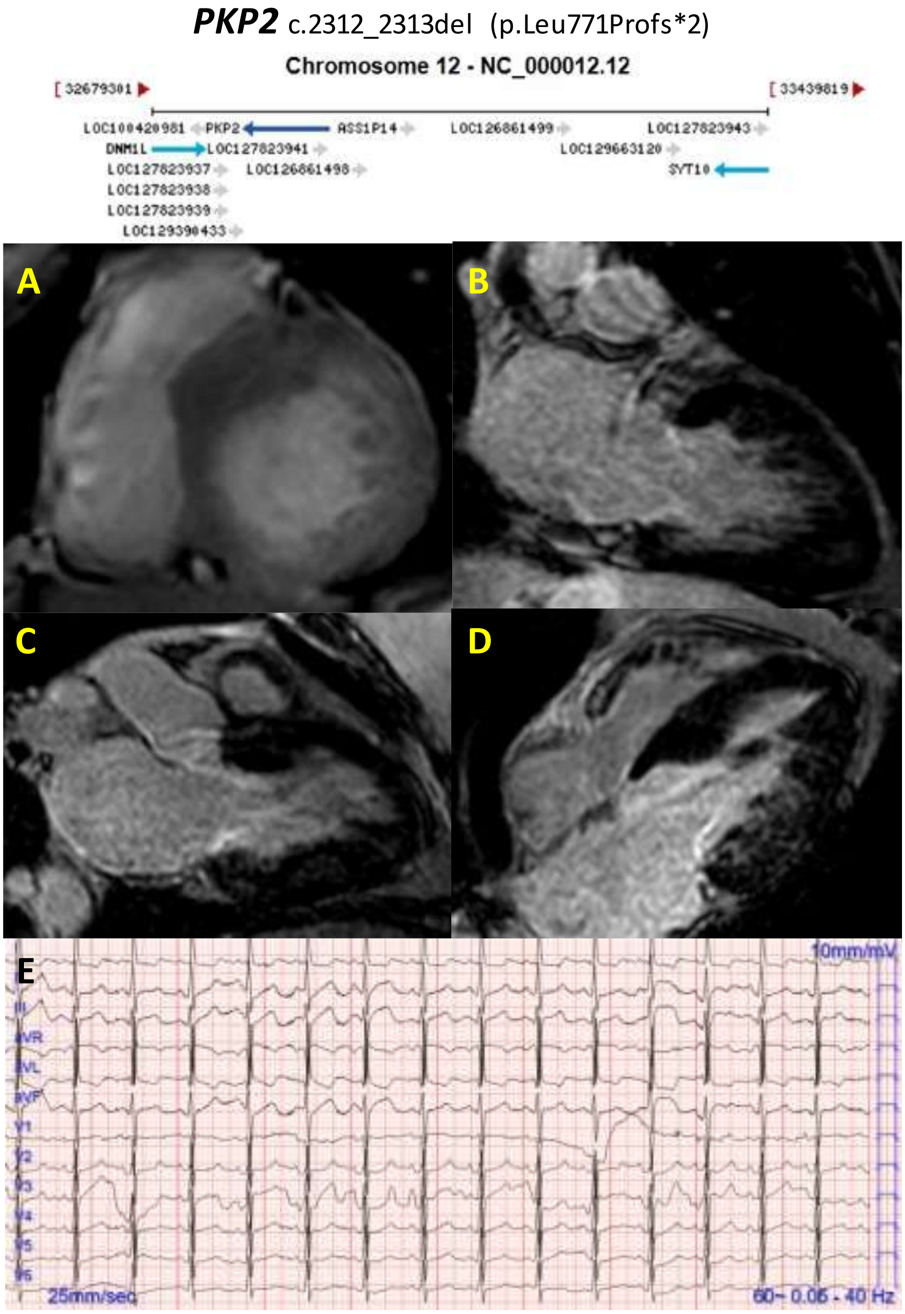


Genetics: ***PKP2*** c.2312_2313del (p.Leu771Profs*2). **A**: CMR LGE short axis; **B**: CMR LGE 2ch; **C**: CMR LGE 3ch; **D**: CMR LGE 4ch; **E**: ECG.

**Supplemental Figure 17:**

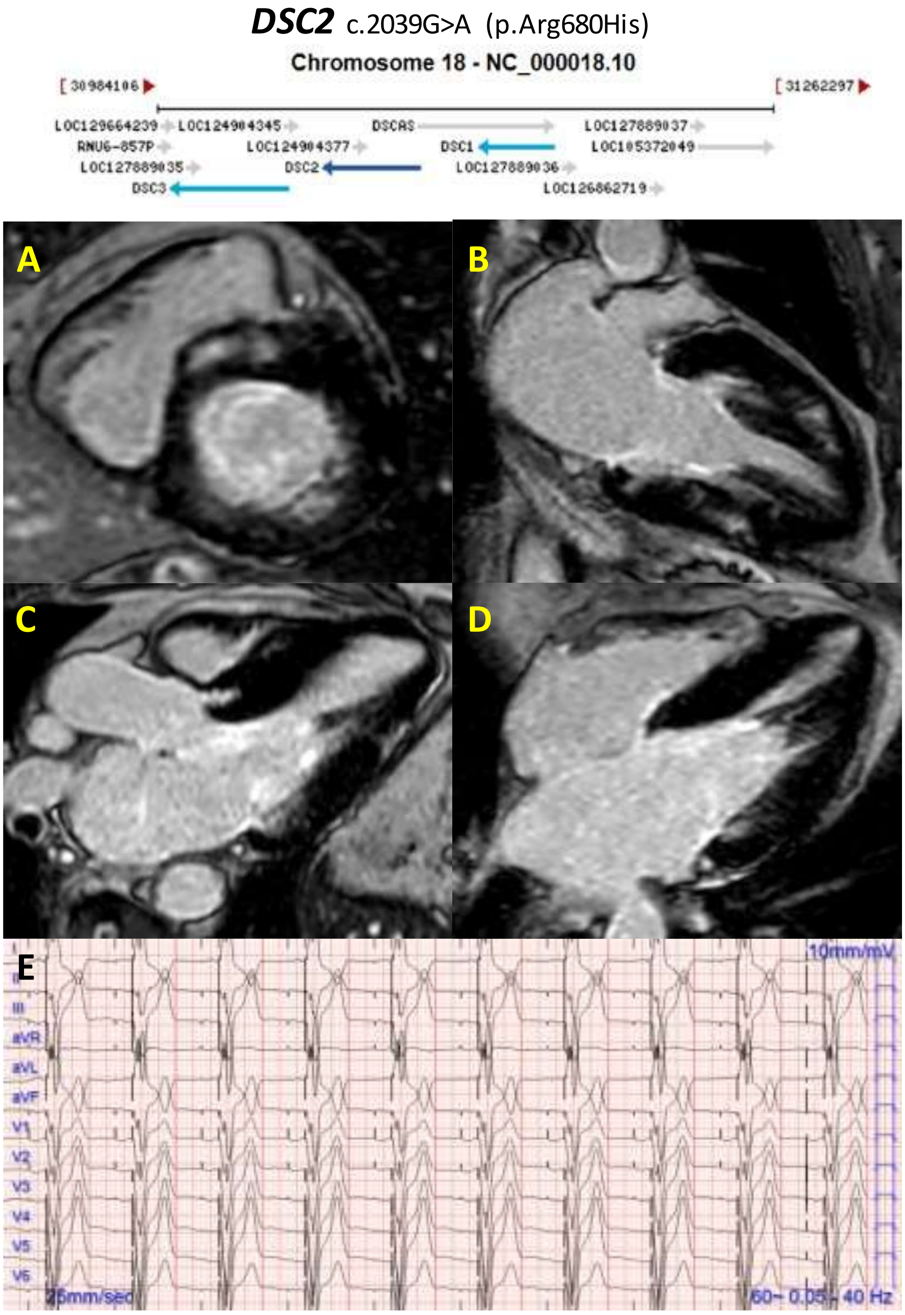


Genetics: ***DSC2*** c.2039G>A (p.Arg680His). **A**: CMR LGE short axis; **B**: CMR LGE 2ch; **C**: CMR LGE 3ch; **D**: CMR LGE 4ch; **E**: ECG.

**Supplemental Figure 18:**

***DSG2*** c.146G>A (p.Arg49His)

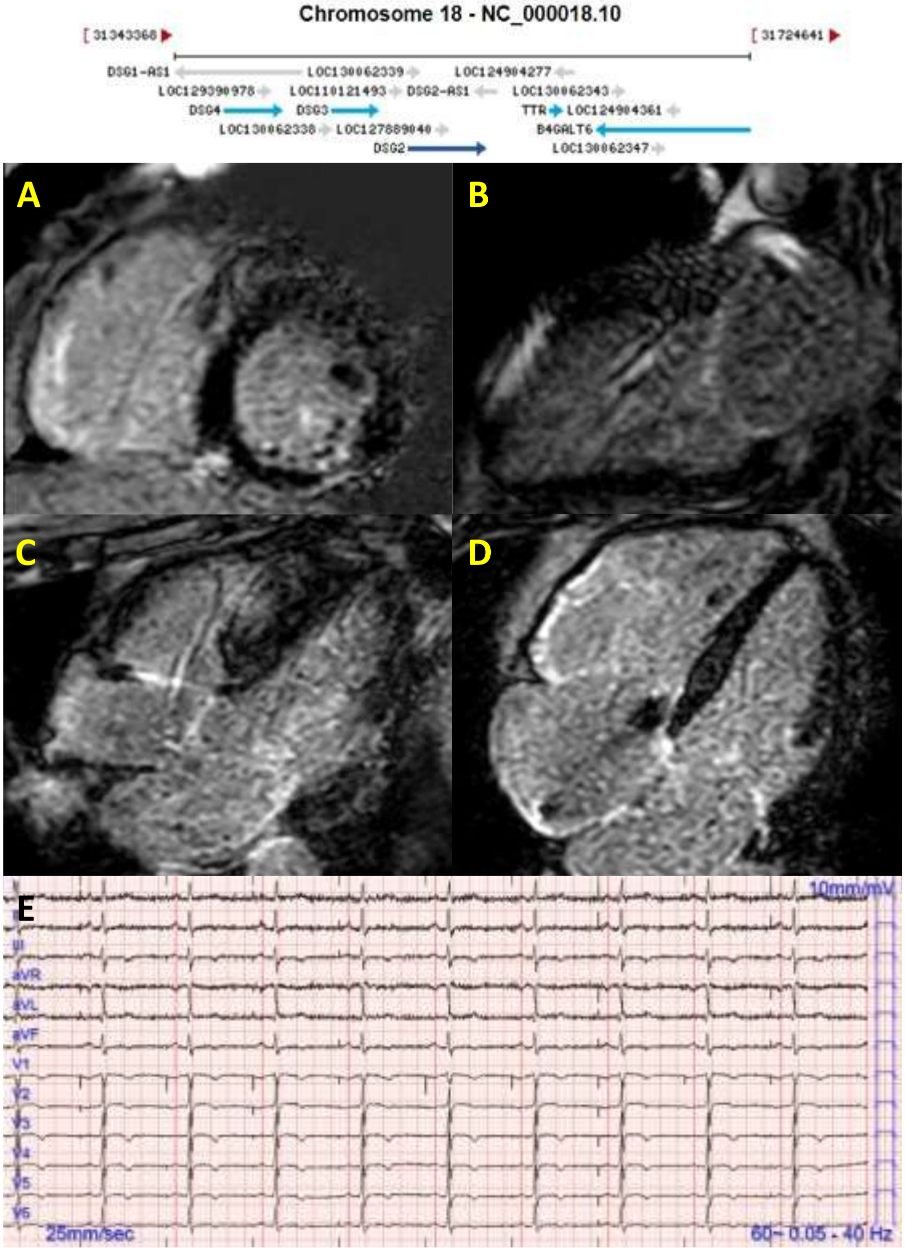


Genetics: ***DSG2*** c.146G>A (p.Arg49His). **A**: CMR LGE short axis; **B**: CMR LGE 2ch; **C**: CMR LGE 3ch; **D**: CMR LGE 4ch; **E**: ECG.

**Supplemental Figure 19:**

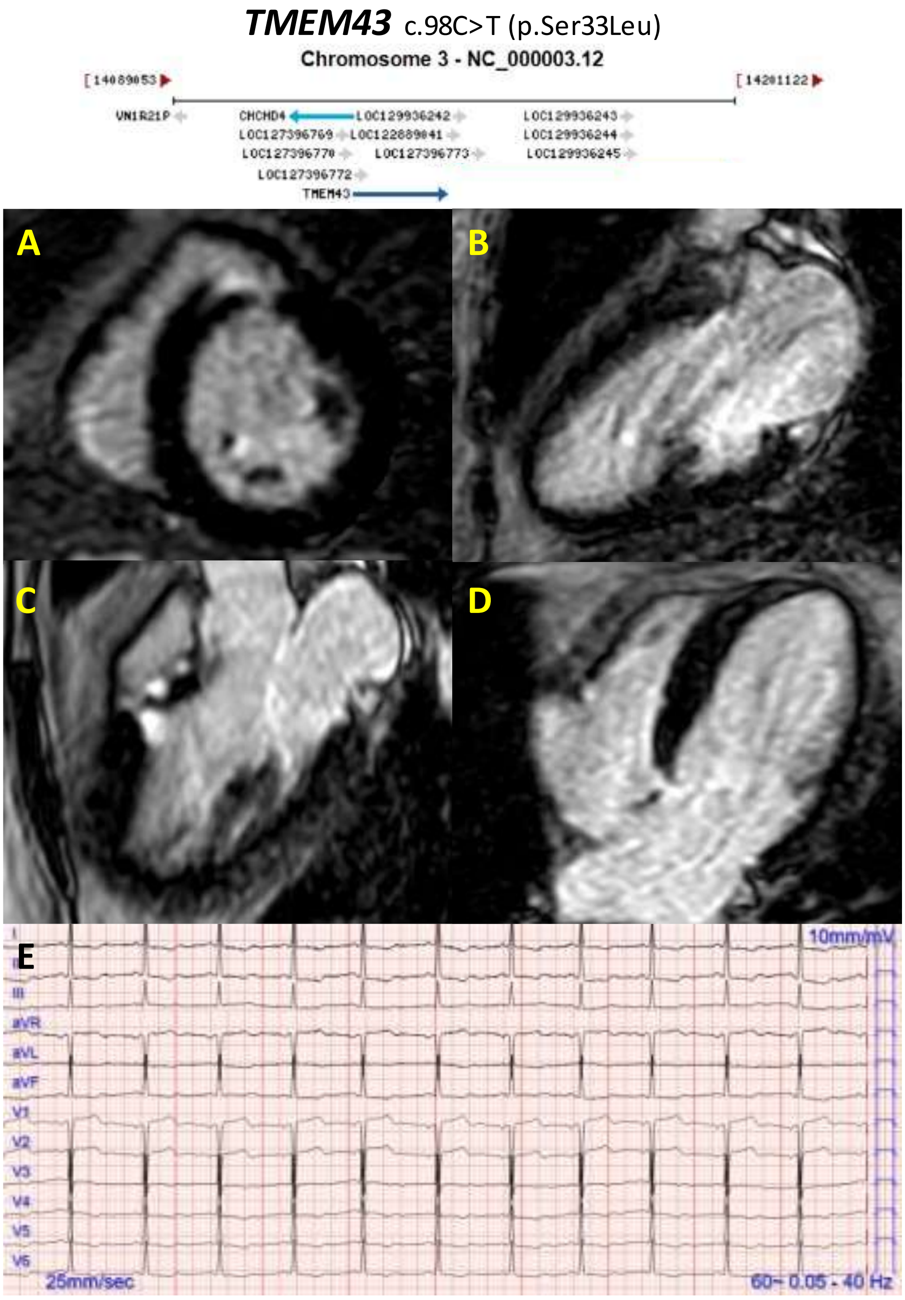


Genetics: ***TMEM43*** c.98C>T (p.Ser33Leu). **A**: CMR LGE short axis; **B**: CMR LGE 2ch; **C**: CMR LGE 3ch; **D**: CMR LGE 4ch; **E**: ECG.

**Supplemental Figure 20:**

***JUP*** c.509C>T (p.Ser170Leu)

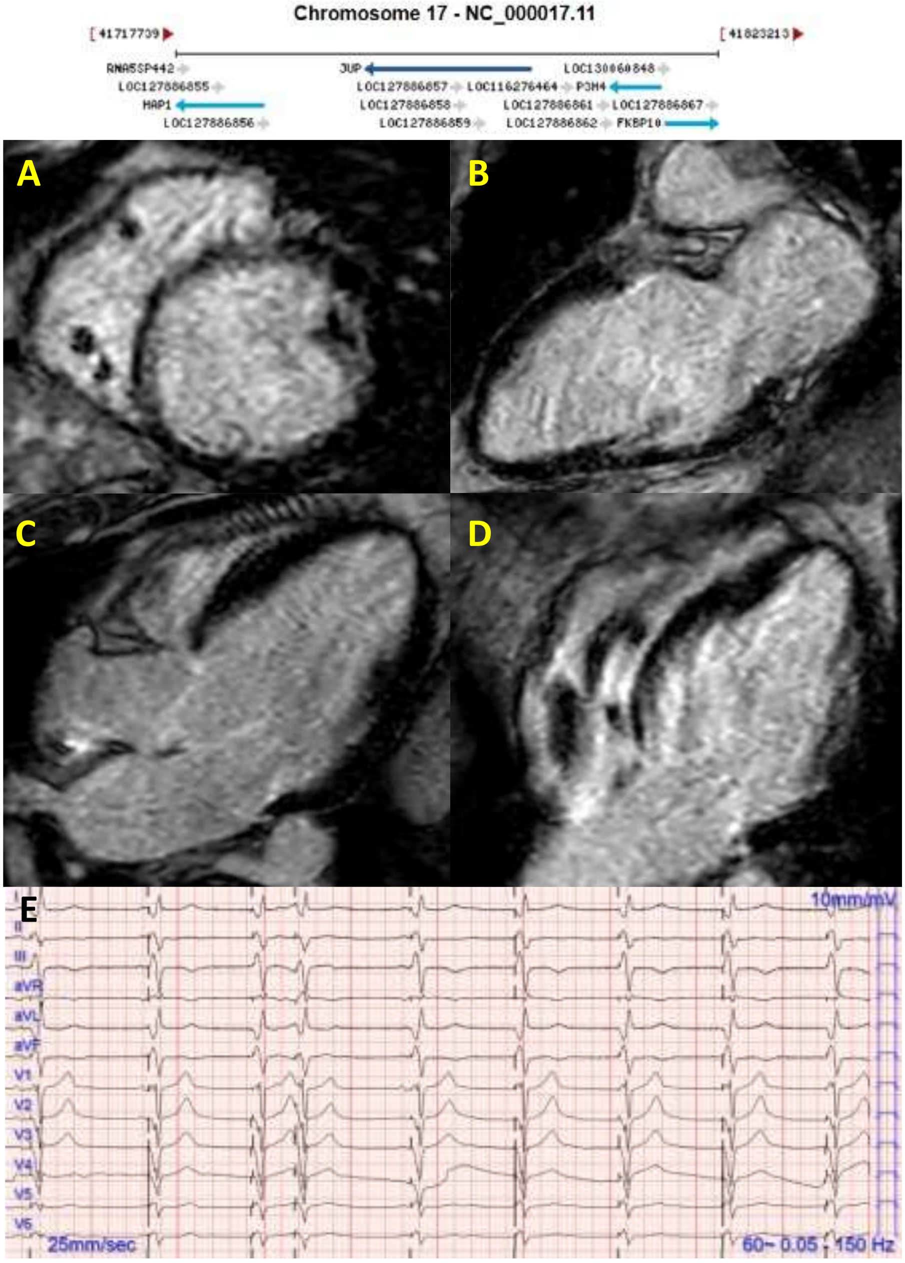


Genetics: ***JUP*** c.509C>T (p.Ser170Leu). **A**: CMR LGE short axis; **B**: CMR LGE 2ch; **C**: CMR LGE 3ch; **D**: CMR LGE 4ch; **E**: ECG.

**Supplemental Figure 21:**

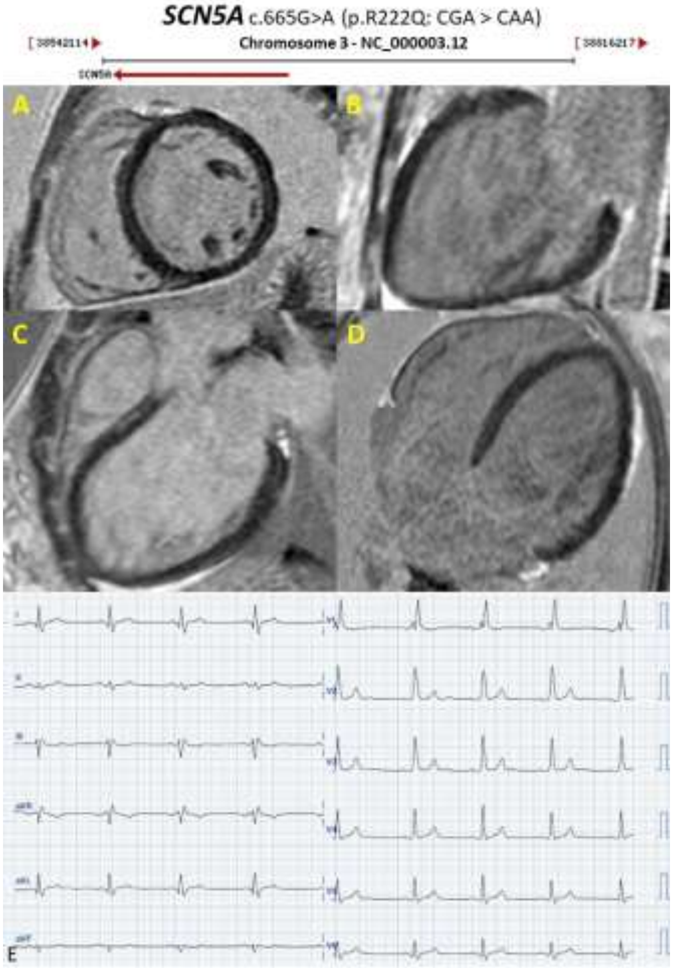


Genetics: ***SCN5A*** c.665G>A (p.R222Q: CGA > CAA). 24-year-old man, black, referred to CMR for excessive trabeculations and dilated phenotype by echocardiogram. **A**: CMR LGE short axis showing mild midwall circumferential LGE of basal segments; **B**: CMR LGE 2ch; **C**: CMR LGE 3ch; **D**: CMR LGE 4ch; **E**: ECG.

**Supplemental Figure 22**

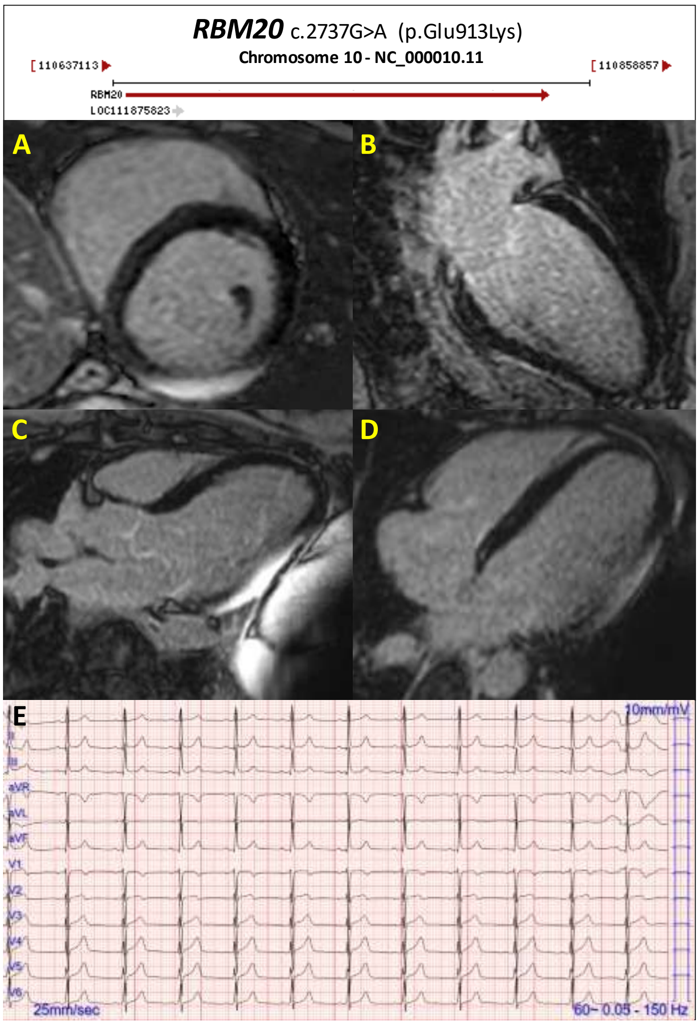


**RBM20 Mutation in patient with COVID-related myocarditis.** The evaluation of the inferior wall is limited due to artifact from the subcutaneous pacemaker however no obvious delayed enhancement noted in the visualized segments of the myocardium. No myocardial edema by T2-weighted imaging noted in the visualized segments of the cardia. Genetics: ***RBM20*** c.2737G>A (p.Glu913Lys). **A**: CMR LGE short axis; **B**: CMR LGE 2ch; **C**: CMR LGE 3ch; **D**: CMR LGE 4ch; **E**: ECG Ventricular tachycardia, LBBB. *CMR: Cardiac Magnetic Resonance; LBBB: Left Bundle Branch Block.*

**Supplemental Figure 23**

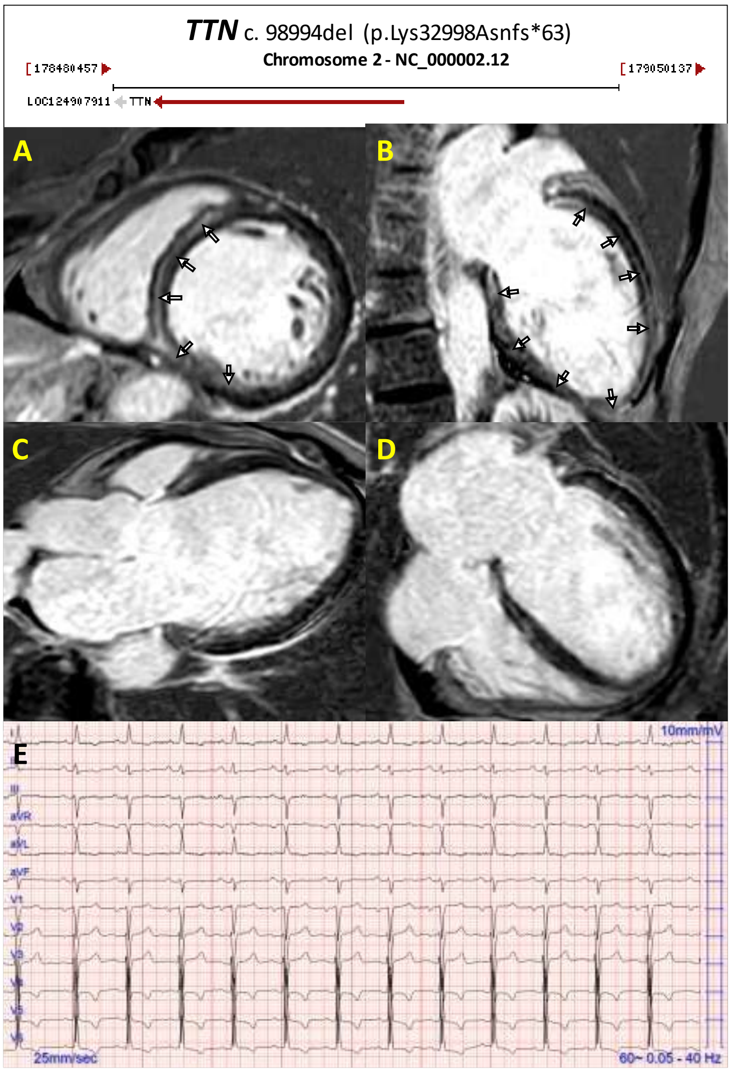


**ALVC in patient with TTN mutation.** ALVC in a patient with severely reduced LV ejection fraction (LVEF: 15%). ECG findings: TWI and RVOT ventricular tachycardias/premature ventricular contractions. CMR: excessive LV trabeculation, midmyocardial LGE at basal/mid septum. ICD implantation. Genetics: ***TTN*** c. 98994del (p.Lys32998Asnfs*63). **A**: CMR LGE short axis; **B**: CMR LGE 2ch; **C**: CMR LGE 3ch; **D**: CMR LGE 4ch; **E**: ECG Sinus with T inversions. *ALVC: Arrhythmogenic Left Ventricular Cardiomyopathy; TWI: T Wave Inversion; RVOT: Right Ventricular Outflow Tract.*

**Supplemental Figure 24**

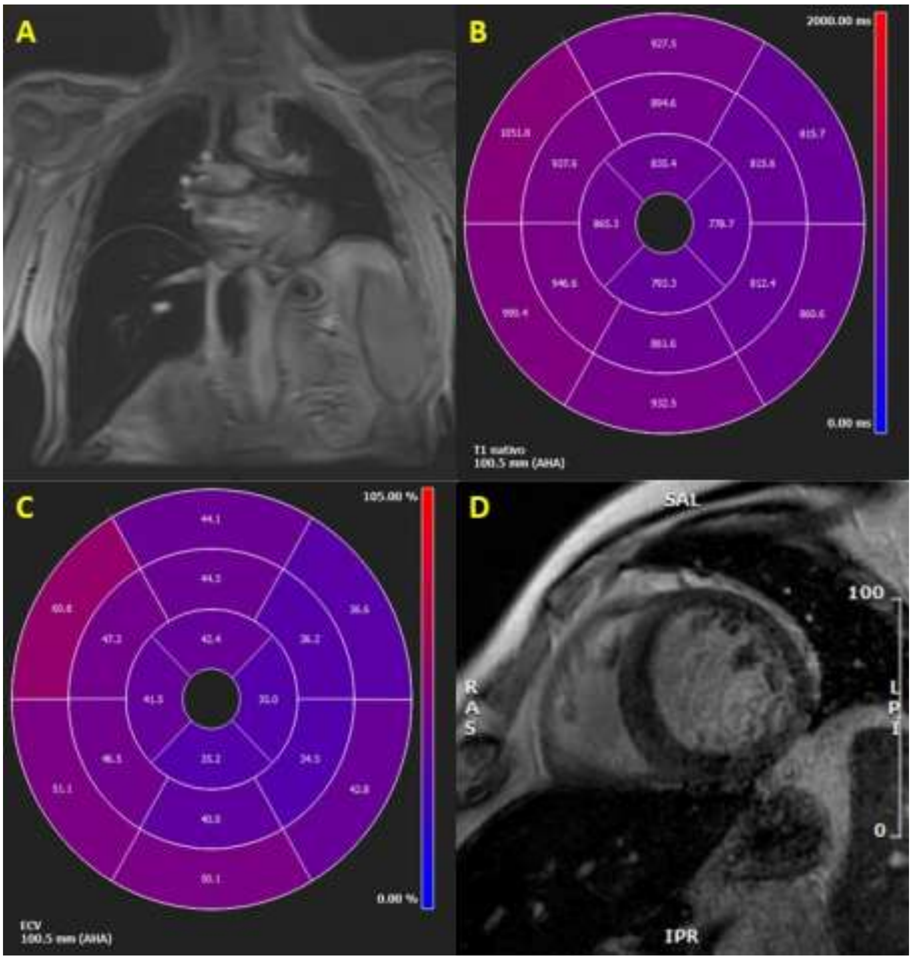


**DCM in patient with haemochromatosis. A**: CMR survey scan; **B**: CMR T1 mapping; **C**: CMR ECV; **D**: CMR LGE short axis. *DCM: Dilated Cardiomyopathy; CMR: Cardiac Magnetic Resonance; ECV: Extracellular Volume; LGE: Late Gadolinium Enhancement.*

**Supplemental Figure 25**

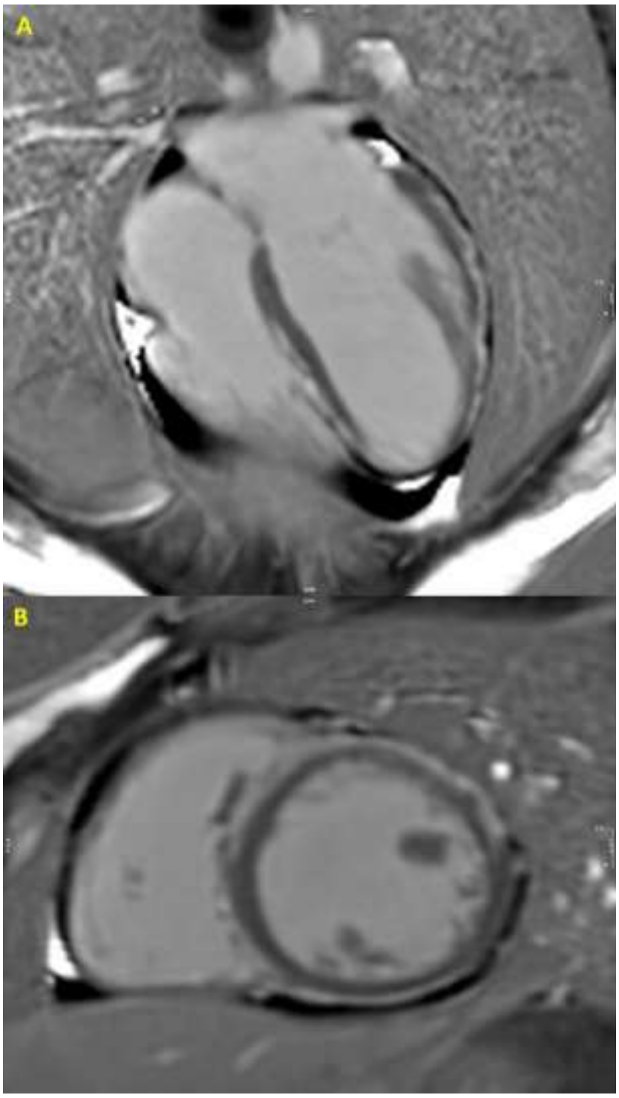


**ALVC in patient with FLNC mutation. A**: CMR 4 chamber cine view; **B**: CMR LGE short axis view. *ALVC: Arrhythmogenic Left Ventricular Cardiomyopathy; CMR: Cardiac Magnetic Resonance; FLNC: Filamin C gene; LGE: Late Gadolinium Enhancement.*

**Supplemental Figure 26**

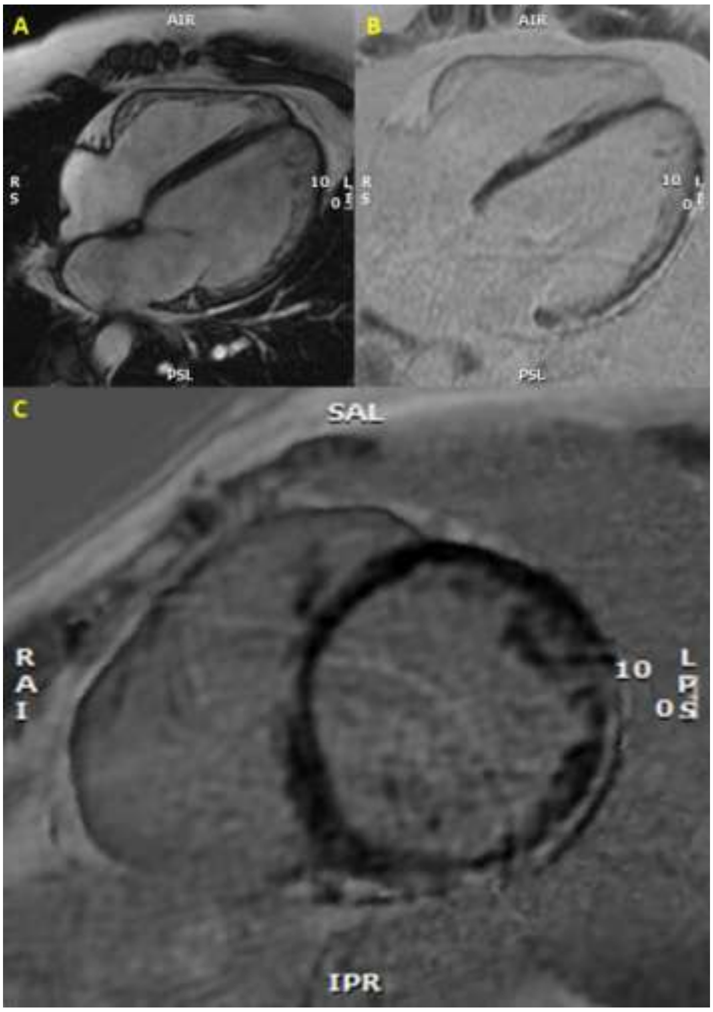


**DCM in patient with Duchenne Muscular Dystrophy. A**: CMR 4 chamber cine view; **B**: CMR LGE 4 chamber view; **C**: CMR LGE short axis view. *DCM: Dilated Cardiomyopathy; CMR: Cardiac Magnetic Resonance; LGE: Late Gadolinium Enhancement.*

**Supplemental Figure 27**

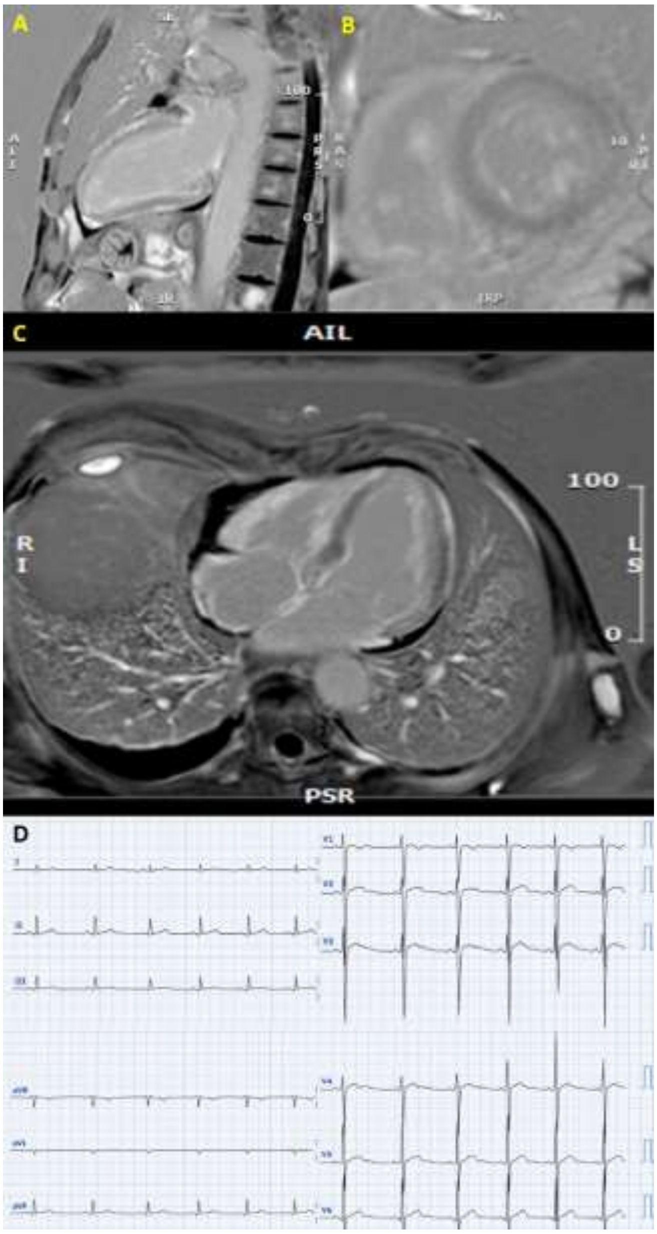


**DCM in patient with hereditary ATTR characterized by cardiac and neurological involvement.** Genetics: ***TTR*** missense variant c.160G (p.Arg54Gly); **A**: CMR 2 chamber LGE view; **B**: CMR LGE short axis view; **C**: CMR LGE 4 chamber view. *ATTR: Transthyretin-related Hereditary Amyloidosis; DCM: Dilated Cardiomyopathy; CMR: Cardiac Magnetic Resonance; LGE: Late Gadolinium Enhancement; TTR: Transthyretin.*

**Supplemental Figure 28:**

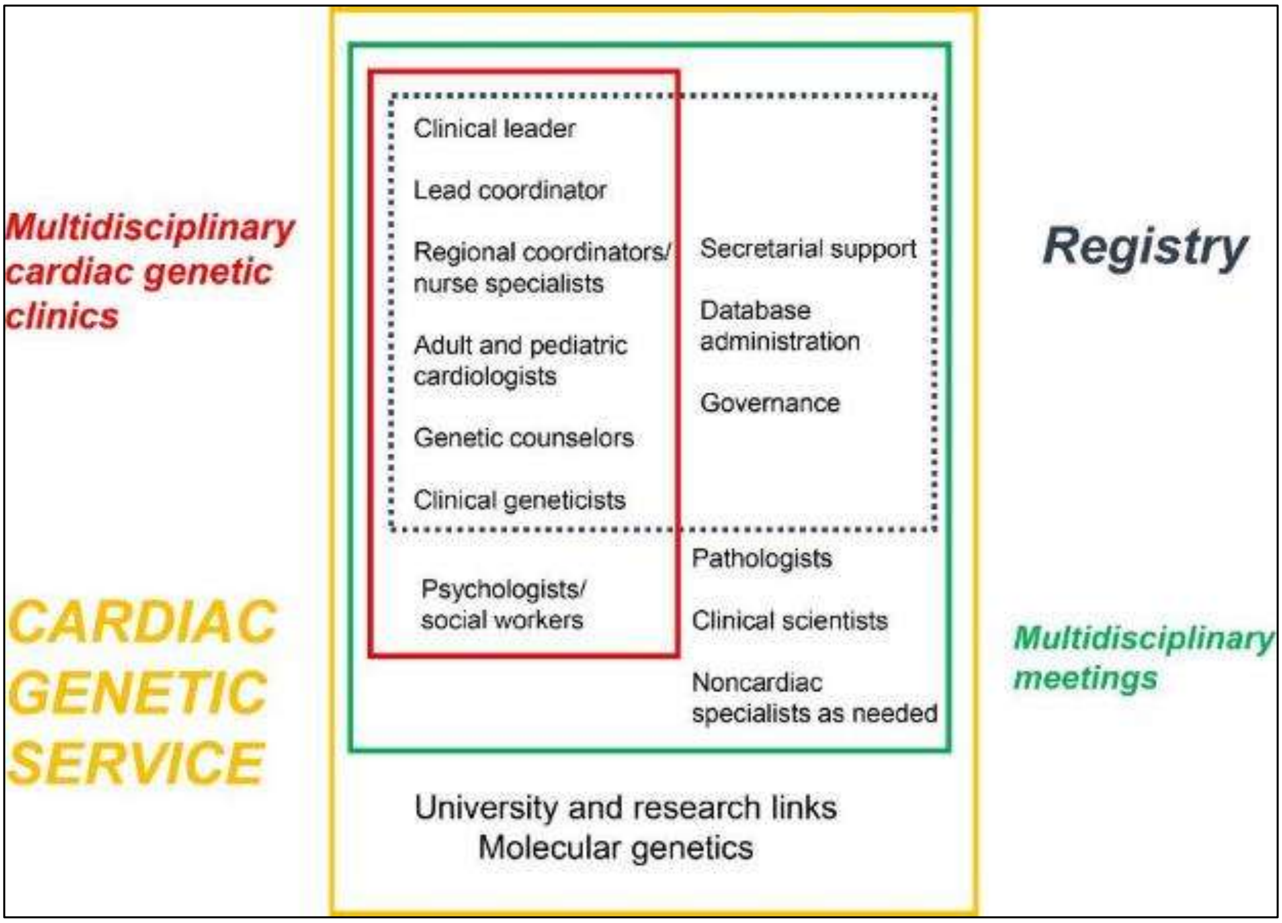


**Cardiac Genetic Service Specialists involved in DCM management.** Genetic testing should be offered to all first-degree relatives if a pathogenic or likely pathogenic variant has been detected in the family because, even in genotype-phenotype-negative members, because a non-monogenic DCM variant could be present. *DCM: Dilated Cardiomyopathy*.

**Supplemental Figure 29**

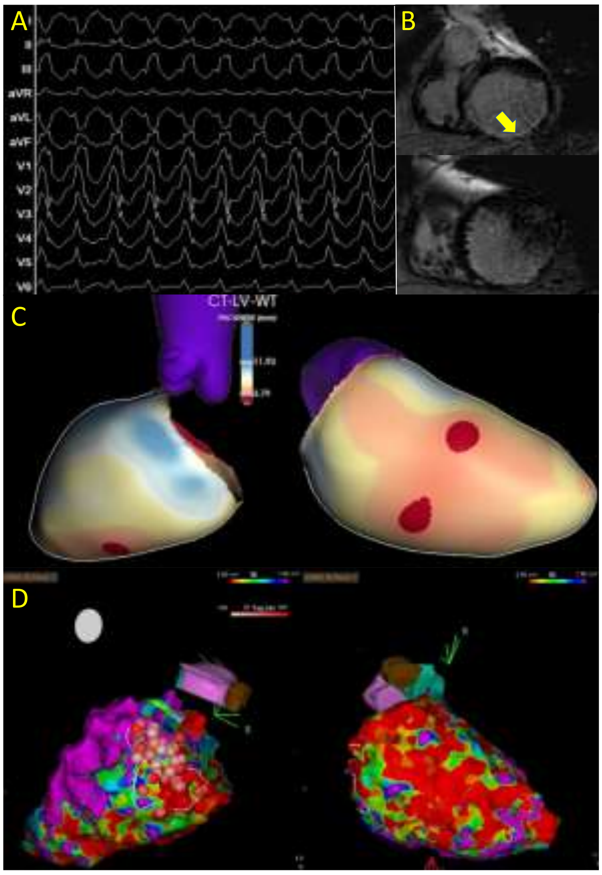


**ADAS case report. A**: VT morphology RBRI, with positive concordance precordial leads, **B**: CMR T1 LGE images showing areas of subendocardial LGE *yellow arrow), and significant device artefact; **C**: ADAS 3D reconstructed model showing left ventricle wall thickness (thicker blue to thinner red), **D**: Electroanatomical map showing Bipolar voltage maps and regions of ablation (red spheres) correlating with transition region from thick to thin on ADAS. *VT: Ventricular Tachycardia; RBRI: Right Bundle Branch Block – Right Inferior; CMR: Cardiac Magnetic Resonance; LGE: Late Gadolinium Enhancement.*

# Supplemental Tables

**Supplemental Table 1. Main clinical and laboratory findings in DCM phenotypes.**

| **Laboratory Finding** | **Dilated Cardiomyopathy** |
|---|---|
| Creatine Kinase increase | Dystrophinopaties, Sarcoglycanopathies, Zaspopathies (LDB3 genes), Laminopathies, etc. |
| High Transferrin saturation or Hyperferritinaemia | Haemochromatosis |
| Lactic Acidosis | Mitochondrial diseases |
| Myoglobinuria | Mitochondrial diseases |
| Leucocytopenia | Mitochondrial diseases (TAZ genes/Barth Syndrome) |
| Myotonia | Myotonic Dystrophy 1 or 2 |
| **Clinical Finding** | **Dilated Cardiomyopathy** |
| Intellectual Disability | Dystrophinopathies, Mithocondrial diseases, FKTN mutations, Myotonic dystrophy |
| Sensorineural deafness | Epicardin mutation, Mithocondrial diseases |
| Visual Impairment | CRYAB (polar cataract), Type 2 Myotonic Dystrophy (subcapsular cataract) |
| Gait disturbance | Dystrophinopaties, Sarcoglycanopathies, Myofibrillar Myopathies |
| Muscle Weakness | Dystrophinopathies, Sarcoglycanopathies, Laminopathies, Myotonic dystrophy, Desminopathy |
| Palpebral Ptosis | Mithocondrial diseases, Myotonic Dystrophy |
| Pigmentation of skin and scars | Haemochromatosis |
| Palmoplantar keratoderma and woolly hair | Carvajal Syndrome, DSP variants |

*Adapted from Rapezzi et al. Diagnostic work-up in cardiomyopathies: bridging the gap between clinical phenotypes and final diagnosis. A position statement from the ESC Working Group on Myocardial and Pericardial Diseases. Eur Heart J. 2013;34(19). CRYAB (Heat-Shock 20 KD Like-Protein); FKTN (Fukuyama-Type Congenital Muscular Dystrophy Protein), TAZ (Tafazzin).*

**Supplemental Table 2. Diagnostic criteria for relatives or familial DCM [adapted from EACVI 2019 Consensus document].**

| Major criteria | Minor criteria |
|---|---|
| Unexplained decrease of LVEF ≤ 50% but > 45%<br><br>Or<br><br>Unexplained LVED dilatation (diameter or volume) according to nomograms (LVED diameter/volume 2 SD + 5% since this more specific echocardiographic criterion was used in studies that demonstrated the predictive impact of isolated dilatation in relatives. | Complete LBBB or AV block (PR ≥ 200 ms or higher degree of AV block);<br><br>Unexplained ventricular arrhythmia (100 ventricular premature beats per hour in 24h or non-sustained ventricular tachycardia, ≥ 3 beats at a rate of ≥ 120 bpm);<br><br>Segmental wall motion abnormalities in the left ventricle in the absence of intraventricular conduction defect;<br><br>LGE of non-ischemic origin on CMR imaging;<br><br>Evidence of non-ischemic myocardial abnormalities (inflammation, necrosis and/or fibrosis) on EMB;<br><br>Presence of serum organ-specific and disease-specific AHA by one or more autoantibody tests. |

*LVEF (Left Ventricular Ejection Fraction); LVED (Left Ventricular End Diastolic); SD (Standard Deviation); LBBB (Left Bundle Branch Block); AV (Atrioventricular); LGE (Late Gadolinium Enhancement); CMR (Cardiovascular Magnetic Resonance); EMB (Endomyocardial Biopsy); AHA (Anti-Histone Antibody).*

**Supplemental Table 3. Main definitions of myocarditis. [adapted from Ammirati et al.]**

| Terms | Definitions |
|---|---|
| **Active Myocarditis** | On the basis of Dallas criteria, active myocarditis indicates the presence of infiltrating inflammatory mononucleated cells and myocyte necrosis, with or without fibrosis, at routine light microscopy evaluation of EMB. |
| **Acute Myocarditis** | Myocarditis with symptoms of recent onset (on average within ≈1 month), generally with increased levels of high-sensitivity troponins, and evidence of edema on CMR if performed within 4 weeks or alternatively positive cardiac FDG-PET imaging (not suggested as routine diagnostic tool). Histologically, it is characterized by an active myocarditis. We propose the term acute presentation when medical attention occurs within 1 month from the symptom onset compared with the previous 3-months interval reported in ESC and AHA scientific statements. The term subacute myocarditis could be used to describe the interval between 1- and 3-months interval from the symptom onset. |
| **Chronic Inflammatory Cardiomyopathy** | Indicates a persistent/chronic myocardial inflammatory condition (symptom onset >1 month) with clinical phenotype of hypokinetic either dilated or non-DCM that can be associated with arrhythmogenic substrate. Histologically, it is generally characterized by myocyte abnormalities (e.g., variations of myocyte diameter), focal or diffuse fibrosis with inflammatory infiltrates. |
| **Chronic Myocarditis** | Defines an ongoing inflammatory process with fibrosis but without myocyte necrosis or myocyte abnormalities. Chronic myocarditis could represent an intermediate stage between acute myocarditis and chronic infl-CMP in patients with persisting myocardial inflammation. This phenotype can be observed in nondilated or mild dilated arrhythmogenic cardiomyopathy or in the setting of an autoimmune disease or syndrome. There is some overlapping with the term subacute myocarditis. |
| **Fulminant Myocarditis** | A working term indicating severe forms of acute myocarditis, with fast evolution and hemodynamic compromise (low-output syndrome or cardiogenic shock) requiring inotropes or MCS. It is a form of acute myocarditis complicated by cardiogenic shock. When performed, EMB often (but not always) shows diffuse inflammatory infiltrates. |
| **Eosinophilic Myocarditis** | Myocarditis characterized by eosinophilic infiltrate at EMB. Peripheral eosinophilia at differential WBC count is suggestive, but it is not always present. |
| **Giant cells Myocarditis** | Myocarditis characterized by large multinuclear cells infiltrating the heart on histology in the absence of well-formed granuloma. It is usually associated with heart dysfunction and is often clinically fulminant. |
| **Lymphocytic Myocarditis** | Myocarditis characterized by small mononuclear cells (CD3+ T lymphocytes) infiltrating the heart. It is the most frequent histological pattern and may or may not be associated with heart dysfunction. It is the histological subtype more often associated with virus-induced myocarditis and immune checkpoint–associated myocarditis. |
| **Infarct-like Myocarditis** | Myocarditis presenting with chest pain and diffuse ST-segment elevation on the ECG that represents about 45.8% of admitted cases of acute myocarditis based on a contemporary registry. The term is misleading since this presentation can be associated with both normal or reduced LVEF, thus without a real prognostic utility. In fact, contrasting results are reported about the outcome of patients with infarct-like myocarditis. Instead, the term uncomplicated myocarditis is preferred to refer to patients with acute myocarditis presenting with chest pain and normal LVEF. |
| **Sarcoidotic Myocarditis** | Patients presenting with an acute myocarditis associated with known or new systemic sarcoidosis. It can also be the clinical presentation of an isolated cardiac sarcoidosis. Sarcoidotic myocarditis is characterized by infiltration by activated macrophages, which in some cases can lead to chronic inflammation and fibrotic replacement with non-necrotizing granulomas. |

*EMB (Endomyocardial Biopsy); CMR (Cardiovascular Magnetic Resonance); FDG-PET (Fluorodeoxyglucose Positron Emission Tomography); ESC (European Society of Cardiology); AHA (American Heart Association); DCM (Dilated Cardiomyopathy); WBC (White Blood Cells); ECG (Electrocardiography); LVEF (Left Ventricular Ejection Fraction).*

**Supplemental Table 4. Clinical and PVC features to identify PVC-Cardiomyopathy [taken from Huizar et al., JACC 2019].**

| | CM resulting in PVCs | PVCs causing CM |
|---|---|---|
| **Patient Characteristics** | Older with known heart disease | Healty otherwise |
| **Comorbidities** | CAD, myocarditis, RV dysplasia | No prior cardiac history |
| **Echocardiogram** | Segmental hypokinesis, LVEF < 25% | Global hypokinesis, LVEF 37±10% |
| **Cardiac MRI (late-gadolinium enhancement)** | Significant scar | Absence or minimal scar burden (≤ 9gm) |
| **PVC Frequency** | < 5,000/24 hrs (< 5%) | ≥ 10,000/24 hours (>10%) |
| **PVC Pattern** | Multifocal | Monomorphic |
| **QRS Morphology** | Non-specific | RVOT/LVOT/Epicardial |
| **Response to PVC suppression** | No change in LV function | Improvement of LV function |

*CAD (Coronary Artery Diserase), CM (Cardiomyopathy), LVEF (Left Ventricular Ejection Fraction), LVOT (Left Ventricular Outflow Tract), MRI (Magnetic Resonance Imaging), PVC (Premature Ventricular Contraction), RV (Right Ventricle), RVOT (Right Ventricular Outflow Tract).*

**Supplemental Table 5. Main Echocardiography parameters in the diagnosis and follow up of DCM [adapted from Mitropoulou et al.]**

| RV dysfunction (2D TTE) | Stress Echocardiography | Speckle Tracking Echocardiography |
|---|---|---|
| FAC < 35%;<br><br>TAPSE < 17 mm;<br><br>Tricuspid annulus S velocity < 9.5 cm/s (derived from tissue Doppler imaging). | Assessment of the presence of contractile reserve, more commonly during dobutamine infusion;<br><br>Screening for preclinical DCM (e.g., asymptomatic LV dysfunction);<br><br>Guiding therapeutic decisions in candidates to cardiac transplantation;<br><br>Identification of inducible myocardial ischemia and viability. | Assessment of cardiac mechanics and deformation via GLS;<br><br>Evaluation of GLS to assess LV dyssynchrony (mechanical dispersion);<br><br>GLS as a good marker of arrythmias in the non-ischemic cardiomyopathy population;<br><br>Early detection of cancer therapy-related cardiac dysfunction;<br><br>Better diagnostic and prognostic performance than LVEF in the assessment of LV dysfunction in relatives of DCM patients during familial screening;<br><br>Most commonly studied parameter for detecting preclinical disease. |

*FAC (Fractional Area Change); TAPSE (Tricuspid Annular Plane Systolic Excursion); GLS (Global Longitudinal Strain).*

**Supplemental Table 6. Nuclear Imaging in the diagnosis of DCM etiology: main tracers and future applications.**

| **Sarcoidosis** | **Inflammatory DCM-like phenotypes** | **Amyloidosis** | **Sympathetic innervation assessment in HF** |
|---|---|---|---|
| SPECT tracers: 99mTc, 201 Tl, 67 Ga are for the detection of the typical sarcoidotic lesions;<br><br>PET tracer: 18F-FDG, for inflammatory areas of sarcoidosis and extra-cardiac areas. | PET tracers: somatostatin receptor-ligands and quantitative radiotracer uptake for implementation of diagnostic accuracy in the diagnosis of inflammatory myocardial disease presenting with a DCM-like phenotype. | SPECT tracers in ATTR amyloidosis: 99mTc-PYP, 99mTc-MDP, 99mTc-DPD have been proposed as accurate diagnostic techniques to single out amyloidotic cardiomyopathy;<br><br>PET: very promising results for amyloid cardiomyopathy, irrespective of the type of amyloid (AL o ATTR). | $^{123}$I-MIBG for tailor treatment and improvement of risk stratification for HF patients, prediction of the applicability of beta-blockade therapy and dose guiding;<br><br>The extent of $^{123}$I-MIBG SPECT appears to be proportional to the risk of ventricular tachyarrhythmia. |

*99mTc (99 metastable Technetium); 201Tl (201 Thallium); 67Ga (67 Gallium); 18F-FDG (18F-fluorodeoxyglucose); PYP (pyrophosphate); MDP (metilene diphosphonate); DPD (3,3-diphospono-1,2-propanodicarboxylic acid); AL (amyloid light chain); ATTR (transthyretin); $^{123}$I-MIBG (123I-metaiodobenzylguanidine).*

**Supplemental Table 7. Main diagnostic and prognostic CMR findings in DCM. [adapted from Merlo et al.]. In *Italics*, the optional techniques.**

| | **Diagnosis:** | **Prognosis:** |
|---|---|---|
| **Cine Imaging:** | Reduced left/biventricular systolic function, possible left/biventricular dilatation | Detection and assessment of right ventricular dysfunction |
| **T2 weighted imaging:** | *Differential diagnosis from "acute inflammatory" cardiomyopathies* | |
| **LGE:** | In up to 30-40% of cases, typically midwall pattern in the interventricular septum. Other patterns are possible (subepicardial pattern in post inflammatory DCM) | Predictor of adverse prognosis. The impact of LGE extension as well as the role of the different LGE locations are still debated |
| **Mapping:** | Altered T1 and ECV mapping reflecting the presence of interstitial fibrosis | Altered T1 and ECV mapping predictors of adverse prognosis in small studies |
| **Featured tracking analysis:** | *May be more accurate in the detection of impaired contractility* | Reduced GLS could correlate with a worse outcome, still needs validation |

*LGE (Late Gadolinium Enhancement); ECV (Extracellular Volume); GLS (Global Longitudinal Strain).*

**Supplemental Table 8. CMR findings associated to DCM phenotypes.**

| Hint | Condition to suspect |
|---|---|
| Short T2* | Haemochromatosis |
| Patchy, midwall LGE hyperenhancement | Post myocarditis, Dystrophinopathy |
| Akinesia/Dyskinesia and LGE at the anterobasal septum or papillary muscles | Sarcoidosis |
| Fatty replacement (T1w FS) within LV wall | ARVC “Left Dominant” |

This figure summarizes the main clinical, laboratoristic and electrocardiographical findings in DCM variants. *CMR: Cardiac Magnetic Resonance; DCM: Dilated Cardiomyopathy.*

**Supplemental Table 9. Main prognostic markers in DCM. [adapted from EACVI 2019 Consensus document].**

| |
|---|
| LA enlargement, RV dilatation, RV contractile dysfunction: risk of death or hospitalization for HF; |
| LV strain as a key independent prognostic marker in DCM; |
| RV strain imaging as a tool of choice to best define the risk of death and hospitalization; |
| LV filling pressure and diastolic function to be assessed by Echocardiography: LA volume, E/A ratio, E velocity deceleration time, e’, E/e’, maximal velocity of tricuspid regurgitation; |
| LA strain is a new promising approach, but still under investigation; |
| Secondary functional MR (Carpentier I + IIIb) detected by TTE is a potentially reversible consequence and aggravator of ventricular remodeling that is incrementally associated with adverse outcome; |
| Contractile reserve and coronary blood flow reserve, by stress echocardiography and/or nuclear imaging measurements, predict RR and functional recovery; |
| Coronary flow reserve in patients with LBBB by Echocardiography; |
| Microvascular dysfunction assessed by PET is associated with poorer outcomes and a higher risk of progression to overt HF and death. |

*LA (Left Atrium); RV (Right Ventricular); HF (Heart Failure); LV (Left Ventricular); DCM (Dilated Cardiomyopathy); MR (Mitral Regurgitation); TTE (Transthoracic Echocardiography); LBBB (Left Bundle Branch Block); PET (Positron Emission Tomography).*

**Supplemental Table 10. ICD recommendations based on genetic variants [adapted from Orphanou et al.].**

| Genetic variant | ICD recommendations |
|---|---|
| *LMNA* | 2/+ of the following risk factors are associated with malignant VAs and SCD → ICD implantation (Class IIa):<br>– Non-sustained ventricular tachycardia (NSVT);<br>– Conduction block;<br>– LVEF < 45% at first evaluation;<br>– Male sex;<br>– Non-missense mutations.<br>– Septal LGE. |
| *BAG3* | Various pathogenic variants are associated with DCM, characterized by high penetrance > 40 years and a high risk of progressive HF:<br>– A point mutation in BAG3 gene causes myofibrillar myopathies with HCM or restrictive cardiomyopathy;<br>– Risk factors for adverse outcomes: male sex, decreased LVEF and enlarged LVEDD. |
| *PLN* | – *R14del PLN* variant carriers show high risk for malignant VAs or end-stage HF;<br>– Sustained or NSVT and LVEF < 45% are independent risk factors for the aforementioned outcomes: high mortality and poor prognosis are noted from late adolescence;<br>– R14del may cause: DCM and ARVC;<br>– In patients with PLN cardiomyopathy and LVEF < 45% or NSVT: ICD implantation (Class IIa). |
| *FLNC* | – Variants associated with skeletal and cardiac myofibrillar myopathies and an overlapping phenotype of left-dominant arrhythmogenic cardiomyopathy and DCM with high risk of malignant VAs and premature SCD;<br>– Phenotype: LV dilatation, LV dysfunction, myocardial fibrosis, inferolateral negative T waves and low QRS voltage;<br>– If LVEF 45%: ICD implantation (Class IIa). |
| *TMEM43* | – Patients with ICD show better survival than those treated with non-ICD management;<br>– Poorer prognosis: male sex;<br>– ECG abnormalities: poor R wave progression, mostly in males. |
| *DSP* | Development of LV dysfunction and may be involved in DCM and left-dominant ARVC. |

*LMNA (Lamin A/C gene); Vas (Ventricular Arrhythmias); SCD (Sudden Cardiac Death); ICD (Implantable Cardiac Defibrillator); LVEF (Left Ventricular Ejection Fraction); LGE (Late Gadolinium Enhancement); BAG3 (BAG Chaperone 3); DCM (Dilated Cardiomyopathy); HF (Heart Failure); HCM (Hypertrophic Cardiomyopathy); LVEDD (Left Ventricular End Diastolic Diameter); PLN (Phospholamban gene); ARVC (Arrhythmogenic Right Ventricular Cardiomyopathy); FLNC (Filamin C gene); LV (Left Ventricular); TMEM43 (Transmembrane Protein 43); DSP (Desmoplakin gene).*

**Supplemental Table 11. Multimodality imaging and specific predictors for ventricular arrhythmias in patients with DCM. [adapted from EACVI 2019 Consensus document].**

| | |
|---|---|
| **Echocardiography:** | – GLS has shown to be a better marker of ventricular arrhythmias and remains a good predictor in patients with relatively preserved EF;<br>– Reversed apical rotation and loss of LV torsion are also associated with significant LV remodeling and more impaired LV function;<br>– Mechanical dispersion has been suggested as a marker of unfavorable arrhythmic outcome; |
| **Cardiovascular Magnetic Resonance:** | - Mid-wall LGE (reflecting replacement fibrosis) is a strong and independent predictor of all-cause mortality, CV death/transplantation and SCD with incremental prognostic value to LVEF;<br>- Patients with mid-wall LGE have a four-fold increased risk of SCD or aborted SCD after correction for other confounders;<br>- Mid-wall fibrosis is an effective prognosticator amongst a wide range of disease severity, including in DCM patients without history of HF (Class B of HF) and in candidates for device(s) treatment;<br>- Newly diagnosed patients without mid-wall LGE are more likely to experience LVRR than those with LGE, irrespective of the severity of clinical status and of LV dilatation and dysfunction at initial evaluation;<br>- DCM patients with mid-wall fibrosis receiving CRT are less likely to exhibit LVRR and have worse clinical outcomes compared to non-LGE patients and these outcomes are similar to those of ischemic cardiomyopathy patients.<br>- Actually, there is no consensus about which cut-off can effectively stratify DCM patients;<br>- RV systolic dysfunction (EF $\leq$ 45%) is a powerful and independent adverse predictor of transplant-free survival and other HF outcomes; |
| **Cardiac Radionuclide imaging techniques:** | **SPECT:**<br>- Impairment of cardiac adrenergic innervation may represent a relevant marker of adverse prognosis, particularly predisposing to the development of malignant ventricular arrhythmias;<br>- Some studies have suggested that a regional 123I-MIBG defect score, derived from SPECT images, may be superior to the H/M ratio in predicting patients' adverse prognosis, highlighting the independent detrimental effect of regional adrenergic innervation heterogeneity.<br>**PET:**<br>- The positron tracers [11C]hydroxyephedrine and [11C]epinephrine permit quantification of the density of sympathetic nerve terminals, while post-synaptic receptor density can be assessed with [11C]CGP12177, which has been shown to independently predict patients' adverse prognosis, particularly related to the incidence of symptomatic HF. |

*GLS (Global Longitudinal Strain); EF (Ejection Fraction); LV (Left Ventricular); LGE (Late Gadolinium Enhancement); CV (Cardiovascular); SCD (Sudden Cardiac Death); DCM (Dilated Cardiomyopathy); HF (Heart Failure); LVRR (Left Ventricular Reverse Remodeling); CRT (Cardiac Resynchronization Therapy); RV (Right Ventricular); SPECT (Single Photon Emission Computed Tomography); H/M (Heart to Mediastinum).*

**Supplemental Table 12. Overall prognostication of adverse events in DCM via CMR. [adapted from Mitropoulou et al.]**

| LGE | T1 mapping | ECV | Feature Tracking Strain | RV systolic function |
|---|---|---|---|---|
| Occurrence of myocardial scar detected by LGE as an independent prognosticator for "hard" events: all-cause mortality, hospitalization, SCD;<br><br>In patients with LGE: increased overall mortality, HF hospitalizations and SCD/aborted SCD;<br><br>LGE presence associated with: CV mortality, ventricular arrhythmic events and rehospitalization for HF;<br><br>Mid-wall fibrosis retains its prognostic value when considered as a continuous variable: the extent, not only the presence, of fibrosis consists of a prognostic marker;<br><br>Myocardial scars allow to identify a subgroup of patients at a higher risk of adverse outcome independently from LVEF. | Higher native T1 values in patients who achieved the primary endpoint of appropriate ICD therapy or sustained ventricular arrhythmia;<br><br>T1 mapping indices (native T1 and ECV) and the extent of LGE, are predictive of all-cause mortality and the composite endpoint of HF mortality and hospitalization. | Myocardial ECV may predict outcomes, as composite of cardiovascular death, HF hospitalizations and appropriate defibrillator intervention, in DCM;<br><br>Mean ECV is strongly associated with major cardiac adverse events in non-ischemic HF patients. | FT parameters as predictors of survival in DCM and refine risk stratification beyond clinical parameters, biomarkers, LVEF and LGE;<br><br>Global and mean longitudinal strain as independent prognostic value surpassing NT-proBNP, LVEF and LGE: a preserved GLS shows excellent prognosis even in patients with LVEF ≤ 35% and in those with LGE;<br><br>GLS incremental in risk stratification with respect to LVEF and LGE extent in ischemic and non-ischemic cardiomyopathy patients;<br><br>GLS is significantly associated with all-cause of death in patients with DCM. | RV dysfunction shows higher risk of all-cause mortality or CT during a median follow up period of 6.8 years;<br><br>RV systolic dysfunction is a significant independent predictor of the primary endpoint of all-cause mortality or CT, as well as secondary outcomes of cardiovascular mortality or CT, HF hospitalization, cardiac death, or CT. |

*LGE (Late Gadolinium Enhancement); SCD (Sudden Cardiac Death); CV (Cardiovascular); HF (Heart Failure); ICD (Implantable Cardioverter Defibrillator); ECV (Extracellular Volume); DCM (Dilated Cardiomyopathy); FT (Feature Tracking); LVEF (Left Ventricular Ejection Fraction); GLS (Global Longitudinal Strain); RV (Right Ventricular); CT (Cardiac Transplantation).*

**Supplemental Table 13. Outcome prediction via CMR [adapted from Mitropoulou et al.]**

| LVRR | Risk of SCD | Response to CRT |
|---|---|---|
| DCM patients without mid-wall LGE are more likely to experience LVRR, irrespective of the severity of clinical status, LV dilatation and dysfunction at initial evaluation;<br><br>Absence of LGE is a very strong predictor of LVRR;<br><br>Native T1 mapping and ECV as additional techniques to help refining the prognostic risk stratification and prediction of LVRR;<br><br>Each technique isolated is probably too weak to accurately predict LVRR: these parameters should be used in combination to elaborate multiparametric scores for LVRR prediction. | LV scar (as detected by LGE on CMR) should guide patient selection for implantation of primary-prevention ICD, with controversial results: Gutman et al. study and DANISH-MRI study;<br><br>The increased risk associated with LV scar may not be associated with shockable ventricular arrythmias;<br><br>Since arrhythmic events occurred more often in patients with scar, then arrhythmic burden does not necessarily entail a net survival benefit from ICD in this population, hinting to alternative potential mechanisms as observed in ischemic cardiomyopathy. | TTE and CMR measures of dyssynchrony should be used in combination;<br><br>In LBBB: short-axis cine imaging to assess radial wall motion to quantify dyssynchrony;<br><br>LGE could help predict clinical response to CRT and guide the lead placement away from areas of scar tissue;<br><br>LV lead positions over scar are associated with a higher risk of cardiovascular death or hospitalization for HF, compared with LV lead positions over viable myocardium. |

*LVRR (Left Ventricular Reverse Remodeling); DCM (Dilated Cardiomyopathy); LGE (Late Gadolinium Enhancement); LV (Left Ventricular); ECV (Extracellular Volume); ICD (Implantable Cardioverter Defibrillator); SCD (Sudden Cardiac Death); CRT (Cardiac Resynchronization Therapy); TTE (Transthoracic Echocardiography); LBBB (Left Bundle Branch Block).*
